# Caputo fractional-order SVIR model for rotavirus: Numerical solutions using Laplace-Adomian decomposition method

**DOI:** 10.1371/journal.pone.0353071

**Published:** 2026-07-21

**Authors:** Monowar Hossain, Mohammed Aman Ullah

**Affiliations:** Department of Mathematics, University of Chittagong, Chattogram, Bangladesh; Princess Sumaya University for Technology, JORDAN

## Abstract

Rotavirus is a leading cause of severe gastroenteritis and diarrheal mortality in children under five years of age, especially in developing countries. Mathematical modeling plays a crucial role in understanding the transmission dynamics of rotavirus and in evaluating the impact of vaccination strategies. In this study, a Caputo fractional-order susceptible–vaccinated–infected–recovered (SVIR) epidemic model is proposed to explore the transmission dynamics of rotavirus while capturing memory and hereditary effects associated with disease progression and immune response. The disease-free and endemic equilibrium points of the model are derived, and the vaccination reproduction number $R_{v}$ is obtained using the next-generation matrix technique. Stability analysis is performed for the disease-free and endemic equilibrium points. In addition, a comparative study for $R_{v}<1$ and $R_{v}>1$ is presented. The sensitivity of the model parameters is computed, and the results are presented graphically. Also, the positivity and boundedness of the solutions are verified to ensure biological feasibility. The approximate solutions of the Caputo fractional-order SVIR model are obtained using the Laplace Adomian Decomposition Method (LADM). The stability, convergence, and error analysis of this well-established method are also studied. To validate the obtained solutions, the method is compared with other methods in the classical-order case. Additionally, the LADM solutions are presented numerically and graphically for different fractional orders, showing that reducing the fractional-order parameters increases memory effects and significantly changes the epidemic dynamics. The numerical and graphical results confirm that the fractional-order framework captures the dynamics of the proposed model more effectively than the corresponding classical integer-order epidemic model.

## 1 Introduction

The mathematical modeling of infectious disease transmission has a long history, beginning with Daniel Bernoulli’s 1766 study on smallpox inoculation, which is considered one of the earliest works in epidemiological modeling [1]. The field was later formalized by Kermack and McKendrick in 1927 through their seminal work, in which they introduced the first deterministic compartmental model describing the dynamics of susceptible, infected, and removed populations [2]. Since then, systems of ordinary differential equation–based compartmental models have become fundamental tools for studying epidemic dynamics, where populations are categorized into epidemiological classes and transitions are controlled by infection, recovery, and immunity processes. Several extensions of the SIR model have been developed, including SIS, SEIR, SEIRS, SEIQR, and SVIR models, to capture additional biological features such as temporary immunity, latent periods, and vaccination effects [3–8]. These models provide a flexible and effective framework for analyzing the spread and control of infectious disease dynamics.

Rotavirus is a major gastrointestinal pathogen that causes acute gastroenteritis and severe diarrhea among children under five years of age worldwide [9,10]. Nearly 90–95% of children are infected with rotavirus before the age of five, with the highest infection rate observed between 4 and 36 months of age [9]. Rotavirus is named after its wheel-like appearance under a microscope, and severe infections are most common in infants aged 6–24 months. Typical symptoms include fever, vomiting, abdominal cramps, and frequent watery diarrhea lasting about one week [11,12]. Among the seven rotavirus species (A–G), only species A, B, and C infect humans, with species A being the most common. Rotavirus transmission occurs primarily through the fecal–oral route via contaminated hands, surfaces, and objects, and may also occur in some cases through respiratory droplets [9,13]. The incubation period is approximately two days, and although reinfections may occur, partial immunity typically leads to reduced disease severity in later infections [9,11]. On a global scale, rotavirus remains a major cause of morbidity and mortality, responsible for about 6% of diarrheal episodes and up to 20% of diarrhea-related deaths among children under five in developing countries, with more than 600,000 deaths annually [9].

Rotavirus is one of the leading causes of diarrhoea in children under five in Bangladesh, with around 10,000 cases per 100,000 children and 2,000–3,000 deaths annually [13–15]. Rotavirus A is the dominant species, with commonly reported genotypes including G1P[8], G2P[4], G3P[8], G4P[8], and G9P[8] [13,14]. Although international vaccines such as Rotarix and RotaTeq are available, rotavirus vaccination has not yet been fully included in Bangladesh’s national immunization program. Vaccine efficacy in Bangladesh is lower than in developed countries, partly because of the circulation of diverse viral strains and frequent mutations [14]. Therefore, studying the prevalence, genetic diversity, and seasonal behavior of rotavirus is essential for evaluating vaccine effectiveness and improving public health policies [13,14].

In this study, we consider the SVIR epidemic model for rotavirus proposed by Omondi et al. [9], which was numerically analyzed in its classical (integer-order) form by Adeniji et al. [16] and Riyapan et al. [17]. More recently, Shah et al. [10] extended this model to a fractional-order framework using the modified Atangana–Baleanu–Caputo fractional derivative. In the present work, we further extend the SVIR rotavirus model by employing the Caputo fractional derivative and obtain semi-analytical solutions using an efficient iterative method. The proposed fractional-order SVIR model can be applied to real epidemiological settings such as rotavirus-endemic regions, where memory and past infection history influence disease transmission. It can help develop vaccination plans and improve forecasts of future epidemic patterns in nations like Bangladesh, making it a valuable tool for public health decision-making in disease control.

In this study, the Caputo fractional derivative is used due to its mathematical convenience, clear biological meaning, and physical relevance, compared with other fractional derivatives like the modified Atangana–Baleanu–Caputo (mABC) derivative. The Caputo derivative is based on a power-law memory structure that is easier to analyse and interpret in epidemiological contexts than the mABC operator, which uses a non-singular and non-local Mittag–Leffler kernel that adds extra model complexity and parameter sensitivity. Additionally, the Caputo formulation allows the use of classical integer-order initial conditions, which are directly obtained from epidemiological data (e.g., initial populations of susceptible, vaccinated, infected, and recovered individuals). Moreover, the Laplace transform is easier to apply in the Caputo fractional-order model than in mABC-based models. Consequently, the Caputo formulation is more suitable for the present SVIR model analysis. A comparative study of the Caputo and Atangana–Baleanu–Caputo fractional derivatives in mathematical biological models can be found in [18,19].

Fractional calculus extends classical calculus to arbitrary-order derivatives and integrals, which allows the modeling of memory effects and hereditary properties because of its nonlocal nature. Time-fractional differential equations use fractional-order time derivatives to capture memory effects, allowing more accurate modeling of real-world processes than classical differential equations. The concept of fractional differentiation originates from Leibniz’s introduction of the notation $d^{n}y/dx^{n}$ and L’Hospital’s 1695 inquiry into the meaning of a derivative of order $n=\frac{1}{2}$, after which many mathematicians, including Riemann, Liouville, Caputo, Hadamard, Weyl, Fourier, Abel, Grünwald, and Letnikov, contributed significantly to the development of fractional calculus [20]. Among the various definitions of fractional derivatives, the Caputo fractional derivative introduced by Michele Caputo is the most widely used because it allows the use of classical initial conditions [21].

In recent years, fractional-order epidemic models have attracted considerable attention because of their ability to represent memory effects and heterogeneity in disease transmission more effectively than classical models. Currently, many researchers have been working in this field, and this research area has gained significant attention [22–28]. In particular, in mathematical biology, homogeneous and heterogeneous fractional-order formulations have been developed to describe how different population groups respond differently to infection and vaccination processes [29,30]. The incorporation of fractional derivatives allows the present infection dynamics to depend continuously on past states, reflecting the long-term influence of prior exposure, immunity, and treatment history. This feature is especially important in viral transmission, where immunity wanes over time and previous infection levels influence future outbreaks. Compared to integer-order models, which assume instantaneous responses, fractional models provide a smoother and more flexible representation of disease progression. In this study, the Caputo fractional derivative is employed due to its physical interpretability and its capability to describe memory effects more effectively in vaccination and infection dynamics.

Recently, the analytical and numerical study of epidemiological models describing infectious disease dynamics has received considerable interest. Numerous semi-analytical and numerical techniques have been developed and used because the majority of epidemic models are nonlinear and rarely yield closed-form solutions. These techniques include the homotopy perturbation method [31] and the Adomian decomposition method [32], the differential transform method [16], the variational iteration method [33], the fourth-order Runge–Kutta method [34], the Runge–Kutta–Fehlberg method [16], the Laplace Adomian decomposition method [16], the homotopy perturbation transform method [30], and the Yang transform decomposition method [30], and many others.

Among these techniques, the LADM has gained particular popularity due to its efficiency, rapid convergence, and ability to handle strong nonlinearities. In recent years, this method has been successfully applied to both classical and fractional-order epidemic models, including the HIV infection of CD4^+^ T cells [34], coronavirus models [35,36], the SEIR measles model [37], Lassa fever [38], smoking dynamics [39], dengue fever [40], childhood diseases [41], monkeypox [42], Diabetes [43] and chickenpox [44]. Motivated by these successful applications, in this paper we employ the Laplace Adomian decomposition method to analyze a fractional-order rotavirus transmission model.

The Laplace Adomian Decomposition Method was introduced by Khuri [45] as a combination of the Laplace transform and the Adomian decomposition method. It is a well-known technique for solving linear and nonlinear differential equations of both classical and fractional order, and it provides accurate solutions in the form of a rapidly convergent series. In this method, a differential equation is first transformed into an algebraic equation using the Laplace transform, and then the nonlinear terms are decomposed in terms of Adomian polynomials [28,46]. This method is easy to apply, and does not require linearization, discretization, perturbation, or restrictive assumptions.

The rest of this paper is organized as follows. The *Mathematical Preliminaries* section presents the mathematical preliminaries and basic definitions of fractional calculus. The *Model Formulation* section introduces the proposed fractional-order SVIR rotavirus model. The *Some Fundamental Results about the Model* section discusses the fundamental properties and results of the model. The *Laplace Adomian Decomposition Method* section describes the LADM used to obtain the approximate solution of the model, while the *Stability, Convergence and Error Analysis* section provides the stability, convergence, and error analysis of the proposed method. The *Numerical Solution* section presents the numerical simulations and solutions of the model, followed by the *Results and Discussion* section, which discusses the obtained results in detail. Finally, the *Conclusion* section summarizes the main findings and conclusions of the study.

## 2 Mathematical preliminaries

This section presents fundamental concepts of fractional calculus essential for analyzing the fractional-order SVIR model.

**Definition 1 (Riemann-Liouville Fractional Integral).** *For a function* 𝒮(t) *and real number* α>0*, the Riemann-Liouville fractional integral of order* α *is defined as* [21]*:*


$$\begin{array}{c} J^{\alpha }𝒮(t)=\frac{1}{\Gamma (\alpha )}\int_{0}^{t}(t-\tau {)}^{\alpha -1}𝒮(\tau )\;d\tau ,\;t>0, \end{array}$$ (1)

*where* Γ(·) *denotes the Gamma function.*

**Definition 2 (Caputo Fractional Derivative).** *For a function* $𝒮(t)\in C^{n}[0,\infty )$ *and real number* α>0*, the Caputo fractional derivative of order* α *is defined as* [21]*:*


$$\begin{array}{c} {\;}^{C}D_{t}^{\alpha }𝒮(t)=\left\{ \begin{array}{cc} \frac{1}{\Gamma (n-\alpha )}\int_{0}^{t}(t-\tau {)}^{n-\alpha -1}{𝒮}^{\left(n\right)}(\tau )\;d\tau , & n-1<\alpha <n,\\ \frac{d^{n}}{dt^{n}}𝒮(t), & \alpha =n, \end{array}\right. \end{array}$$ (2)

*where* n∈ℕ *and* Γ(·) *represents the gamma function.*

*For power functions, the Caputo derivative satisfies:*


$$\begin{array}{c} {\;}^{C}D_{t}^{\alpha }\;t^{m}=\left\{ \begin{array}{cc} \frac{\Gamma (m+1)}{\Gamma (m-\alpha +1)}t^{m-\alpha }, & m>n-1,\\ 0, & m\leq n-1, \end{array}\right. \end{array}$$ (3)

*where* n−1<α≤n *and* m∈ℝ.

*Equivalently, in factorial form:*


$$\begin{array}{c} {\;}^{C}D_{t}^{\alpha }\;t^{m}=\left\{ \begin{array}{cc} \frac{m!}{(m-\alpha )!}\;t^{m-\alpha }, & m>n-1,\\ 0, & m\leq n-1, \end{array}\right. \end{array}$$ (4)

*where* n−1<α≤n, m∈ℝ*, and the factorial notation m! denotes* Γ(m+1).

**Definition 3 (Mittag–Leffler Function).** *The one-parameter Mittag–Leffler function is defined as [*21*]:*


$$\begin{array}{c} E_{a}(z)=\sum_{k=0}^{\infty }\frac{z^{k}}{\Gamma (ak+1)},\;a\in {\mathbb{R}}^{+},\;z\in \mathbb{C}. \end{array}$$ (5)

**Definition 4 (Laplace Transform).** *The Laplace transform of* 𝒮(t) *is defined as:*


$$\begin{array}{c} ℒ[𝒮(t)]=S(s)=\int_{0}^{\infty }e^{-st}𝒮(t)\;dt, \end{array}$$ (6)

*where S(s) denotes the Laplace transform of* 𝒮(t).

**Definition 5 (Laplace Transform of Caputo Derivative).** *For* m−1<α≤m *where* m∈ℕ*, the Laplace transform of the Caputo fractional derivative satisfies [*21*]:*


$$\begin{array}{c} ℒ\left\{{\;}^{C}D_{t}^{\alpha }𝒮(t)\right\}=s^{\alpha }S(s)-\sum_{k=0}^{m-1}s^{\alpha -k-1}{𝒮}^{\left(k\right)}(0), \end{array}$$ (7)

*where S(s) denotes the Laplace transform of* 𝒮(t) *and* ${𝒮}^{\left(k\right)}(0)$ *are the initial conditions.*

For 0<α<1, the Caputo fractional derivative can be expressed in memory kernel form as ${\;}^{C}D_{t}^{\alpha }𝒮(t)=\int_{0}^{t}K_{\alpha }(t-\tau )\;{𝒮}^{\prime }(\tau )\;d\tau$, where $K_{\alpha }(t-\tau )=(t-\tau {)}^{-\alpha }/\Gamma (1-\alpha )$ denotes the memory kernel of the system, which assigns a power-law weighting to the entire past history on [0,*t*], resulting in non-local and long-memory behavior. The Caputo fractional derivative is suitable for initial value problems in fractional differential equations because the Caputo deriva*t*ive of a constant is zero and the initial conditions are expressed in terms of standard derivatives at *t* = 0, which have clear physical interpretations.

## 3 Model formulation

In this study, we consider the SVIR epidemic model of rotavirus proposed by Omondi *et al.* [9] and subsequently studied by Adeniji *et al.* [16] for the classical case. The total human population at time *t*, denoted by 𝒩(t)=𝒮(t)+𝒱(t)+ℐ(t)+ℛ(t), is divided into susceptible, vaccinated, infected, and recovered compartments. Recruitment into the susceptible and vaccinated classes occurs at the rates (1−π)λ and πλ, respectively. Susceptible individuals become infected through effective contact with infected individuals at the rate β𝒮ℐ, where β denotes the effective contact rate. Vaccination of susceptible individuals occurs at rate κ. Vaccine-induced immunity wanes at rate ω. The parameter 0<ξ<1 measures the reduction in the risk of infection due to vaccination. Infected individuals recover at rate δ, experience disease-induced mortality at rate σ, and all individuals are subject to natural death at rate μ.

Based on these assumptions, the classical integer-order model is described by the system


$$\begin{array}{cc} \frac{d𝒮}{dt} & =(1-\pi )\lambda -\beta 𝒮ℐ-\kappa 𝒮+\omega 𝒱-\mu 𝒮,\\ \frac{d𝒱}{dt} & =\pi \lambda +\kappa 𝒮-\xi \beta 𝒱ℐ-(\omega +\mu )𝒱,\\ \frac{dℐ}{dt} & =\beta 𝒮ℐ+\xi \beta 𝒱ℐ-(\sigma +\delta +\mu )ℐ,\\ \frac{dℛ}{dt} & =\delta ℐ-\mu ℛ. \end{array}$$ (8)

To capture memory and hereditary effects inherent in disease transmission and immune response mechanisms, we generalize the above system by introducing fractional-order derivatives in the sense of Caputo. Different fractional orders are assigned to each epidemiological compartment to allow heterogeneous memory effects in the population. Let $\alpha_{𝒮}$, $\alpha_{𝒱}$, $\alpha_{ℐ}$, and $\alpha_{ℛ}$ denote the fractional orders corresponding to the susceptible, vaccinated, infected, and recovered classes, respectively, with $0<\alpha_{𝒮},\alpha_{𝒱},\alpha_{ℐ},\alpha_{ℛ}\leq 1$.

The resulting fractional-order model is given by


$$\begin{array}{cc} {\;}^{C}D_{t}^{\alpha_{𝒮}}𝒮(t) & =(1-\pi )\lambda -\beta 𝒮ℐ-\kappa 𝒮+\omega 𝒱-\mu 𝒮,\\ {\;}^{C}D_{t}^{\alpha_{𝒱}}𝒱(t) & =\pi \lambda +\kappa 𝒮-\xi \beta 𝒱ℐ-(\omega +\mu )𝒱,\\ {\;}^{C}D_{t}^{\alpha_{ℐ}}ℐ(t) & =\beta 𝒮ℐ+\xi \beta 𝒱ℐ-(\sigma +\delta +\mu )ℐ,\\ {\;}^{C}D_{t}^{\alpha_{ℛ}}ℛ(t) & =\delta ℐ-\mu ℛ. \end{array}$$ (9)

The fractional-order parameters $\alpha_{S}$, $\alpha_{V}$, $\alpha_{I}$, and $\alpha_{R}$ are introduced to incorporate memory and hereditary effects into the SVIR epidemic system. In fractional-order epidemic models, the current state of the disease depends not only on the present conditions but also on the past history of the transmission process. The parameters $\alpha_{S}$, $\alpha_{V}$, $\alpha_{I}$, and $\alpha_{R}$ represent the memory effects associated with the susceptible, vaccinated, infected, and recovered populations, respectively. Lower fractional orders indicate stronger memory effects, which slow the system dynamics compared to the classical case. This means that the past states have a longer effect on the current epidemic behavior, leading to a slower decay of the infection, delayed recovery. When $\alpha_{S}=\alpha_{V}=\alpha_{I}=\alpha_{R}=1$, the fractional-order system reduces to the corresponding classical integer-order model without memory effects. Therefore, the proposed model generalized the classical epidemic model, providing a more flexible mathematical structure to describe the dynamics of disease transmission.

**Fig 1:**
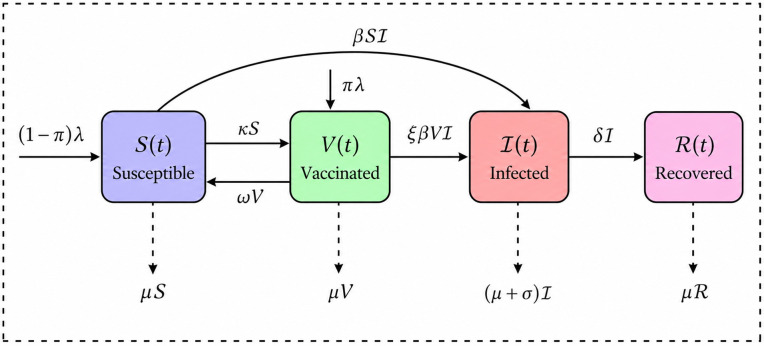
Compartmental flowchart of the SVIR model.

Fig 1 shows the flow diagram of the SVIR model, illustrating the transitions between susceptible, vaccinated, infected, and recovered compartments. The epidemiological parameters, their biological interpretations, numerical values, and units employed in the SVIR model simulations are summarized in Table 1.

**Table 1:** Parameters and their values for the SVIR model [10].

Parameter	Description	Value	Unit
λ	Total recruitment rate	0.4109	people/day
π	Recruitment rate of vaccinated individuals	0.001884	people/day
β	Effective contact rate	0.0001	day^−1^
κ	Vaccination rate	0.001884	day^−1^
ω	Vaccine efficacy waning rate	0.002778	day^−1^
ξ	Expected decrease in the risk of infection	0.001	day^−1^
δ	Rate of flow into the removed class	0.099	day^−1^
σ	Rotavirus-induced deaths	0.00004466	day^−1^
μ	Natural death rate	0.00002537	day^−1^

## 4 Some fundamental results about the model

### 4.1 Feasible region

The total population at time *t* is given by


$$\begin{array}{c} 𝒩(t)=𝒮(t)+𝒱(t)+ℐ(t)+ℛ(t). \end{array}$$ (10)

For feasibility analysis, we consider the homogeneous fractional-order case where $\alpha_{𝒮}=\alpha_{𝒱}=\alpha_{ℐ}=\alpha_{ℛ}=\alpha$. Applying the fractional derivative operators to 𝒩(t) and summing the equations of system (9) yields:


$$\begin{array}{c} {\;}^{C}D_{t}^{\alpha }𝒩(t)=\lambda -\mu 𝒩-\sigma ℐ. \end{array}$$ (11)

where 0<α≤1. In the disease–free case (ℐ=0), this reduces to


$$\begin{array}{c} {\;}^{C}D_{t}^{\alpha }𝒩(t)=\lambda -\mu 𝒩(t). \end{array}$$ (12)

Applying the Laplace transform and letting t→∞, we obtain the steady–state total population. Hence, the feasible region of the model is


$$\begin{array}{c} \Psi =\{(𝒮,𝒱,ℐ,ℛ)\in {\mathbb{R}}_{+}^{4}:𝒩\leq \frac{\lambda }{\mu }\}. \end{array}$$ (13)

### 4.2 Equilibrium points and basic reproduction number

Setting the left–hand sides of system (9) equal to zero, the equilibrium points are obtained.

At the disease–free equilibrium, ℐ=0 and ℛ=0. Hence,


$$\begin{array}{cc} 0 & =(1-\pi )\lambda -\kappa {𝒮}^{0}-\mu {𝒮}^{0}+\omega {𝒱}^{0},\\ 0 & =\pi \lambda +\kappa {𝒮}^{0}-(\omega +\mu ){𝒱}^{0},\\ 0 & ={ℐ}^{0},\\ 0 & ={ℛ}^{0}. \end{array}$$ (14)

By using the relation 𝒮+𝒱=λ/μ, the disease-free equilibrium of the model can be determined by substituting ${𝒱}^{0}=\frac{\pi \lambda +\kappa {𝒮}^{0}}{\omega +\mu }$ (obtained from the second equation of (14)) into the first equation of (14) and solving for ${𝒮}^{0}$ and ${𝒱}^{0}$. Hence,


$$\begin{array}{c} {𝒮}^{0}=\frac{\lambda (\mu -\pi \mu +\omega )}{\mu (\kappa +\mu +\omega )},\;{𝒱}^{0}=\frac{\lambda (\pi \mu +\kappa )}{\mu (\kappa +\omega +\mu )}. \end{array}$$ (15)

Therefore, the disease–free equilibrium is


$$\begin{array}{c} {ℰ}^{0}=\left(\frac{\lambda (\mu -\pi \mu +\omega )}{\mu (\kappa +\mu +\omega )},\frac{\lambda (\pi \mu +\kappa )}{\mu (\kappa +\omega +\mu )},0,0\right). \end{array}$$ (16)

The endemic equilibrium ${ℰ}^{*}=({𝒮}^{*},{𝒱}^{*},{ℐ}^{*},{ℛ}^{*})$ is computed as


$$\begin{array}{cc} 0 & =(1-\pi )\lambda -\beta {𝒮}^{*}{ℐ}^{*}-\kappa {𝒮}^{*}+\omega {𝒱}^{*}-\mu {𝒮}^{*},\\ 0 & =\pi \lambda +\kappa {𝒮}^{*}-\xi \beta {𝒱}^{*}{ℐ}^{*}-(\omega +\mu ){𝒱}^{*},\\ 0 & =\beta {𝒮}^{*}{ℐ}^{*}+\xi \beta {𝒱}^{*}{ℐ}^{*}-(\sigma +\delta +\mu ){ℐ}^{*}.\\ 0 & =\delta {ℐ}^{*}-\mu {ℛ}^{*}. \end{array}$$ (17)

$$\begin{array}{cc} {𝒮}^{*}= & \frac{\left(\sigma +\delta +\mu )(\xi \beta {ℐ}^{*}+\mu +\omega +\kappa )-\xi (\beta \pi \lambda +\kappa (\sigma +\delta +\mu )\right)}{\beta (\xi \beta {ℐ}^{*}+\mu +\omega +\kappa )},\\ {𝒱}^{*}= & \frac{\beta \pi \lambda +\kappa (\sigma +\delta +\mu )}{\beta (\xi \beta {ℐ}^{*}+\mu +\omega +\kappa )},\\ {ℐ}^{*}= & \frac{\sqrt{A}-\beta \kappa (-\beta \lambda +\xi (\mu +1)(\sigma +\delta +\mu )+(\mu +\omega )(\sigma +\delta +\mu ))}{2\beta^{2}\xi (\sigma +\delta +\mu )},\\ {ℛ}^{*}= & \frac{\delta }{\mu }{ℐ}^{*}. \end{array}$$

where


$$\begin{array}{cc} A= & \beta^{2}(\kappa^{2}(-\beta \lambda +\xi (\mu +\omega )(\sigma +\delta +\mu )+(\mu +\omega )(\sigma +\delta +\mu ){)}^{2}-\beta \lambda (\kappa \xi +\mu +\omega ))\\ & -4\xi (\sigma +\delta +\mu )(\mu (\kappa (\sigma +\delta +\mu )-\xi \lambda \pi +\sigma (\mu +\omega )+\lambda \pi +\mu^{2}+\mu \sigma +\mu \omega +\sigma \omega )) \end{array}$$

Now, using the next-generation matrix method [47], the basic reproduction number with vaccination is derived as follows.

We consider the infected compartments of the system, which is only ℐ. The vector of new infections ℱ and the vector of transfer terms 𝒢 are given by:


$$\begin{array}{c} ℱ=[\begin{array}{c} \beta 𝒮ℐ+\xi \beta 𝒱ℐ \end{array}],\;𝒢=[\begin{array}{c} (\sigma +\delta +\mu )ℐ \end{array}]. \end{array}$$ (18)

Then, the Jacobian matrices *F* and *G* evaluated at the disease-free equilibrium ${ℰ}^{0}$ are:


$$\begin{array}{c} F=[\begin{array}{c} \beta {𝒮}^{0}+\xi \beta {𝒱}^{0} \end{array}],\;G=[\begin{array}{c} \sigma +\delta +\mu \end{array}]. \end{array}$$ (19)

The next-generation matrix is $FG^{-1}$, we compute:


$$\begin{array}{c} FG^{-1}=[\begin{array}{c} \beta {𝒮}^{0}+\xi \beta {𝒱}^{0} \end{array}]\times [\begin{array}{c} \frac{1}{\sigma +\delta +\mu } \end{array}]=[\begin{array}{c} \frac{\beta {𝒮}^{0}+\xi \beta {𝒱}^{0}}{\sigma +\delta +\mu } \end{array}]. \end{array}$$ (20)

Therefore, the basic reproduction number with vaccination, $R_{v}$, is given by:


$$\begin{array}{c} R_{v}=\frac{\beta {𝒮}^{0}+\xi \beta {𝒱}^{0}}{\sigma +\delta +\mu }, \end{array}$$

where *S*^0^ and *V*^0^ are the disease-free equilibrium values of the susceptible and vaccinated populations, respectively. Substituting


$$\begin{array}{c} {𝒮}^{0}=\frac{\lambda (\mu -\pi \mu +\omega )}{\mu (\kappa +\mu +\omega )},\;{𝒱}^{0}=\frac{\lambda (\pi \mu +\kappa )}{\mu (\kappa +\omega +\mu )}, \end{array}$$

we obtain


$$\begin{array}{c} R_{v}=\frac{\beta \lambda \left[\mu (1-\pi +\xi \pi )+\omega +\xi \kappa \right]}{\mu (\sigma +\delta +\mu )(\kappa +\mu +\omega )}. \end{array}$$ (21)

In the absence of vaccination (π=κ=ω=0 and ξ=1), this reduces to the classical basic reproduction number


$$\begin{array}{c} R_{0}=\frac{\beta \lambda }{\mu (\sigma +\delta +\mu )}. \end{array}$$ (22)

Thus, the vaccination reproduction number can also be expressed as


$$\begin{array}{c} R_{v}=R_{0}\cdot \frac{\mu (1-\pi +\xi \pi )+\omega +\xi \kappa }{\kappa +\mu +\omega }. \end{array}$$ (23)

Since 0<ξ≤1, it follows that $R_{v}\leq R_{0}$ whenever vaccination is implemented (π>0 or κ>0), demonstrating the effectiveness of the vaccination program in reducing disease transmission.

### 4.3 Stability analysis

For fractional-order systems with Caputo derivative of order α∈(0,1], the local asymptotic stability of an equilibrium point is determined by the eigenvalues of the Jacobian matrix evaluated at that equilibrium. Specifically, the equilibrium is locally asymptotically stable if and only if all eigenvalues $\lambda_{i}$ satisfy [48,49]:


$$\begin{array}{c} |\text{arg}(\lambda_{i})|>\frac{\alpha \pi }{2},\;i=1,2,\ldots ,n. \end{array}$$ (24)

When α=1, this condition reduces to the classical integer-order stability condition Re(λi)<0.

#### 4.3.1 Local stability of the disease-free equilibrium.

The Jacobian matrix *J* of system (9) evaluated at the disease-free equilibrium ${ℰ}^{0}$ is:


$$\begin{array}{c} J({ℰ}^{0})=[\begin{array}{cccc} -\kappa -\mu & \omega & -\beta {𝒮}^{0} & 0\\ \kappa & -(\omega +\mu ) & -\xi \beta {𝒱}^{0} & 0\\ 0 & 0 & \beta {𝒮}^{0}+\xi \beta {𝒱}^{0}-(\sigma +\delta +\mu ) & 0\\ 0 & 0 & \delta & -\mu \end{array}]. \end{array}$$ (25)

To find the eigenvalues, we solve $\det(J({ℰ}^{0})-\lambda I)=0$:


$$\begin{array}{c} \det[\begin{array}{cccc} -\kappa -\mu -\lambda & \omega & -\beta {𝒮}^{0} & 0\\ \kappa & -(\omega +\mu )-\lambda & -\xi \beta {𝒱}^{0} & 0\\ 0 & 0 & \beta {𝒮}^{0}+\xi \beta {𝒱}^{0}-(\sigma +\delta +\mu )-\lambda & 0\\ 0 & 0 & \delta & -\mu -\lambda \end{array}]=0. \end{array}$$ (26)

The matrix is block triangular (the lower-left 2×2 block is zero). Therefore, the determinant is the product of the determinants of the diagonal blocks:


$$\begin{array}{c} \det(J({ℰ}^{0})-\lambda I)=\det(B_{1})\cdot \det(B_{2})=0, \end{array}$$ (27)

where


$$\begin{array}{c} B_{1}=[\begin{array}{cc} -\kappa -\mu -\lambda & \omega \\ \kappa & -(\omega +\mu )-\lambda \end{array}],\;B_{2}=[\begin{array}{cc} \beta {𝒮}^{0}+\xi \beta {𝒱}^{0}-(\sigma +\delta +\mu )-\lambda & 0\\ \delta & -\mu -\lambda \end{array}]. \end{array}$$ (28)

The eigenvalues of $J({ℰ}^{0})$ are:


$$\begin{array}{c} \lambda_{1}=-\mu , \end{array}$$ (29)

$$\begin{array}{c} \lambda_{2}=-\mu , \end{array}$$ (30)

$$\begin{array}{c} \lambda_{3}=-(\kappa +\mu +\omega ), \end{array}$$ (31)

$$\begin{array}{c} \lambda_{4}=\beta {𝒮}^{0}+\xi \beta {𝒱}^{0}-(\sigma +\delta +\mu ). \end{array}$$ (32)

The first three eigenvalues are real and negative. The fourth eigenvalue simplifies using the expression for $R_{v}$:


$$\begin{array}{c} \lambda_{4}=(\sigma +\delta +\mu )\left(\frac{\beta {𝒮}^{0}+\xi \beta {𝒱}^{0}}{\sigma +\delta +\mu }\right)-(\sigma +\delta +\mu )=(\sigma +\delta +\mu )(R_{v}-1). \end{array}$$ (33)

Thus, the eigenvalues are:


$$\begin{array}{c} \lambda_{1}=\lambda_{2}=-\mu ,\;\lambda_{3}=-(\kappa +\mu +\omega ),\;\lambda_{4}=(\sigma +\delta +\mu )(R_{v}-1). \end{array}$$ (34)

For the fractional-order system with derivative order α∈(0,1], we analyze the argument condition:

- For $\lambda_{1},\lambda_{2},\lambda_{3}$: Since these are negative real numbers, $\text{arg}(\lambda_{i})=\pi$. Thus:
  $$\begin{array}{c} |\text{arg}(\lambda_{i})|=\pi >\frac{\alpha \pi }{2}\;\text{for all }\alpha \in (0,1], \end{array}$$
- For $\lambda_{4}$, if $R_{v}<1$ then $\lambda_{4}<0$ and the stability condition $|\text{arg}(\lambda_{4})|>\alpha \pi /2$ holds; if $R_{v}>1$ then $\lambda_{4}>0$ and the condition fails for any α∈(0,1], implying instability. When ξ=1 or π=κ=0, we obtain $R_{v}=R_{0}$. Consequently, if *R*_0_ < 1, the disease-free equilibrium is locally asymptotically stable, whereas if *R*_0_ > 1, it is unstable.

For integer-order compartmental models, it is well-known that the basic reproduction number determines the local stability of the disease-free equilibrium: if *R*_0_ < 1, the disease-free equilibrium is locally asymptotically stable; if *R*_0_ > 1, it is unstable [47]. Our fractional-order analysis confirms that this threshold behavior extends to Caputo fractional derivatives of order α∈(0,1], with the disease-free equilibrium is locally asymptotically stable when *R*_0_ < 1 and unstable when *R*_0_ > 1. This approach is comparable to the stability analysis conducted for rotavirus infection in breastfed and non-breastfed children [11].

#### 4.3.2 Local stability of the endemic equilibrium.

The endemic equilibrium ${ℰ}^{*}=({𝒮}^{*},{𝒱}^{*},{ℐ}^{*},{ℛ}^{*})$ exists uniquely when $R_{v}>1$. The Jacobian matrix evaluated at ${ℰ}^{*}$ is:


$$\begin{array}{c} J({ℰ}^{*})=[\begin{array}{cccc} -\beta {ℐ}^{*}-\kappa -\mu & \omega & -\beta {𝒮}^{*} & 0\\ \kappa & -\xi \beta {ℐ}^{*}-(\omega +\mu ) & -\xi \beta {𝒱}^{*} & 0\\ \beta {ℐ}^{*} & \xi \beta {ℐ}^{*} & \beta {𝒮}^{*}+\xi \beta {𝒱}^{*}-(\sigma +\delta +\mu ) & 0\\ 0 & 0 & \delta & -\mu \end{array}]. \end{array}$$ (35)

Using the equilibrium condition:


$$\begin{array}{c} \beta {𝒮}^{*}+\xi \beta {𝒱}^{*}=\sigma +\delta +\mu , \end{array}$$ (36)

the (3,3) entry simplifies to 0. Thus, the Jacobian becomes:


$$\begin{array}{c} J({ℰ}^{*})=[\begin{array}{cccc} -\beta {ℐ}^{*}-\kappa -\mu & \omega & -\beta {𝒮}^{*} & 0\\ \kappa & -\xi \beta {ℐ}^{*}-(\omega +\mu ) & -\xi \beta {𝒱}^{*} & 0\\ \beta {ℐ}^{*} & \xi \beta {ℐ}^{*} & 0 & 0\\ 0 & 0 & \delta & -\mu \end{array}]. \end{array}$$ (37)

The characteristic equation of the endemic equilibrium takes the form:


$$\begin{array}{c} \lambda^{4}+a_{1}\lambda^{3}+a_{2}\lambda^{2}+a_{3}\lambda +a_{4}=0, \end{array}$$ (38)

where one eigenvalue is λ=−μ and the remaining three eigenvalues satisfy the cubic equation:


$$\begin{array}{c} \lambda^{3}+b_{1}\lambda^{2}+b_{2}\lambda +b_{3}=0. \end{array}$$ (39)

The coefficients are given by:


$$\begin{array}{cc} b_{1} & =\beta {ℐ}^{*}+\kappa +2\mu +\xi \beta {ℐ}^{*}+\omega , \end{array}$$ (40)

$$\begin{array}{cc} b_{2} & =\mu (\beta {ℐ}^{*}+\xi \beta {ℐ}^{*}+\kappa +\mu +\omega )+\beta^{2}{ℐ}^{*}{𝒮}^{*}+\xi^{2}\beta^{2}{ℐ}^{*}{𝒱}^{*}+\xi \beta^{2}{ℐ}^{*}{𝒮}^{*} \end{array}$$ (41)

$$\begin{array}{cc} & \;+\xi \beta^{2}{ℐ}^{*}{𝒱}^{*}+\kappa \xi \beta {ℐ}^{*}+\beta {ℐ}^{*}\omega , \end{array}$$ (42)

$$\begin{array}{cc} b_{3} & =\beta^{2}{ℐ}^{*}{𝒮}^{*}\mu +\xi^{2}\beta^{2}{ℐ}^{*}{𝒱}^{*}\mu +\xi \beta^{2}{ℐ}^{*}{𝒮}^{*}\mu +\xi \beta^{2}{ℐ}^{*}{𝒱}^{*}\mu +\kappa \xi \beta {ℐ}^{*}\mu +\beta {ℐ}^{*}\omega \mu \end{array}$$ (43)

$$\begin{array}{cc} & \;+\beta^{2}{ℐ}^{*}{𝒮}^{*}(\kappa +\omega )+\xi^{2}\beta^{2}{ℐ}^{*}{𝒱}^{*}(\kappa +\omega )+\xi \beta^{2}{ℐ}^{*}{𝒮}^{*}(\kappa +\omega ) \end{array}$$ (44)

$$\begin{array}{cc} & \;+\xi \beta^{2}{ℐ}^{*}{𝒱}^{*}(\kappa +\omega )+\beta^{2}{ℐ}^{*}{𝒮}^{*}\xi \beta {ℐ}^{*}+\xi^{2}\beta^{2}{ℐ}^{*}{𝒱}^{*}\xi \beta {ℐ}^{*}. \end{array}$$ (45)

Clearly, when *b*_1_ > 0, *b*_2_ > 0, *b*_3_ > 0, and if the condition of the Routh-Hurwitz criterion, namely $b_{1}b_{2}>b_{3}$, is satisfied, then it can be inferred that all the roots of the characteristic equation of $J({ℰ}^{*})$ will have either negative real parts or will be negative. Thus, when *b*_1_ > 0, *b*_2_ > 0, *b*_3_ > 0, and $b_{1}b_{2}>b_{3}$, the endemic equilibrium ${ℰ}^{*}$ is locally asymptotically stable for all α∈(0,1][50].

The disease-free equilibrium of the system is globally asymptotically stable [9,11]. For $R_{v}>1$, the unique endemic equilibrium ${ℰ}^{*}=({𝒮}^{*},{𝒱}^{*},{ℐ}^{*},{ℛ}^{*})$ of model (9) is locally asymptotically stable in the interior of Ψ [9].

**Fig 2:**
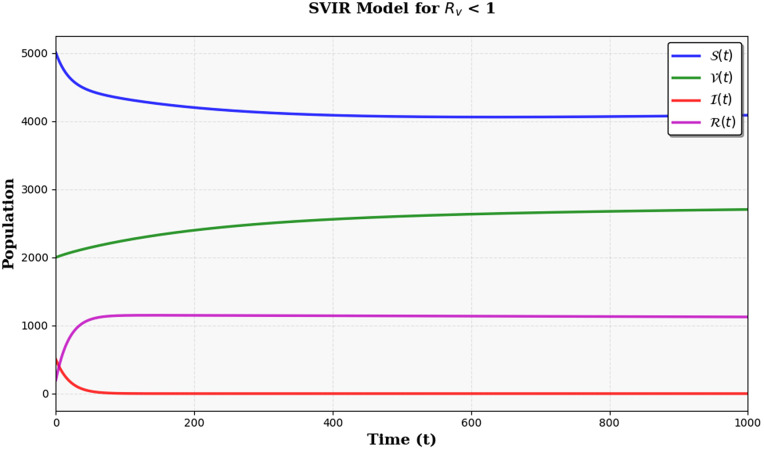
Stability of the populations 𝒮(t), 𝒱(t), ℐ(t), and ℛ(t) for $R_{v}=0.9784<1$.

**Fig 3:**
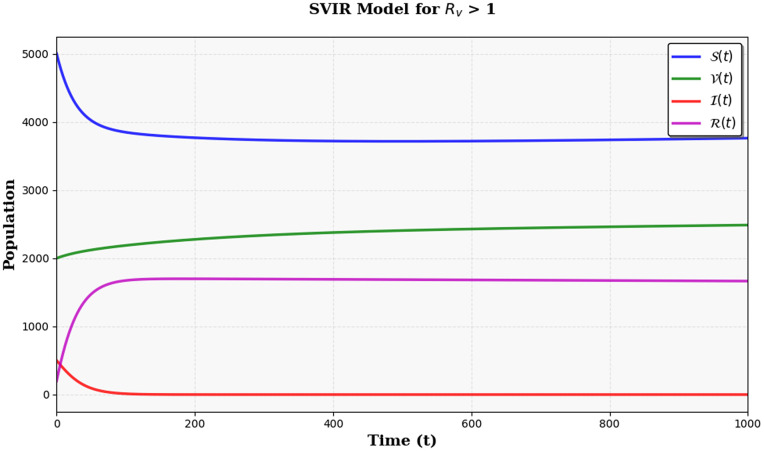
Stability of the populations 𝒮(t), 𝒱(t), ℐ(t), and ℛ(t) for $R_{v}=1.4676>1$.

Figs 2 and 3 show the behavior of the SVIR model for two different values of the transmission rate β. When β=0.00001, the reproduction number is $R_{v}=0.9784<1$, whereas for β=0.000015, the reproduction number becomes $R_{v}=1.4676>1$. All the other parameter values are taken from Table 1.

### 4.4 Sensitivity analysis

The normalized forward sensitivity index of $R_{v}$ with respect to a parameter *q* is defined as [51,52]:


$$\begin{array}{c} \Upsilon_{q}^{R_{v}}=\frac{q}{R_{v}}\cdot \frac{\partial R_{v}}{\partial q}. \end{array}$$

Using $R_{v}=R_{0}\cdot \frac{\mu (1-\pi +\xi \pi )+\omega +\xi \kappa }{\kappa +\mu +\omega }$ with $R_{0}=\frac{\beta \lambda }{\mu (\sigma +\delta +\mu )}$, the sensitivity indices for all parameters are:


$$\begin{array}{c} \Upsilon_{\beta }^{R_{v}}=\frac{\beta }{R_{v}}\frac{\partial R_{v}}{\partial \beta }=1, \end{array}$$

$$\begin{array}{c} \Upsilon_{\lambda }^{R_{v}}=\frac{\lambda }{R_{v}}\frac{\partial R_{v}}{\partial \lambda }=1, \end{array}$$

$$\begin{array}{c} \Upsilon_{\pi }^{R_{v}}=\frac{\pi }{R_{v}}\frac{\partial R_{v}}{\partial \pi }=\frac{\mu \pi (\xi -1)}{\mu (1-\pi +\xi \pi )+\omega +\xi \kappa }=-1.702\times 10^{-5}, \end{array}$$

$$\begin{array}{c} \Upsilon_{\kappa }^{R_{v}}=\frac{\kappa }{R_{v}}\frac{\partial R_{v}}{\partial \kappa }=-\frac{\kappa [\mu (\pi -1)+\omega ](\xi -1)}{\left(\kappa +\mu +\omega )[\mu (1-\pi +\xi \pi )+\omega +\xi \kappa \right]}=-0.4013, \end{array}$$

$$\begin{array}{c} \Upsilon_{\omega }^{R_{v}}=\frac{\omega }{R_{v}}\frac{\partial R_{v}}{\partial \omega }=-\frac{\omega (\kappa +\mu \pi )(\xi -1)}{\left(\kappa +\mu +\omega )[\mu (1-\pi +\xi \pi )+\omega +\xi \kappa \right]}=0.3976, \end{array}$$

$$\begin{array}{c} \Upsilon_{\xi }^{R_{v}}=\frac{\xi }{R_{v}}\frac{\partial R_{v}}{\partial \xi }=\frac{\xi (\kappa +\mu \pi )}{\mu (1-\pi +\xi \pi )+\omega +\xi \kappa }=6.716\times 10^{-4}, \end{array}$$

$$\begin{array}{c} \Upsilon_{\sigma }^{R_{v}}=\frac{\sigma }{R_{v}}\frac{\partial R_{v}}{\partial \sigma }=-\frac{\sigma }{\mu +\delta +\sigma }=-4.508\times 10^{-4}, \end{array}$$

$$\begin{array}{c} \Upsilon_{\delta }^{R_{v}}=\frac{\delta }{R_{v}}\frac{\partial R_{v}}{\partial \delta }=-\frac{\delta }{\mu +\delta +\sigma }=-0.9993. \end{array}$$

$$\begin{array}{c} \Upsilon_{\mu }^{R_{v}}=-1-\frac{\mu }{\kappa +\mu +\omega }+\frac{\mu (1-\pi +\xi \pi )}{\mu (1-\pi +\xi \pi )+\omega +\xi \kappa }-\frac{\mu }{\mu +\delta +\sigma }=-0.9966. \end{array}$$

The normalized sensitivity indices show that the transmission rate and recruitment rate satisfy $\Upsilon_{\beta }^{R_{v}}=1$ and $\Upsilon_{\lambda }^{R_{v}}=1$, indicating that these parameters strongly enhance disease transmission and contribute directly to increasing the effective reproduction number. The disease-induced death rate and natural death rate satisfy $\Upsilon_{\delta }^{R_{v}}=-0.9993$ and $\Upsilon_{\mu }^{R_{v}}=-0.9966$, respectively, showing strong negative effects on $R_{v}$ by reducing the infectious population; however, increasing natural mortality is not a feasible public health intervention. The vaccination rate satisfies $\Upsilon_{\kappa }^{R_{v}}=-0.4013$, confirming that increasing vaccination significantly suppresses disease spread and acts as an important control parameter in the model. In contrast, the waning immunity rate satisfies $\Upsilon_{\omega }^{R_{v}}=0.3976$, implying that loss of immunity contributes positively to disease transmission by increasing the susceptible population. Furthermore, the proportion of vaccinated newborns, vaccine efficacy, and progression rate satisfy $\Upsilon_{\pi }^{R_{v}}=-1.702\times 10^{-5}$, $\Upsilon_{\xi }^{R_{v}}=6.716\times 10^{-4}$, and $\Upsilon_{\sigma }^{R_{v}}=-4.508\times 10^{-4}$, respectively, indicating that these parameters have only negligible influence on the effective reproduction number. Overall, the parameters β and λ promote disease transmission, whereas κ, δ, and μ reduce the spread of infection, with κ representing one of the most meaningful practical control strategies in the model.

**Fig 4:**
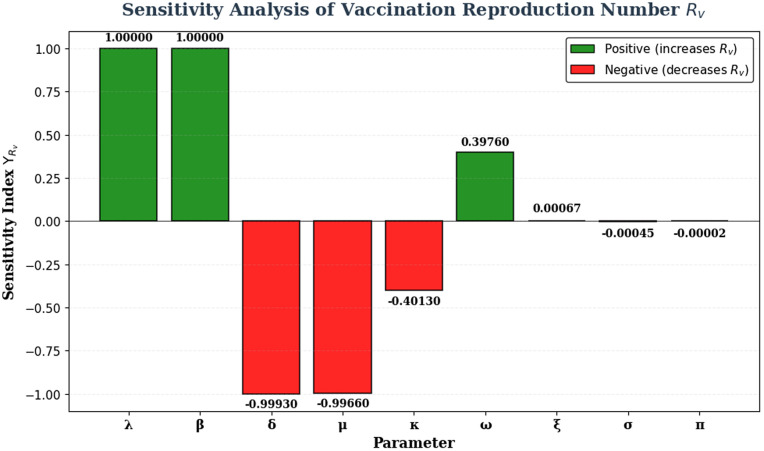
Sensitivity analysis of the reproduction number $R_{v}$ with respect to the model parameters.

Fig 4 illustrates the normalized sensitivity indices of the vaccination reproduction number $R_{v}$ with respect to the model parameters. The figure shows that β and λ have the strongest positive influence on disease transmission, while δ, μ, and κ contribute significantly to reducing the spread of the disease.

### 4.5 Positivity of solutions for the fractional-order system

**Theorem 1.** *Let* 𝒮(0),𝒱(0),ℐ(0),ℛ(0)≥0 *and* $\alpha_{𝒮},\alpha_{𝒱},\alpha_{ℐ},\alpha_{ℛ}\in (0,1]$*. Then the solution* (𝒮(t),𝒱(t),ℐ(t),ℛ(t)) *of system (9) remains nonnegative for all* t≥0*.*

*Proof.* The Caputo fractional derivative satisfies the generalized Mean Value Theorem [53]: if ${\;}^{C}D_{t}^{\alpha }f(t)\geq 0$ for t∈[0,T], then f(t)≥f(0). Assume there exists a first time $t^{*}>0$ such that one variable becomes zero while others remain nonnegative.

**Case 1:** $𝒮(t^{*})=0$. From (9):


$$\begin{array}{c} {\;}^{C}D_{t}^{\alpha_{𝒮}}𝒮(t^{*})=(1-\pi )\lambda +\omega 𝒱(t^{*})\geq 0. \end{array}$$

Thus 𝒮(t)≥𝒮(0)≥0 for all *t*.

**Case 2:** $𝒱(t^{*})=0$. Then:


$$\begin{array}{c} {\;}^{C}D_{t}^{\alpha_{𝒱}}𝒱(t^{*})=\pi \lambda +\kappa 𝒮(t^{*})\geq 0. \end{array}$$

**Case 3:** $ℐ(t^{*})=0$. Then:


$$\begin{array}{c} {\;}^{C}D_{t}^{\alpha_{ℐ}}ℐ(t^{*})=0. \end{array}$$

**Case 4:** $ℛ(t^{*})=0$. Then:


$$\begin{array}{c} {\;}^{C}D_{t}^{\alpha_{ℛ}}ℛ(t^{*})=\delta ℐ(t^{*})\geq 0. \end{array}$$

In each case, the Caputo derivative at the zero-crossing point is nonnegative. Therefore, by the generalized Mean Value Theorem, no state variable can become negative. Hence, following the arguments in the previous literature [43,52], we obtain


$$\begin{array}{c} 𝒮(t),𝒱(t),ℐ(t),ℛ(t)\geq 0,\;\forall t\geq 0. \end{array}$$

Therefore, the region ${\mathbb{R}}_{+}^{4}$ is positively invariant.

## 5 Laplace Adomian decomposition method

We consider a general nonlinear fractional ordinary differential equation of the form


$$\begin{array}{c} D_{t}^{\alpha }𝒮(t)=L(𝒮(t))+N(𝒮(t))+g(t),\;t\geq 0, \end{array}$$ (46)

subject to the initial condition


$$\begin{array}{c} 𝒮(0)=f(t_{0}) \end{array}$$ (47)

where ${\;}^{C}D_{t}^{\alpha }$ denotes the Caputo fractional derivative of order 0<α≤1, *L* is a linear differential operator, *N* is a nonlinear differential operator, and *g*(*t*) is a known source function.

Applying the Laplace transform to Eq. (46) and using the Laplace property of the Caputo fractional derivative, we obtain


$$\begin{array}{c} s^{\alpha }S(s)-s^{\alpha -1}f(t_{0})=ℒL(𝒮)+ℒN(𝒮)+ℒg(t). \end{array}$$ (48)

$$\begin{array}{c} S(s)=\frac{f(t_{0})}{s}+\frac{1}{s^{\alpha }}ℒ\{g(t)\}+\frac{1}{s^{\alpha }}ℒ\;\left[L(𝒮)+N(𝒮)\right]. \end{array}$$ (49)

Applying the inverse Laplace transform to Eq. (49), we obtain


$$\begin{array}{c} 𝒮(t)=h(t)+{ℒ}^{-1}\;\left[\frac{1}{s^{\alpha }}ℒ\;\left(L(𝒮)+N(𝒮)\right)\right], \end{array}$$ (50)

where


$$\begin{array}{c} h(t)=f(t_{0})+{ℒ}^{-1}\;\left[\frac{1}{s^{\alpha }}ℒ\{g(t)\}\right]. \end{array}$$ (51)

According to the Adomian decomposition method, the solution 𝒮(t) is expressed as an infinite series


$$\begin{array}{c} 𝒮(t)=\sum_{n=0}^{\infty }{𝒮}_{n}(t), \end{array}$$ (52)

and the nonlinear operator is decomposed as


$$\begin{array}{c} N(𝒮)=\sum_{n=0}^{\infty }A_{n}, \end{array}$$ (53)

where $A_{n}$ are the Adomian polynomials defined by


$$\begin{array}{c} A_{n}=\frac{1}{n!}\frac{d^{n}}{d\lambda^{n}}{\left[N\;\left(\sum_{i=0}^{\infty }\lambda^{i}{𝒮}_{i}\right)\right]}_{\lambda =0},\;n=0,1,2,\ldots \end{array}$$ (54)

Substituting equations (52)–(53) into Eq. (50), we obtain:


$$\begin{array}{c} \sum_{n=0}^{\infty }{𝒮}_{n}(t)=h(t)+{ℒ}^{-1}\;\left[\frac{1}{s^{\alpha }}ℒ\;\left(L\;\left(\sum_{n=0}^{\infty }{𝒮}_{n}(t)\right)+\sum_{n=0}^{\infty }A_{n}(t)\right)\right]. \end{array}$$ (55)

Hence, the iterative scheme of the LADM is obtained as


$$\begin{array}{c} {𝒮}_{0}(t)=h(t), \end{array}$$ (56)

$$\begin{array}{c} {𝒮}_{1}(t)={ℒ}^{-1}\left[\frac{1}{s^{\alpha }}ℒ(L({𝒮}_{0}(t))+A_{0})\right], \end{array}$$ (57)

$$\begin{array}{c} {𝒮}_{2}(t)={ℒ}^{-1}\left[\frac{1}{s^{\alpha }}ℒ(L({𝒮}_{1}(t))+A_{1})\right], \end{array}$$ (58)

$$\begin{array}{c} \vdots \end{array}$$ (59)

$$\begin{array}{c} {𝒮}_{n+1}(t)={ℒ}^{-1}\left[\frac{1}{s^{\alpha }}ℒ(L({𝒮}_{n}(t))+A_{n})\right],\;n\geq 0. \end{array}$$ (60)

Therefore, an approximate solution of order *k* is given by


$$\begin{array}{c} 𝒮(t)\approx \sum_{n=0}^{k}{𝒮}_{n}(t). \end{array}$$ (61)

## 6 Stability, convergence and error analysis

The convergence and stability analysis of the proposed LADM are presented in this section. The analysis establishes the existence, uniqueness, convergence behavior, and error estimates of the iterative solution sequence generated by the method. These theoretical results ensure the reliability and stability of the proposed numerical scheme for solving the fractional SVIR model.

**Theorem 62.** *Consider a Banach space* (Ξ,‖·‖) *and let* 𝒯:Ξ→Ξ *be the self-map of* Ξ*, Then, the iterative scheme associated with the Laplace Adomian Decomposition Method defined by:*


$$\begin{array}{c} 𝒯({𝒮}_{m}(t))={𝒮}_{m+1}(t)=f(t_{0})+{ℒ}^{-1}\left[\frac{1}{s^{\alpha }}ℒ[g(t)+L{𝒮}_{m}(t)+A_{m}(t)]\right], \end{array}$$ (62)

*is Picard* 𝒯*-stable if there exist constants* $\epsilon_{0},\epsilon_{1}\geq 0$ *and* ϵ<1 *such that:*

1. $\left\|L{𝒮}_{m}(t)-L{𝒮}_{n}(t)\|\leq \epsilon_{0}\|{𝒮}_{m}(t)-{𝒮}_{n}(t)\right\|$,
2. $\left\|A_{m}(𝒮)-A_{n}(𝒮)\|\leq \epsilon_{1}\|{𝒮}_{m}(t)-{𝒮}_{n}(t)\right\|$,
3. $\epsilon =\epsilon_{0}+\epsilon_{1}\left\|\frac{t^{\alpha }}{(\alpha )!}\right\|<1$.

*Proof.* For m,n∈ℕ, consider two successive iterates:


$$\begin{array}{c} 𝒯({𝒮}_{m}(t))=f(t_{0})+{ℒ}^{-1}\left[\frac{1}{s^{\alpha }}ℒ[g(t)+L{𝒮}_{m}(t)+A_{m}(𝒮)]\right], \end{array}$$ (63)

$$\begin{array}{c} 𝒯({𝒮}_{n}(t))=f(t_{0})+{ℒ}^{-1}\left[\frac{1}{s^{\alpha }}ℒ[g(t)+L{𝒮}_{n}(t)+A_{n}(𝒮)]\right]. \end{array}$$ (64)

Subtracting (64) from (63) yields:


$$\begin{array}{c} 𝒯({𝒮}_{m}(t))-𝒯({𝒮}_{n}(t))=-{ℒ}^{-1}\left[\frac{1}{s^{\alpha }}ℒ[L({𝒮}_{m}(t)-{𝒮}_{n}(t))+(A_{m}(𝒮)-A_{n}(𝒮))]\right]. \end{array}$$ (65)

Taking norms and using the linearity of the Laplace transform:


$$\begin{array}{cc} \left\|𝒯({𝒮}_{m}(t))-𝒯({𝒮}_{n}(t))\right\| & \leq \left\|{ℒ}^{-1}\left[\frac{1}{s^{\alpha }}ℒ[L({𝒮}_{m}(t)-{𝒮}_{n}(t))]\right]\right\|+\left\|{ℒ}^{-1}\left[\frac{1}{s^{\alpha }}ℒ[A_{m}(𝒮)-A_{n}(𝒮)]\right]\right\|. \end{array}$$ (66)

Using the given conditions and the Laplace transform property, we obtain:


$$\begin{array}{cc} \left\|𝒯({𝒮}_{m}(t))-𝒯({𝒮}_{n}(t))\right\| & \leq \epsilon_{0}\|{𝒮}_{m}(t)-{𝒮}_{n}(t)\|\left\|{ℒ}^{-1}\left[\frac{1}{s^{\alpha }}\right]\right\|+\epsilon_{1}\|{𝒮}_{m}(t)-{𝒮}_{n}(t)\|\left\|{ℒ}^{-1}\left[\frac{1}{s^{\alpha }}\right]\right\|\\ & =(\epsilon_{0}+\epsilon_{1})\|{𝒮}_{m}(t)-{𝒮}_{n}(t)\|\left\|\frac{t^{\alpha }}{(\alpha )!}\right\|\\ & =\epsilon \|{𝒮}_{m}(t)-{𝒮}_{n}(t)\|. \end{array}$$

where $\epsilon =\epsilon_{0}+\epsilon_{1}\left\|\frac{t^{\alpha }}{(\alpha )!}\right\|$.

Thus, the self-map 𝒯 possesses a fixed point. Now,


$$\begin{array}{c} \left\|𝒯({𝒮}_{n}(t))-𝒯({𝒮}_{m}(t))\|\leq \gamma \|{𝒮}_{n}(t)-{𝒮}_{m}(t)\|+\epsilon \|{𝒮}_{n}(t)-{𝒮}_{m}(t)\right\| \end{array}$$ (67)

For γ=0, if


$$\begin{array}{c} \epsilon =\epsilon_{0}+\epsilon_{1}\left\|\frac{t^{\alpha }}{(\alpha )!}\right\|<1, \end{array}$$

then, by the Picard-𝒯 stability criterion [54], the Laplace–Adomian Decomposition Method is Picard-𝒯 stable.

**Theorem 3.** *Let* (Ξ,‖·‖) *be a Banach space and let* 𝒯:Ξ→Ξ *be the LADM iteration mapping. Then, for any initial guess* ${𝒮}_{0}\in \Xi$*, the sequence* $\left\{{𝒮}_{n}\right\}$ *generated by the iterative scheme converges to a unique fixed point* ${𝒮}^{*}(t)$*, provided* 0<ϵ<1*,* $\|{𝒮}_{0}\|<\infty$*, and* $\left\|{𝒮}_{n+1}\|\leq \epsilon \|{𝒮}_{n}\right\|$*.*

*Proof.* Define the sequence of partial sums:


$$\begin{array}{cc} \Psi_{0} & ={𝒮}_{0},\\ \Psi_{1} & ={𝒮}_{0}+{𝒮}_{1},\\ \Psi_{2} & ={𝒮}_{0}+{𝒮}_{1}+{𝒮}_{2},\\ & \vdots \\ \Psi_{n} & =\sum_{k=0}^{n}{𝒮}_{k}. \end{array}$$ (68)

Now consider the difference between successive partial sums:


$$\begin{array}{c} \|\Psi_{n+1}-\Psi_{n}\|=\|{𝒮}_{n+1}\|\leq \epsilon \|{𝒮}_{n}\|\leq \epsilon^{n+1}\|{𝒮}_{0}\|. \end{array}$$ (69)

To show that $\left\{\Psi_{n}\right\}$ is a Cauchy sequence, take m>n∈ℕ and apply the triangle inequality:


$$\begin{array}{cc} \left\|\Psi_{m}-\Psi_{n}\right\| & \leq \|\Psi_{n}-\Psi_{n+1}\|+\|\Psi_{n+1}-\Psi_{n+2}\|+\cdots +\|\Psi_{m-1}-\Psi_{m}\|\\ & \leq \epsilon^{n+1}\|{𝒮}_{0}\|+\epsilon^{n+2}\|{𝒮}_{0}\|+\cdots +\epsilon^{m}\|{𝒮}_{0}\|\\ & =\epsilon^{n+1}\|{𝒮}_{0}\|(1+\epsilon +\epsilon^{2}+\cdots +\epsilon^{m-n-1})\\ & =\epsilon^{n+1}\|{𝒮}_{0}\|\left(\frac{1-\epsilon^{m-n}}{1-\epsilon }\right)\\ & \leq \frac{\epsilon^{n+1}}{1-\epsilon }\|{𝒮}_{0}\|. \end{array}$$ (70)

As n→∞, $\epsilon^{n+1}\to 0$, so $\left\{\Psi_{n}\right\}$ is Cauchy and converges to ${𝒮}^{*}(t)$.

To show uniqueness, assume that ${𝒮}^{*}$ and $\tilde{𝒮}$ are two fixed points of 𝒯. Then


$$\begin{array}{c} \left\|{𝒮}^{*}-\tilde{𝒮}\|=\|𝒯({𝒮}^{*})-𝒯(\tilde{𝒮})\right\|\\ \leq \epsilon \|{𝒮}^{*}-\tilde{𝒮}\|. \end{array}$$ (71)

Thus,


$$\begin{array}{c} (1-\epsilon )\|{𝒮}^{*}-\tilde{𝒮}\|\leq 0. \end{array}$$

Since 1−ϵ>0, it follows that


$$\begin{array}{c} {𝒮}^{*}=\tilde{𝒮}. \end{array}$$

Therefore, ${𝒮}^{*}$ is the only fixed point of 𝒯. Hence, the LADM iteration converges to a unique fixed point ${𝒮}^{*}(t)$.

**Theorem 4.** *Let* 𝒮(t) *be the exact solution and let* $\Psi_{n}(t)=\sum_{k=0}^{n}{𝒮}_{k}(t)$ *be the nth LADM approximation. If* $\left\|{𝒮}_{k+1}(t)\|\leq \epsilon \|{𝒮}_{k}(t)\right\|$ *for some* 0<ϵ<1 *and all* k≥0*, then the maximum absolute error satisfies:*


$$\begin{array}{c} \|𝒮(t)-\Psi_{n}(t)\|<\frac{\epsilon^{n+1}}{1-\epsilon }\|{𝒮}_{0}(t)\|,\;\text{for all }t\in [0,T]. \end{array}$$ (72)

*Proof.* From the convergence proof, we have:


$$\begin{array}{cc} \left\|𝒮(t)-\Psi_{n}(t)\right\| & =\left\|\sum_{k=n+1}^{\infty }{𝒮}_{k}(t)\right\|\leq \sum_{k=n+1}^{\infty }\|{𝒮}_{k}(t)\|\leq \sum_{k=n+1}^{\infty }\epsilon^{k}\|{𝒮}_{0}(t)\|\\ & =\epsilon^{n+1}\|{𝒮}_{0}(t)\|\sum_{k=0}^{\infty }\epsilon^{k}=\frac{\epsilon^{n+1}}{1-\epsilon }\|{𝒮}_{0}(t)\|. \end{array}$$

## 7 Numerical solution

From (9), the fractional-order SVIR epidemic model for rotavirus is given by


$$\begin{array}{cc} D_{t}^{\alpha_{𝒮}}𝒮(t) & =(1-\pi )\lambda -\beta 𝒮(t)ℐ(t)-\kappa 𝒮(t)+\omega 𝒱(t)-\mu 𝒮(t),\\ D_{t}^{\alpha_{𝒱}}𝒱(t) & =\pi \lambda +\kappa 𝒮(t)-\xi \beta 𝒱(t)ℐ(t)-(\omega +\mu )𝒱(t),\\ D_{t}^{\alpha_{ℐ}}ℐ(t) & =\beta 𝒮(t)ℐ(t)+\xi \beta 𝒱(t)ℐ(t)-(\sigma +\delta +\mu )ℐ(t),\\ D_{t}^{\alpha_{ℛ}}ℛ(t) & =\delta ℐ(t)-\mu ℛ(t). \end{array}$$ (73)

We consider the following initial conditions [10,17]:


$$\begin{array}{c} 𝒮(0)=500,\;𝒱(0)=350,\;ℐ(0)=150,\;ℛ(0)=50. \end{array}$$ (74)

Applying Laplace transform to each equation in (73):


$$\begin{array}{cc} s^{\alpha_{𝒮}}S(s)-s^{\alpha_{𝒮}-1}𝒮(0) & =ℒ[(1-\pi )\lambda -\beta 𝒮ℐ-\kappa 𝒮+\omega 𝒱-\mu 𝒮],\\ s^{\alpha_{𝒱}}V(s)-s^{\alpha_{𝒱}-1}𝒱(0) & =ℒ[\pi \lambda +\kappa 𝒮-\xi \beta 𝒱ℐ-(\omega +\mu )𝒱],\\ s^{\alpha_{ℐ}}I(s)-s^{\alpha_{ℐ}-1}ℐ(0) & =ℒ[\beta 𝒮ℐ+\xi \beta 𝒱ℐ-(\sigma +\delta +\mu )ℐ],\\ s^{\alpha_{ℛ}}R(s)-s^{\alpha_{ℛ}-1}ℛ(0) & =ℒ[\delta ℐ-\mu ℛ]. \end{array}$$ (75)

Using the initial conditions:


$$\begin{array}{cc} S(s) & =\frac{500}{s}+\frac{1}{s^{\alpha_{𝒮}}}ℒ[(1-\pi )\lambda ]-\frac{1}{s^{\alpha_{𝒮}}}ℒ[\beta 𝒮ℐ+\kappa 𝒮+\mu 𝒮-\omega 𝒱]\\ V(s) & =\frac{350}{s}+\frac{1}{s^{\alpha_{𝒱}}}ℒ[\pi \lambda ]+\frac{1}{s^{\alpha_{𝒱}}}ℒ[\kappa 𝒮-\xi \beta 𝒱ℐ-(\omega +\mu )𝒱],\\ I(s) & =\frac{150}{s}+\frac{1}{s^{\alpha_{ℐ}}}ℒ[\beta 𝒮ℐ+\xi \beta 𝒱ℐ-(\sigma +\delta +\mu )ℐ],\\ R(s) & =\frac{50}{s}+\frac{1}{s^{\alpha_{ℛ}}}ℒ[\delta ℐ-\mu ℛ]. \end{array}$$ (76)

Applying inverse Laplace transform to each equation:


$$\begin{array}{cc} 𝒮(t) & =500+{ℒ}^{-1}\left[\frac{1}{s^{\alpha_{𝒮}}}ℒ[(1-\pi )\lambda ]\right]-{ℒ}^{-1}\left[\frac{1}{s^{\alpha_{𝒮}}}ℒ[\beta 𝒮ℐ+\kappa 𝒮+\mu 𝒮-\omega 𝒱]\right],\\ 𝒱(t) & =350+{ℒ}^{-1}\left[\frac{1}{s^{\alpha_{𝒱}}}ℒ[\pi \lambda ]\right]+{ℒ}^{-1}\left[\frac{1}{s^{\alpha_{𝒱}}}ℒ[\kappa 𝒮-\xi \beta 𝒱ℐ-(\omega +\mu )𝒱]\right],\\ ℐ(t) & =150+{ℒ}^{-1}\left[\frac{1}{s^{\alpha_{ℐ}}}ℒ[\beta 𝒮ℐ+\xi \beta 𝒱ℐ-(\sigma +\delta +\mu )ℐ]\right],\\ ℛ(t) & =50+{ℒ}^{-1}\left[\frac{1}{s^{\alpha_{ℛ}}}ℒ[\delta ℐ-\mu ℛ]\right]. \end{array}$$ (77)

For the numerical results, we used the parameter values listed in Table 1


$$\begin{array}{cc} 𝒮(t) & =500+{ℒ}^{-1}\left[\frac{1}{s^{\alpha_{𝒮}}}ℒ[0.4109(1-0.001884)]\right]-{ℒ}^{-1}\left[\frac{1}{s^{\alpha_{𝒮}}}ℒ[0.0001𝒮ℐ+0.001884𝒮+0.00002537𝒮-0.002778𝒱]\right],\\ 𝒱(t) & =350+{ℒ}^{-1}\left[\frac{1}{s^{\alpha_{𝒱}}}ℒ[0.4109\times 0.001884]\right]+{ℒ}^{-1}\left[\frac{1}{s^{\alpha_{𝒱}}}ℒ[0.001884𝒮-0.001\times 0.0001𝒱ℐ-0.00280337𝒱]\right],\\ ℐ(t) & =150+{ℒ}^{-1}\left[\frac{1}{s^{\alpha_{ℐ}}}ℒ[0.0001𝒮ℐ+0.001\times 0.0001𝒱ℐ-(0.00004466+0.099+0.00002537)ℐ]\right],\\ ℛ(t) & =50+{ℒ}^{-1}\left[\frac{1}{s^{\alpha_{ℛ}}}ℒ[0.099ℐ-0.00002537ℛ]\right]. \end{array}$$ (78)

Hence we get,


$$\begin{array}{cc} 𝒮(t) & =500+{ℒ}^{-1}\left[\frac{1}{s^{\alpha_{𝒮}}}ℒ[0.410126]\right]-{ℒ}^{-1}\left[\frac{1}{s^{\alpha_{𝒮}}}ℒ[0.0001𝒮ℐ+0.0019094𝒮-0.002778𝒱]\right],\\ 𝒱(t) & =350+{ℒ}^{-1}\left[\frac{1}{s^{\alpha_{𝒱}}}ℒ[0.00077414]\right]+{ℒ}^{-1}\left[\frac{1}{s^{\alpha_{𝒱}}}ℒ[0.001884𝒮-0.0000001𝒱ℐ-0.002803𝒱]\right],\\ ℐ(t) & =150+{ℒ}^{-1}\left[\frac{1}{s^{\alpha_{ℐ}}}ℒ[0.0001𝒮ℐ+0.0000001𝒱ℐ-0.09907ℐ]\right],\\ ℛ(t) & =50+{ℒ}^{-1}\left[\frac{1}{s^{\alpha_{ℛ}}}ℒ[0.099ℐ-0.00002537ℛ]\right]. \end{array}$$ (79)

According to the Adomian Decomposition Method, the solution is expressed as:


$$\begin{array}{c} 𝒮(t)=\sum_{n=0}^{\infty }{𝒮}_{n}(t),\;𝒱(t)=\sum_{n=0}^{\infty }{𝒱}_{n}(t),\;ℐ(t)=\sum_{n=0}^{\infty }{ℐ}_{n}(t),\;ℛ(t)=\sum_{n=0}^{\infty }{ℛ}_{n}(t). \end{array}$$ (80)

The nonlinear terms are decomposed as:


$$\begin{array}{c} 𝒮(t)ℐ(t)=\sum_{n=0}^{\infty }A_{n}(t),\;𝒱(t)ℐ(t)=\sum_{n=0}^{\infty }B_{n}(t). \end{array}$$ (81)

Using (54) the Adomian polynomials are:


$$\begin{array}{c} A_{n}=\sum_{k=0}^{n}{𝒮}_{k}{ℐ}_{n-k},\;B_{n}=\sum_{k=0}^{n}{𝒱}_{k}{ℐ}_{n-k},\;n=0,1,2,\ldots \end{array}$$ (82)

The first few polynomials are:


$$\begin{array}{cccc} A_{0} & ={𝒮}_{0}{ℐ}_{0}, & B_{0} & ={𝒱}_{0}{ℐ}_{0},\\ A_{1} & ={𝒮}_{0}{ℐ}_{1}+{𝒮}_{1}{ℐ}_{0}, & B_{1} & ={𝒱}_{0}{ℐ}_{1}+{𝒱}_{1}{ℐ}_{0},\\ A_{2} & ={𝒮}_{0}{ℐ}_{2}+{𝒮}_{1}{ℐ}_{1}+{𝒮}_{2}{ℐ}_{0}, & B_{2} & ={𝒱}_{0}{ℐ}_{2}+{𝒱}_{1}{ℐ}_{1}+{𝒱}_{2}{ℐ}_{0},\\ A_{3} & ={𝒮}_{0}{ℐ}_{3}+{𝒮}_{1}{ℐ}_{2}+{𝒮}_{2}{ℐ}_{1}+{𝒮}_{3}{ℐ}_{0}, & B_{3} & ={𝒱}_{0}{ℐ}_{3}+{𝒱}_{1}{ℐ}_{2}+{𝒱}_{2}{ℐ}_{1}+{𝒱}_{3}{ℐ}_{0}. \end{array}$$ (83)

Then the system in series form is:


$$\begin{array}{cc} \sum_{n=0}^{\infty }{𝒮}_{n}(t) & =500+{ℒ}^{-1}\left[\frac{1}{s^{\alpha_{𝒮}}}ℒ[0.410126]\right]-{ℒ}^{-1}\left[\frac{1}{s^{\alpha_{𝒮}}}ℒ[0.0001\sum_{n=0}^{\infty }A_{n}+0.0019094\sum_{n=0}^{\infty }{𝒮}_{n}-0.002778\sum_{n=0}^{\infty }{𝒱}_{n}]\right],\\ \sum_{n=0}^{\infty }{𝒱}_{n}(t) & =350+{ℒ}^{-1}\left[\frac{1}{s^{\alpha_{𝒱}}}ℒ[0.00077414]\right]+{ℒ}^{-1}\left[\frac{1}{s^{\alpha_{𝒱}}}ℒ[0.001884\sum_{n=0}^{\infty }{𝒮}_{n}-0.0000001\sum_{n=0}^{\infty }B_{n}-0.002803\sum_{n=0}^{\infty }{𝒱}_{n}]\right],\\ \sum_{n=0}^{\infty }{ℐ}_{n}(t) & =150+{ℒ}^{-1}\left[\frac{1}{s^{\alpha_{ℐ}}}ℒ[0.0001\sum_{n=0}^{\infty }A_{n}+0.0000001\sum_{n=0}^{\infty }B_{n}-0.09907\sum_{n=0}^{\infty }{ℐ}_{n}]\right],\\ \sum_{n=0}^{\infty }{ℛ}_{n}(t) & =50+{ℒ}^{-1}\left[\frac{1}{s^{\alpha_{ℛ}}}ℒ[0.099\sum_{n=0}^{\infty }{ℐ}_{n}-0.00002537\sum_{n=0}^{\infty }{ℛ}_{n}]\right]. \end{array}$$ (84)

Now, from equations (60) and (84) we get the initial iteration as follows:


$$\begin{array}{c} {𝒮}_{0}(t)=500+\frac{0.410126\;t^{\alpha_{𝒮}}}{(\alpha_{𝒮})!},\\ {𝒱}_{0}(t)=350+\frac{0.00077414\;t^{\alpha_{𝒱}}}{(\alpha_{𝒱})!},\\ {ℐ}_{0}(t)=150,\\ {ℛ}_{0}(t)=50. \end{array}$$

where (α)! denotes Γ(α+1). Now, from (84) and (60) the iterative scheme follows:


$$\begin{array}{c} \begin{array}{cc} {𝒮}_{n+1}(t) & =-{ℒ}^{-1}\;\left[\frac{1}{s^{\alpha_{𝒮}}}\;ℒ\;\left\{0.0001A_{n}+0.0019094{𝒮}_{n}-0.002778{𝒱}_{n}\right\}\right],\\ {𝒱}_{n+1}(t) & ={ℒ}^{-1}\;\left[\frac{1}{s^{\alpha_{𝒱}}}\;ℒ\;\left\{0.001884{𝒮}_{n}-0.0000001B_{n}-0.002803{𝒱}_{n}\right\}\right],\\ {ℐ}_{n+1}(t) & ={ℒ}^{-1}\;\left[\frac{1}{s^{\alpha_{ℐ}}}\;ℒ\;\left\{0.0001A_{n}+0.0000001B_{n}-0.09907{ℐ}_{n}\right\}\right],\\ {ℛ}_{n+1}(t) & ={ℒ}^{-1}\;\left[\frac{1}{s^{\alpha_{ℛ}}}\;ℒ\;\left\{0.099{ℐ}_{n}-0.00002537{ℛ}_{n}\right\}\right], \end{array}\;n\geq 0. \end{array}$$ (85)

Now, for the first iteration (*n* = 0), using $A_{0}={𝒮}_{0}{ℐ}_{0}$ and $B_{0}={𝒱}_{0}{ℐ}_{0}$, we obtain:


$$\begin{array}{cc} {𝒮}_{1}(t) & =-{ℒ}^{-1}\;[\frac{1}{s^{\alpha_{𝒮}}}ℒ[0.0001\;\left(150\;\left(500+\frac{0.410126\;t^{\alpha_{𝒮}}}{(\alpha_{𝒮})!}\right)\right)\\ & \;+\;0.0019094\;\left(500+\frac{0.410126\;t^{\alpha_{𝒮}}}{(\alpha_{𝒮})!}\right)-0.002778\;\left(350+\frac{0.00077414\;t^{\alpha_{𝒱}}}{(\alpha_{𝒱})!}\right)]],\\ {𝒱}_{1}(t) & ={ℒ}^{-1}\;[\frac{1}{s^{\alpha_{𝒱}}}ℒ[0.001884\;\left(500+\frac{0.410126\;t^{\alpha_{𝒮}}}{(\alpha_{𝒮})!}\right)\\ & \;-0.0000001\;\left(150\;\left(350+\frac{0.00077414\;t^{\alpha_{𝒱}}}{(\alpha_{𝒱})!}\right)\right)-0.002803\;\left(350+\frac{0.00077414\;t^{\alpha_{𝒱}}}{(\alpha_{𝒱})!}\right)]],\\ {ℐ}_{1}(t) & ={ℒ}^{-1}\;[\frac{1}{s^{\alpha_{ℐ}}}ℒ[0.0001\;\left(150\;\left(500+\frac{0.410126\;t^{\alpha_{𝒮}}}{(\alpha_{𝒮})!}\right)\right)\\ & \;+0.0000001\;\left(150\;\left(350+\frac{0.00077414\;t^{\alpha_{𝒱}}}{(\alpha_{𝒱})!}\right)\right)-0.09907(150)]],\\ {ℛ}_{1}(t) & ={ℒ}^{-1}\;[\frac{1}{s^{\alpha_{ℛ}}}ℒ[0.099(150)-0.00002537(50)]]. \end{array}$$

After simplifying the expressions inside the Laplace operators, we obtain


$$\begin{array}{cc} & {𝒮}_{1}(t)=-{ℒ}^{-1}\;\left[\frac{1}{s^{\alpha_{𝒮}}}ℒ\;\left[7.4824+0.00693498\;\frac{t^{\alpha_{𝒮}}}{(\alpha_{𝒮})!}-0.00000215056\;\frac{t^{\alpha_{𝒱}}}{(\alpha_{𝒱})!}\right]\right],\\ & {𝒱}_{1}(t)={ℒ}^{-1}\;\left[\frac{1}{s^{\alpha_{𝒱}}}ℒ\;\left[0.0443-0.000772677\;\frac{t^{\alpha_{𝒮}}}{(\alpha_{𝒮})!}-0.00000218153\;\frac{t^{\alpha_{𝒱}}}{(\alpha_{𝒱})!}\right]\right],\\ & {ℐ}_{1}(t)={ℒ}^{-1}\;\left[\frac{1}{s^{\alpha_{ℐ}}}ℒ\;\left[7.35525-0.00615189\;\frac{t^{\alpha_{𝒮}}}{(\alpha_{𝒮})!}-0.0000000116121\;\frac{t^{\alpha_{𝒱}}}{(\alpha_{𝒱})!}\right]\right],\\ & {ℛ}_{1}(t)={ℒ}^{-1}\;\left[\frac{1}{s^{\alpha_{ℛ}}}ℒ[14.8487]\right]. \end{array}$$

Hence,


$$\begin{array}{cc} & {𝒮}_{1}(t)=-\frac{7.4824\;t^{\alpha_{𝒮}}}{(\alpha_{𝒮})!}-\frac{0.00693498\;t^{2\alpha_{𝒮}}}{(2\alpha_{𝒮})!}+\frac{0.00000215056\;t^{\alpha_{𝒮}+\alpha_{𝒱}}}{(\alpha_{𝒮}+\alpha_{𝒱})!},\\ & {𝒱}_{1}(t)=\frac{0.0443\;t^{\alpha_{𝒱}}}{(\alpha_{𝒱})!}-\frac{0.000772677\;t^{\alpha_{𝒮}+\alpha_{𝒱}}}{(\alpha_{𝒮}+\alpha_{𝒱})!}+\frac{0.00000218153\;t^{2\alpha_{𝒱}}}{(2\alpha_{𝒱})!},\\ & {ℐ}_{1}(t)=-\frac{7.35525\;t^{\alpha_{ℐ}}}{(\alpha_{ℐ})!}+\frac{0.00615189\;t^{\alpha_{𝒮}+\alpha_{ℐ}}}{(\alpha_{𝒮}+\alpha_{ℐ})!}+\frac{0.0000000116121\;t^{\alpha_{𝒱}+\alpha_{ℐ}}}{(\alpha_{𝒱}+\alpha_{ℐ})!},\\ & {ℛ}_{1}(t)=\frac{14.8487\;t^{\alpha_{ℛ}}}{(\alpha_{ℛ})!}. \end{array}$$

Now, for the second iteration (*n* = 1), the recursive relations become


$$\begin{array}{cc} {𝒮}_{2}(t) & =-{ℒ}^{-1}\;\left[\frac{1}{s^{\alpha_{𝒮}}}\;ℒ\;\left\{0.0001A_{1}+0.0019094\;{𝒮}_{1}-0.002778\;{𝒱}_{1}\right\}\right],\\ {𝒱}_{2}(t) & ={ℒ}^{-1}\;\left[\frac{1}{s^{\alpha_{𝒱}}}\;ℒ\;\left\{0.001884\;{𝒮}_{1}-0.0000001\;B_{1}-0.002803\;{𝒱}_{1}\right\}\right],\\ {ℐ}_{2}(t) & ={ℒ}^{-1}\;\left[\frac{1}{s^{\alpha_{ℐ}}}\;ℒ\;\left\{0.0001A_{1}+0.0000001\;B_{1}-0.09907\;{ℐ}_{1}\right\}\right],\\ {ℛ}_{2}(t) & ={ℒ}^{-1}\;\left[\frac{1}{s^{\alpha_{ℛ}}}\;ℒ\;\left\{0.099\;{ℐ}_{1}-0.00002537\;{ℛ}_{1}\right\}\right]. \end{array}$$ (86)

$$\begin{array}{cc} {𝒮}_{2}(t) & =\frac{0.126523\;t^{2\alpha_{𝒮}}}{(2\alpha_{𝒮})!}+\frac{0.000117266\;t^{3\alpha_{𝒮}}}{(3\alpha_{𝒮})!}-\frac{0.000123065\;t^{\alpha_{𝒮}+\alpha_{𝒱}}}{(\alpha_{𝒮}+\alpha_{𝒱})!}+\frac{2.11013\times 10^{-6}\;t^{2\alpha_{𝒮}+\alpha_{𝒱}}}{(2\alpha_{𝒮}+\alpha_{𝒱})!}-\frac{6.06029\times 10^{-9}\;t^{\alpha_{𝒮}+2\alpha_{𝒱}}}{(\alpha_{𝒮}+2\alpha_{𝒱})!}\\ & \;+\frac{0.367763\;t^{\alpha_{𝒮}+\alpha_{ℐ}}}{(\alpha_{𝒮}+\alpha_{ℐ})!}-\frac{0.000307595\;t^{2\alpha_{𝒮}+\alpha_{ℐ}}}{(2\alpha_{𝒮}+\alpha_{ℐ})!}+\frac{0.000301658\;t^{2\alpha_{𝒮}+\alpha_{ℐ}}\;(\alpha_{𝒮}+\alpha_{ℐ})!}{(\alpha_{𝒮})!(\alpha_{ℐ})!(2\alpha_{𝒮}+\alpha_{ℐ})!}-\frac{2.52305\times 10^{-7}\;t^{3\alpha_{𝒮}+\alpha_{ℐ}}\;(2\alpha_{𝒮}+\alpha_{ℐ})!}{(\alpha_{𝒮})!(\alpha_{𝒮}+\alpha_{ℐ})!(3\alpha_{𝒮}+\alpha_{ℐ})!}\\ & \;-\frac{5.80605\times 10^{-10}\;t^{\alpha_{𝒮}+\alpha_{𝒱}+\alpha_{ℐ}}}{(\alpha_{𝒮}+\alpha_{𝒱}+\alpha_{ℐ})!}-\frac{4.76242\times 10^{-13}\;t^{2\alpha_{𝒮}+\alpha_{𝒱}+\alpha_{ℐ}}\;(\alpha_{𝒮}+\alpha_{𝒱}+\alpha_{ℐ})!}{(\alpha_{𝒮})!(\alpha_{𝒱}+\alpha_{ℐ})!(2\alpha_{𝒮}+\alpha_{𝒱}+\alpha_{ℐ})!}, \end{array}$$

$$\begin{array}{cc} {𝒱}_{2}(t) & =-\frac{0.0140968\;t^{\alpha_{𝒮}+\alpha_{𝒱}}}{(\alpha_{𝒮}+\alpha_{𝒱})!}-\frac{0.0000130655\;t^{2\alpha_{𝒮}+\alpha_{𝒱}}}{(2\alpha_{𝒮}+\alpha_{𝒱})!}+\frac{0.000124837\;t^{2\alpha_{𝒱}}}{(2\alpha_{𝒱})!}-\frac{2.17335\times 10^{-6}\;t^{\alpha_{𝒮}+2\alpha_{𝒱}}}{(\alpha_{𝒮}+2\alpha_{𝒱})!}+\frac{6.14755\times 10^{-9}\;t^{3\alpha_{𝒱}}}{(3\alpha_{𝒱})!}\\ & \;+\frac{0.0002574\;t^{\alpha_{𝒱}+\alpha_{ℐ}}}{(\alpha_{𝒱}+\alpha_{ℐ})!}-\frac{2.153\times 10^{-7}\;t^{\alpha_{𝒮}+\alpha_{𝒱}+\alpha_{ℐ}}}{(\alpha_{𝒮}+\alpha_{𝒱}+\alpha_{ℐ})!}-\frac{4.064\times 10^{-13}\;t^{2\alpha_{𝒱}+\alpha_{ℐ}}}{(2\alpha_{𝒱}+\alpha_{ℐ})!}+\frac{5.694\times 10^{-10}\;t^{2\alpha_{𝒱}+\alpha_{ℐ}}\;(\alpha_{𝒱}+\alpha_{ℐ})!}{(\alpha_{𝒱})!(\alpha_{ℐ})!(2\alpha_{𝒱}+\alpha_{ℐ})!}\\ & \;-\frac{4.76242\times 10^{-13}\;t^{\alpha_{𝒮}+2\alpha_{𝒱}+\alpha_{ℐ}}\;(\alpha_{𝒮}+\alpha_{𝒱}+\alpha_{ℐ})!}{(\alpha_{𝒱})!(\alpha_{𝒮}+\alpha_{ℐ})!(\alpha_{𝒮}+2\alpha_{𝒱}+\alpha_{ℐ})!}-\frac{8.98939\times 10^{-19}\;t^{3\alpha_{𝒱}+\alpha_{ℐ}}\;(2\alpha_{𝒱}+\alpha_{ℐ})!}{(\alpha_{𝒱})!(\alpha_{𝒱}+\alpha_{ℐ})!(3\alpha_{𝒱}+\alpha_{ℐ})!}, \end{array}$$

$$\begin{array}{cc} {ℐ}_{2}(t) & =-\frac{0.112236\;t^{\alpha_{𝒮}+\alpha_{ℐ}}}{(\alpha_{𝒮}+\alpha_{ℐ})!}-\frac{0.000104025\;t^{2\alpha_{𝒮}+\alpha_{ℐ}}}{(2\alpha_{𝒮}+\alpha_{ℐ})!}-\frac{6.645\times 10^{-7}\;t^{\alpha_{𝒱}+\alpha_{ℐ}}}{(\alpha_{𝒱}+\alpha_{ℐ})!}+\frac{4.38486\times 10^{-8}\;t^{\alpha_{𝒮}+\alpha_{𝒱}+\alpha_{ℐ}}}{(\alpha_{𝒮}+\alpha_{𝒱}+\alpha_{ℐ})!}+\frac{0.360665\;t^{2\alpha_{ℐ}}}{(2\alpha_{ℐ})!}\\ & \;-\frac{3.272\times 10^{-11}\;t^{2\alpha_{𝒱}+\alpha_{ℐ}}}{(2\alpha_{𝒱}+\alpha_{ℐ})!}-\frac{0.0003017\;t^{\alpha_{𝒮}+2\alpha_{ℐ}}}{(\alpha_{𝒮}+2\alpha_{ℐ})!}-\frac{0.0003017\;t^{\alpha_{𝒮}+2\alpha_{ℐ}}\;(\alpha_{𝒮}+\alpha_{ℐ})!}{(\alpha_{𝒮})!(\alpha_{ℐ})!(\alpha_{𝒮}+2\alpha_{ℐ})!}+\frac{2.523\times 10^{-7}\;t^{2\alpha_{𝒮}+2\alpha_{ℐ}}\;(2\alpha_{𝒮}+\alpha_{ℐ})!}{(\alpha_{𝒮})!(\alpha_{𝒮}+\alpha_{ℐ})!(2\alpha_{𝒮}+2\alpha_{ℐ})!}\\ & \;-\frac{5.69399\times 10^{-10}\;t^{\alpha_{𝒱}+2\alpha_{ℐ}}}{(\alpha_{𝒱}+2\alpha_{ℐ})!}-\frac{5.69399\times 10^{-10}\;t^{\alpha_{𝒱}+2\alpha_{ℐ}}\;(\alpha_{𝒱}+\alpha_{ℐ})!}{(\alpha_{𝒱})!(\alpha_{ℐ})!(\alpha_{𝒱}+2\alpha_{ℐ})!}+\frac{4.76242\times 10^{-13}\;t^{\alpha_{𝒮}+\alpha_{𝒱}+2\alpha_{ℐ}}\;(\alpha_{𝒮}+\alpha_{𝒱}+\alpha_{ℐ})!}{(\alpha_{𝒱})!(\alpha_{𝒮}+\alpha_{ℐ})!(\alpha_{𝒮}+\alpha_{𝒱}+2\alpha_{ℐ})!}\\ & \;+\frac{4.76242\times 10^{-13}\;t^{\alpha_{𝒮}+\alpha_{𝒱}+2\alpha_{ℐ}}\;(\alpha_{𝒮}+\alpha_{𝒱}+\alpha_{ℐ})!}{(\alpha_{𝒮})!(\alpha_{𝒱}+\alpha_{ℐ})!(\alpha_{𝒮}+\alpha_{𝒱}+2\alpha_{ℐ})!}+\frac{8.98939\times 10^{-19}\;t^{2\alpha_{𝒱}+2\alpha_{ℐ}}\;(2\alpha_{𝒱}+\alpha_{ℐ})!}{(\alpha_{𝒱})!(\alpha_{𝒱}+\alpha_{ℐ})!(2\alpha_{𝒱}+2\alpha_{ℐ})!}, \end{array}$$

$$\begin{array}{c} {ℛ}_{2}(t)=-\frac{0.72817\;t^{\alpha_{ℐ}+\alpha_{ℛ}}}{(\alpha_{ℐ}+\alpha_{ℛ})!}+\frac{0.000609037\;t^{\alpha_{𝒮}+\alpha_{ℐ}+\alpha_{ℛ}}}{(\alpha_{𝒮}+\alpha_{ℐ}+\alpha_{ℛ})!}+\frac{1.1496\times 10^{-9}\;t^{\alpha_{𝒱}+\alpha_{ℐ}+\alpha_{ℛ}}}{(\alpha_{𝒱}+\alpha_{ℐ}+\alpha_{ℛ})!}-\frac{0.000376712\;t^{2\alpha_{ℛ}}}{(2\alpha_{ℛ})!}. \end{array}$$

$$\begin{array}{cc} {𝒮}_{3}(t) & =-\frac{0.00213943\;t^{3\alpha_{𝒮}}}{(3\alpha_{𝒮})!}-\frac{1.9829\times 10^{-6}\;t^{4\alpha_{𝒮}}}{(4\alpha_{𝒮})!}-\frac{0.00003708\;t^{2\alpha_{𝒮}+\alpha_{𝒱}}}{(2\alpha_{𝒮}+\alpha_{𝒱})!}-\frac{7.1977\times 10^{-8}\;t^{3\alpha_{𝒮}+\alpha_{𝒱}}}{(3\alpha_{𝒮}+\alpha_{𝒱})!}+\frac{3.46797\times 10^{-7}\;t^{\alpha_{𝒮}+2\alpha_{𝒱}}}{(\alpha_{𝒮}+2\alpha_{𝒱})!}\\ & \;-\frac{5.93509\times 10^{-9}\;t^{2\alpha_{𝒮}+2\alpha_{𝒱}}}{(2\alpha_{𝒮}+2\alpha_{𝒱})!}+\frac{1.70779\times 10^{-11}\;t^{\alpha_{𝒮}+3\alpha_{𝒱}}}{(\alpha_{𝒮}+3\alpha_{𝒱})!}-\frac{0.000606852\;t^{2\alpha_{𝒮}+\alpha_{ℐ}}}{(2\alpha_{𝒮}+\alpha_{ℐ})!}-\frac{0.00550349\;t^{2\alpha_{𝒮}+\alpha_{ℐ}}(\alpha_{𝒮}+\alpha_{ℐ})!}{(\alpha_{𝒮})!(\alpha_{ℐ})!(2\alpha_{𝒮}+\alpha_{ℐ})!}\\ & \;-\frac{5.1008\times 10^{-6}\;t^{3\alpha_{𝒮}+\alpha_{ℐ}}(\alpha_{𝒮}+\alpha_{ℐ})!}{(\alpha_{𝒮})!(\alpha_{ℐ})!(3\alpha_{𝒮}+\alpha_{ℐ})!}-\frac{5.1008\times 10^{-6}\;t^{3\alpha_{𝒮}+\alpha_{ℐ}}(2\alpha_{𝒮}+\alpha_{ℐ})!}{(2\alpha_{𝒮})!(\alpha_{ℐ})!(3\alpha_{𝒮}+\alpha_{ℐ})!}+\frac{4.26633\times 10^{-9}\;t^{4\alpha_{𝒮}+\alpha_{ℐ}}(2\alpha_{𝒮}+\alpha_{ℐ})!}{(\alpha_{𝒮})!(\alpha_{𝒮}+\alpha_{ℐ})!(4\alpha_{𝒮}+\alpha_{ℐ})!}\\ & \;+\frac{9.20618\times 10^{-6}\;t^{3\alpha_{𝒮}+\alpha_{ℐ}}(2\alpha_{𝒮}+\alpha_{ℐ})!}{(\alpha_{𝒮})!(\alpha_{𝒮}+\alpha_{ℐ})!(3\alpha_{𝒮}+\alpha_{ℐ})!}+\frac{4.26632\times 10^{-9}\;t^{4\alpha_{𝒮}+\alpha_{ℐ}}(3\alpha_{𝒮}+\alpha_{ℐ})!}{(2\alpha_{𝒮})!(\alpha_{𝒮}+\alpha_{ℐ})!(4\alpha_{𝒮}+\alpha_{ℐ})!}+\frac{4.26634\times 10^{-9}\;t^{4\alpha_{𝒮}+\alpha_{ℐ}}(3\alpha_{𝒮}+\alpha_{ℐ})!}{(\alpha_{𝒮})!(2\alpha_{𝒮}+\alpha_{ℐ})!(4\alpha_{𝒮}+\alpha_{ℐ})!}\\ & \;-\frac{1.262\times 10^{-8}\;t^{3\alpha_{𝒮}+2\alpha_{ℐ}}(2\alpha_{𝒮}+\alpha_{ℐ})!}{(\alpha_{𝒮})!(\alpha_{𝒮}+\alpha_{ℐ})!(3\alpha_{𝒮}+2\alpha_{ℐ})!}-\frac{2.78076\times 10^{-9}\;t^{2\alpha_{𝒮}+\alpha_{𝒱}+\alpha_{ℐ}}}{(2\alpha_{𝒮}+\alpha_{𝒱}+\alpha_{ℐ})!}+\frac{1.58179\times 10^{-9}\;t^{2\alpha_{𝒮}+\alpha_{𝒱}+\alpha_{ℐ}}(\alpha_{𝒮}+\alpha_{𝒱}+\alpha_{ℐ})!}{(\alpha_{𝒮}+\alpha_{𝒱})!(\alpha_{ℐ})!(2\alpha_{𝒮}+\alpha_{𝒱}+\alpha_{ℐ})!}\\ & \;+\frac{0.0000104\;t^{3\alpha_{𝒮}+\alpha_{ℐ}}}{(3\alpha_{𝒮}+\alpha_{ℐ})!}+\frac{3.5942\times 10^{-11}\;t^{2\alpha_{𝒮}+\alpha_{𝒱}+\alpha_{ℐ}}(\alpha_{𝒮}+\alpha_{𝒱}+\alpha_{ℐ})!}{(\alpha_{𝒮})!(\alpha_{𝒱}+\alpha_{ℐ})!(2\alpha_{𝒮}+\alpha_{𝒱}+\alpha_{ℐ})!}+\frac{8.053\times 10^{-15}\;t^{3\alpha_{𝒮}+\alpha_{𝒱}+\alpha_{ℐ}}(\alpha_{𝒮}+\alpha_{𝒱}+\alpha_{ℐ})!}{(\alpha_{𝒮})!(\alpha_{𝒱}+\alpha_{ℐ})!(3\alpha_{𝒮}+\alpha_{𝒱}+\alpha_{ℐ})!}\\ & \;-\frac{1.323\times 10^{-12}\;t^{3\alpha_{𝒮}+\alpha_{𝒱}+\alpha_{ℐ}}(2\alpha_{𝒮}+\alpha_{𝒱}+\alpha_{ℐ})!}{(\alpha_{𝒮}+\alpha_{𝒱})!(\alpha_{𝒮}+\alpha_{ℐ})!(3\alpha_{𝒮}+\alpha_{𝒱}+\alpha_{ℐ})!}+\frac{8.05297\times 10^{-15}\;t^{3\alpha_{𝒮}+\alpha_{𝒱}+\alpha_{ℐ}}(2\alpha_{𝒮}+\alpha_{𝒱}+\alpha_{ℐ})!}{(2\alpha_{𝒮})!(\alpha_{𝒱}+\alpha_{ℐ})!(3\alpha_{𝒮}+\alpha_{𝒱}+\alpha_{ℐ})!}\\ & \;-\frac{1.798\times 10^{-12}\;t^{3\alpha_{𝒮}+\alpha_{𝒱}+\alpha_{ℐ}}(2\alpha_{𝒮}+\alpha_{𝒱}+\alpha_{ℐ})!}{(\alpha_{𝒮})!(\alpha_{𝒮}+\alpha_{𝒱}+\alpha_{ℐ})!(3\alpha_{𝒮}+\alpha_{𝒱}+\alpha_{ℐ})!}+\frac{1.635\times 10^{-12}\;t^{\alpha_{𝒮}+2\alpha_{𝒱}+\alpha_{ℐ}}}{(\alpha_{𝒮}+2\alpha_{𝒱}+\alpha_{ℐ})!}+\frac{1.582\times 10^{-12}\;t^{\alpha_{𝒮}+2\alpha_{𝒱}+\alpha_{ℐ}}(\alpha_{𝒱}+\alpha_{ℐ})!}{(\alpha_{𝒱})!(\alpha_{ℐ})!(\alpha_{𝒮}+2\alpha_{𝒱}+\alpha_{ℐ})!}\\ & \;-\frac{1.323\times 10^{-15}\;t^{2\alpha_{𝒮}+2\alpha_{𝒱}+\alpha_{ℐ}}(\alpha_{𝒮}+\alpha_{𝒱}+\alpha_{ℐ})!}{(\alpha_{𝒱})!(\alpha_{𝒮}+\alpha_{ℐ})!(2\alpha_{𝒮}+2\alpha_{𝒱}+\alpha_{ℐ})!}-\frac{2.49725\times 10^{-18}\;t^{2\alpha_{𝒮}+2\alpha_{𝒱}+\alpha_{ℐ}}(\alpha_{𝒮}+2\alpha_{𝒱}+\alpha_{ℐ})!}{(\alpha_{𝒮}+\alpha_{𝒱})!(\alpha_{𝒱}+\alpha_{ℐ})!(2\alpha_{𝒮}+2\alpha_{𝒱}+\alpha_{ℐ})!}\\ & \;+\frac{1.34205\times 10^{-15}\;t^{2\alpha_{𝒮}+2\alpha_{𝒱}+\alpha_{ℐ}}(\alpha_{𝒮}+2\alpha_{𝒱}+\alpha_{ℐ})!}{(\alpha_{𝒮})!(2\alpha_{𝒱}+\alpha_{ℐ})!(2\alpha_{𝒮}+2\alpha_{𝒱}+\alpha_{ℐ})!}-\frac{2.49725\times 10^{-21}\;t^{\alpha_{𝒮}+3\alpha_{𝒱}+\alpha_{ℐ}}(2\alpha_{𝒱}+\alpha_{ℐ})!}{(\alpha_{𝒱})!(\alpha_{𝒱}+\alpha_{ℐ})!(\alpha_{𝒮}+3\alpha_{𝒱}+\alpha_{ℐ})!}\\ & \;-\frac{0.0180333\;t^{\alpha_{𝒮}+2\alpha_{ℐ}}}{(\alpha_{𝒮}+2\alpha_{ℐ})!}+\frac{0.00001508\;t^{2\alpha_{𝒮}+2\alpha_{ℐ}}}{(2\alpha_{𝒮}+2\alpha_{ℐ})!}+\frac{0.00001508\;t^{2\alpha_{𝒮}+2\alpha_{ℐ}}(\alpha_{𝒮}+\alpha_{ℐ})!}{(\alpha_{𝒮})!(\alpha_{ℐ})!(2\alpha_{𝒮}+2\alpha_{ℐ})!}-\frac{0.00001479\;t^{2\alpha_{𝒮}+2\alpha_{ℐ}}(\alpha_{𝒮}+2\alpha_{ℐ})!}{(\alpha_{𝒮})!(2\alpha_{ℐ})!(2\alpha_{𝒮}+2\alpha_{ℐ})!}\\ & \;+\frac{7.48377\times 10^{-7}\;t^{\alpha_{𝒮}+\alpha_{𝒱}+\alpha_{ℐ}}}{(\alpha_{𝒮}+\alpha_{𝒱}+\alpha_{ℐ})!}+\frac{1.237\times 10^{-8}\;t^{3\alpha_{𝒮}+2\alpha_{ℐ}}(2\alpha_{𝒮}+2\alpha_{ℐ})!}{(\alpha_{𝒮})!(\alpha_{𝒮}+2\alpha_{ℐ})!(3\alpha_{𝒮}+2\alpha_{ℐ})!}+\frac{1.237\times 10^{-8}\;t^{3\alpha_{𝒮}+2\alpha_{ℐ}}(\alpha_{𝒮}+\alpha_{ℐ})!(2\alpha_{𝒮}+2\alpha_{ℐ})!}{(\alpha_{𝒮}){!}^{2}(\alpha_{ℐ})!(\alpha_{𝒮}+2\alpha_{ℐ})!(3\alpha_{𝒮}+2\alpha_{ℐ})!}\\ & \;-\frac{1.035\times 10^{-11}\;t^{4\alpha_{𝒮}+2\alpha_{ℐ}}(2\alpha_{𝒮}+\alpha_{ℐ})!(3\alpha_{𝒮}+2\alpha_{ℐ})!}{(\alpha_{𝒮}){!}^{2}(\alpha_{𝒮}+\alpha_{ℐ})!(2\alpha_{𝒮}+2\alpha_{ℐ})!(4\alpha_{𝒮}+2\alpha_{ℐ})!}+\frac{2.85\times 10^{-11}\;t^{\alpha_{𝒮}+\alpha_{𝒱}+2\alpha_{ℐ}}}{(\alpha_{𝒮}+\alpha_{𝒱}+2\alpha_{ℐ})!}+\frac{2.85\times 10^{-11}\;t^{\alpha_{𝒮}+\alpha_{𝒱}+2\alpha_{ℐ}}(\alpha_{𝒱}+\alpha_{ℐ})!}{(\alpha_{𝒱})!(\alpha_{ℐ})!(\alpha_{𝒮}+\alpha_{𝒱}+2\alpha_{ℐ})!}\\ & \;-\frac{2.38121\times 10^{-14}\;t^{2\alpha_{𝒮}+\alpha_{𝒱}+2\alpha_{ℐ}}(\alpha_{𝒮}+\alpha_{𝒱}+\alpha_{ℐ})!}{(\alpha_{𝒱})!(\alpha_{𝒮}+\alpha_{ℐ})!(2\alpha_{𝒮}+\alpha_{𝒱}+2\alpha_{ℐ})!}-\frac{1.95319\times 10^{-17}\;t^{3\alpha_{𝒮}+\alpha_{𝒱}+2\alpha_{ℐ}}(\alpha_{𝒮}+\alpha_{𝒱}+\alpha_{ℐ})!(2\alpha_{𝒮}+\alpha_{𝒱}+2\alpha_{ℐ})!}{(\alpha_{𝒮}){!}^{2}(\alpha_{𝒱}+\alpha_{ℐ})!(\alpha_{𝒮}+\alpha_{𝒱}+2\alpha_{ℐ})!(3\alpha_{𝒮}+\alpha_{𝒱}+2\alpha_{ℐ})!}\\ & \;+\frac{2.33525\times 10^{-14}\;t^{2\alpha_{𝒮}+\alpha_{𝒱}+2\alpha_{ℐ}}(\alpha_{𝒮}+\alpha_{𝒱}+2\alpha_{ℐ})!}{(\alpha_{𝒮})!(\alpha_{𝒱}+2\alpha_{ℐ})!(2\alpha_{𝒮}+\alpha_{𝒱}+2\alpha_{ℐ})!}-\frac{1.95319\times 10^{-17}\;t^{3\alpha_{𝒮}+\alpha_{𝒱}+2\alpha_{ℐ}}(\alpha_{𝒮}+\alpha_{𝒱}+\alpha_{ℐ})!(2\alpha_{𝒮}+\alpha_{𝒱}+2\alpha_{ℐ})!}{(\alpha_{𝒮})!(\alpha_{𝒱})!(\alpha_{𝒮}+\alpha_{ℐ})!(\alpha_{𝒮}+\alpha_{𝒱}+2\alpha_{ℐ})!(3\alpha_{𝒮}+\alpha_{𝒱}+2\alpha_{ℐ})!}\\ & \;+\frac{2.33525\times 10^{-14}\;t^{2\alpha_{𝒮}+\alpha_{𝒱}+2\alpha_{ℐ}}(\alpha_{𝒱}+\alpha_{ℐ})!(\alpha_{𝒮}+\alpha_{𝒱}+2\alpha_{ℐ})!}{(\alpha_{𝒮})!(\alpha_{𝒱})!(\alpha_{ℐ})!(\alpha_{𝒱}+2\alpha_{ℐ})!(2\alpha_{𝒮}+\alpha_{𝒱}+2\alpha_{ℐ})!}-\frac{2.38121\times 10^{-14}\;t^{2\alpha_{𝒮}+\alpha_{𝒱}+2\alpha_{ℐ}}(\alpha_{𝒮}+\alpha_{𝒱}+\alpha_{ℐ})!}{(\alpha_{𝒮})!(\alpha_{𝒱}+\alpha_{ℐ})!(2\alpha_{𝒮}+\alpha_{𝒱}+2\alpha_{ℐ})!}\\ & \;-\frac{4.4947\times 10^{-20}\;t^{\alpha_{𝒮}+2\alpha_{𝒱}+2\alpha_{ℐ}}(2\alpha_{𝒱}+\alpha_{ℐ})!}{(\alpha_{𝒱})!(\alpha_{𝒱}+\alpha_{ℐ})!(\alpha_{𝒮}+2\alpha_{𝒱}+2\alpha_{ℐ})!}-\frac{3.68678\times 10^{-23}\;t^{2\alpha_{𝒮}+2\alpha_{𝒱}+2\alpha_{ℐ}}(2\alpha_{𝒱}+\alpha_{ℐ})!(\alpha_{𝒮}+2\alpha_{𝒱}+2\alpha_{ℐ})!}{(\alpha_{𝒮})!(\alpha_{𝒱})!(\alpha_{𝒱}+\alpha_{ℐ})!(2\alpha_{𝒱}+2\alpha_{ℐ})!(2\alpha_{𝒮}+2\alpha_{𝒱}+2\alpha_{ℐ})!}. \end{array}$$

$$\begin{array}{cc} {𝒱}_{3}(t) & =\frac{0.000238369\;t^{2\alpha_{𝒮}+\alpha_{𝒱}}}{(2\alpha_{𝒮}+\alpha_{𝒱})!}+\frac{2.20929\times 10^{-7}\;t^{3\alpha_{𝒮}+\alpha_{𝒱}}}{(3\alpha_{𝒮}+\alpha_{𝒱})!}+\frac{0.0000395\;t^{\alpha_{𝒮}+2\alpha_{𝒱}}}{(\alpha_{𝒮}+2\alpha_{𝒱})!}+\frac{4.0794\times 10^{-8}\;t^{2\alpha_{𝒮}+2\alpha_{𝒱}}}{(2\alpha_{𝒮}+2\alpha_{𝒱})!}-\frac{3.5179\times 10^{-7}\;t^{3\alpha_{𝒱}}}{(3\alpha_{𝒱})!}\\ & \;+\frac{6.11308\times 10^{-9}\;t^{\alpha_{𝒮}+3\alpha_{𝒱}}}{(\alpha_{𝒮}+3\alpha_{𝒱})!}-\frac{1.73238\times 10^{-11}\;t^{4\alpha_{𝒱}}}{(4\alpha_{𝒱})!}+\frac{0.000696794\;t^{\alpha_{𝒮}+\alpha_{𝒱}+\alpha_{ℐ}}}{(\alpha_{𝒮}+\alpha_{𝒱}+\alpha_{ℐ})!}-\frac{5.75868\times 10^{-7}\;t^{2\alpha_{𝒮}+\alpha_{𝒱}+\alpha_{ℐ}}}{(2\alpha_{𝒮}+\alpha_{𝒱}+\alpha_{ℐ})!}\\ & \;+\frac{5.68324\times 10^{-7}\;t^{2\alpha_{𝒮}+\alpha_{𝒱}+\alpha_{ℐ}}\;(\alpha_{𝒮}+\alpha_{ℐ})!}{(\alpha_{𝒮})!(\alpha_{ℐ})!(2\alpha_{𝒮}+\alpha_{𝒱}+\alpha_{ℐ})!}-\frac{4.75343\times 10^{-10}\;t^{3\alpha_{𝒮}+\alpha_{𝒱}+\alpha_{ℐ}}\;(2\alpha_{𝒮}+\alpha_{ℐ})!}{(\alpha_{𝒮})!(\alpha_{𝒮}+\alpha_{ℐ})!(3\alpha_{𝒮}+\alpha_{𝒱}+\alpha_{ℐ})!}-\frac{7.25426\times 10^{-7}\;t^{2\alpha_{𝒱}+\alpha_{ℐ}}}{(2\alpha_{𝒱}+\alpha_{ℐ})!}\\ & \;-\frac{3.25838\times 10^{-8}\;t^{2\alpha_{𝒱}+\alpha_{ℐ}}\;(\alpha_{𝒱}+\alpha_{ℐ})!}{(\alpha_{𝒱})!(\alpha_{ℐ})!(2\alpha_{𝒱}+\alpha_{ℐ})!}+\frac{6.04132\times 10^{-10}\;t^{\alpha_{𝒮}+2\alpha_{𝒱}+\alpha_{ℐ}}}{(\alpha_{𝒮}+2\alpha_{𝒱}+\alpha_{ℐ})!}+\frac{5.68323\times 10^{-10}\;t^{\alpha_{𝒮}+2\alpha_{𝒱}+\alpha_{ℐ}}\;(\alpha_{𝒮}+\alpha_{𝒱}+\alpha_{ℐ})!}{(\alpha_{𝒮}+\alpha_{𝒱})!(\alpha_{ℐ})!(\alpha_{𝒮}+2\alpha_{𝒱}+\alpha_{ℐ})!}\\ & \;+\frac{3.59415\times 10^{-11}\;t^{\alpha_{𝒮}+2\alpha_{𝒱}+\alpha_{ℐ}}\;(\alpha_{𝒮}+\alpha_{𝒱}+\alpha_{ℐ})!}{(\alpha_{𝒱})!(\alpha_{𝒮}+\alpha_{ℐ})!(\alpha_{𝒮}+2\alpha_{𝒱}+\alpha_{ℐ})!}+\frac{8.05299\times 10^{-15}\;t^{2\alpha_{𝒮}+2\alpha_{𝒱}+\alpha_{ℐ}}\;(2\alpha_{𝒮}+\alpha_{𝒱}+\alpha_{ℐ})!}{(\alpha_{𝒱})!(2\alpha_{𝒮}+\alpha_{ℐ})!(2\alpha_{𝒮}+2\alpha_{𝒱}+\alpha_{ℐ})!}\\ & \;-\frac{4.75342\times 10^{-13}\;t^{2\alpha_{𝒮}+2\alpha_{𝒱}+\alpha_{ℐ}}\;(2\alpha_{𝒮}+\alpha_{𝒱}+\alpha_{ℐ})!}{(\alpha_{𝒮}+\alpha_{𝒱})!(\alpha_{𝒮}+\alpha_{ℐ})!(2\alpha_{𝒮}+2\alpha_{𝒱}+\alpha_{ℐ})!}+\frac{2.2906\times 10^{-15}\;t^{3\alpha_{𝒱}+\alpha_{ℐ}}}{(3\alpha_{𝒱}+\alpha_{ℐ})!}-\frac{1.60457\times 10^{-12}\;t^{3\alpha_{𝒱}+\alpha_{ℐ}}\;(\alpha_{𝒱}+\alpha_{ℐ})!}{(\alpha_{𝒱})!(\alpha_{ℐ})!(3\alpha_{𝒱}+\alpha_{ℐ})!}\\ & \;-\frac{8.9724\times 10^{-16}\;t^{2\alpha_{𝒮}+2\alpha_{𝒱}+\alpha_{ℐ}}\;(\alpha_{𝒮}+\alpha_{𝒱}+\alpha_{ℐ})!}{(\alpha_{𝒮})!(\alpha_{𝒱}+\alpha_{ℐ})!(2\alpha_{𝒮}+2\alpha_{𝒱}+\alpha_{ℐ})!}-\frac{1.60457\times 10^{-12}\;t^{3\alpha_{𝒱}+\alpha_{ℐ}}\;(2\alpha_{𝒱}+\alpha_{ℐ})!}{(2\alpha_{𝒱})!(\alpha_{ℐ})!(3\alpha_{𝒱}+\alpha_{ℐ})!}-\frac{0.0000126233\;t^{\alpha_{𝒱}+2\alpha_{ℐ}}}{(\alpha_{𝒱}+2\alpha_{ℐ})!}\\ & \;+\frac{1.02883\times 10^{-16}\;t^{3\alpha_{𝒱}+\alpha_{ℐ}}\;(2\alpha_{𝒱}+\alpha_{ℐ})!}{(\alpha_{𝒱})!(\alpha_{𝒱}+\alpha_{ℐ})!(3\alpha_{𝒱}+\alpha_{ℐ})!}+\frac{1.34205\times 10^{-15}\;t^{\alpha_{𝒮}+3\alpha_{𝒱}+\alpha_{ℐ}}\;(\alpha_{𝒮}+\alpha_{𝒱}+\alpha_{ℐ})!}{(\alpha_{𝒱})!(\alpha_{𝒮}+\alpha_{ℐ})!(\alpha_{𝒮}+3\alpha_{𝒱}+\alpha_{ℐ})!}+\frac{1.0558\times 10^{-8}\;t^{\alpha_{𝒮}+\alpha_{𝒱}+2\alpha_{ℐ}}}{(\alpha_{𝒮}+\alpha_{𝒱}+2\alpha_{ℐ})!}\\ & \;+\frac{1.34205\times 10^{-15}\;t^{\alpha_{𝒮}+3\alpha_{𝒱}+\alpha_{ℐ}}\;(\alpha_{𝒮}+2\alpha_{𝒱}+\alpha_{ℐ})!}{(2\alpha_{𝒱})!(\alpha_{𝒮}+\alpha_{ℐ})!(\alpha_{𝒮}+3\alpha_{𝒱}+\alpha_{ℐ})!}-\frac{8.9724\times 10^{-19}\;t^{\alpha_{𝒮}+3\alpha_{𝒱}+\alpha_{ℐ}}\;(\alpha_{𝒮}+2\alpha_{𝒱}+\alpha_{ℐ})!}{(\alpha_{𝒮}+\alpha_{𝒱})!(\alpha_{𝒱}+\alpha_{ℐ})!(\alpha_{𝒮}+3\alpha_{𝒱}+\alpha_{ℐ})!}\\ & \;-\frac{3.3945\times 10^{-18}\;t^{\alpha_{𝒮}+3\alpha_{𝒱}+\alpha_{ℐ}}\;(\alpha_{𝒮}+2\alpha_{𝒱}+\alpha_{ℐ})!}{(\alpha_{𝒱})!(\alpha_{𝒮}+\alpha_{𝒱}+\alpha_{ℐ})!(\alpha_{𝒮}+3\alpha_{𝒱}+\alpha_{ℐ})!}+\frac{2.53321\times 10^{-21}\;t^{4\alpha_{𝒱}+\alpha_{ℐ}}\;(2\alpha_{𝒱}+\alpha_{ℐ})!}{(\alpha_{𝒱})!(\alpha_{𝒱}+\alpha_{ℐ})!(4\alpha_{𝒱}+\alpha_{ℐ})!}+\frac{1.9929\times 10^{-14}\;t^{2\alpha_{𝒱}+2\alpha_{ℐ}}}{(2\alpha_{𝒱}+2\alpha_{ℐ})!}\\ & \;+\frac{2.5332\times 10^{-21}\;t^{4\alpha_{𝒱}+\alpha_{ℐ}}\;(3\alpha_{𝒱}+\alpha_{ℐ})!}{(2\alpha_{𝒱})!(\alpha_{𝒱}+\alpha_{ℐ})!(4\alpha_{𝒱}+\alpha_{ℐ})!}+\frac{2.5332\times 10^{-21}\;t^{4\alpha_{𝒱}+\alpha_{ℐ}}\;(3\alpha_{𝒱}+\alpha_{ℐ})!}{(\alpha_{𝒱})!(2\alpha_{𝒱}+\alpha_{ℐ})!(4\alpha_{𝒱}+\alpha_{ℐ})!}+\frac{1.055\times 10^{-8}\;t^{\alpha_{𝒮}+\alpha_{𝒱}+2\alpha_{ℐ}}\;(\alpha_{𝒮}+\alpha_{ℐ})!}{(\alpha_{𝒮})!(\alpha_{ℐ})!(\alpha_{𝒮}+\alpha_{𝒱}+2\alpha_{ℐ})!}\\ & \;-\frac{8.83\times 10^{-12}\;t^{2\alpha_{𝒮}+\alpha_{𝒱}+2\alpha_{ℐ}}\;(2\alpha_{𝒮}+\alpha_{ℐ})!}{(\alpha_{𝒮})!(\alpha_{𝒮}+\alpha_{ℐ})!(2\alpha_{𝒮}+\alpha_{𝒱}+2\alpha_{ℐ})!}+\frac{1.99\times 10^{-14}\;t^{2\alpha_{𝒱}+2\alpha_{ℐ}}\;(\alpha_{𝒱}+\alpha_{ℐ})!}{(\alpha_{𝒱})!(\alpha_{ℐ})!(2\alpha_{𝒱}+2\alpha_{ℐ})!}-\frac{2.792\times 10^{-11}\;t^{2\alpha_{𝒱}+2\alpha_{ℐ}}\;(\alpha_{𝒱}+2\alpha_{ℐ})!}{(\alpha_{𝒱})!(2\alpha_{ℐ})!(2\alpha_{𝒱}+2\alpha_{ℐ})!}\\ & \;-\frac{1.66685\times 10^{-17}\;t^{\alpha_{𝒮}+2\alpha_{𝒱}+2\alpha_{ℐ}}\;(\alpha_{𝒮}+\alpha_{𝒱}+\alpha_{ℐ})!}{(\alpha_{𝒱})!(\alpha_{𝒮}+\alpha_{ℐ})!(\alpha_{𝒮}+2\alpha_{𝒱}+2\alpha_{ℐ})!}-\frac{1.66685\times 10^{-17}\;t^{\alpha_{𝒮}+2\alpha_{𝒱}+2\alpha_{ℐ}}\;(\alpha_{𝒮}+\alpha_{𝒱}+\alpha_{ℐ})!}{(\alpha_{𝒮})!(\alpha_{𝒱}+\alpha_{ℐ})!(\alpha_{𝒮}+2\alpha_{𝒱}+2\alpha_{ℐ})!}\\ & \;+\frac{2.33526\times 10^{-14}\;t^{\alpha_{𝒮}+2\alpha_{𝒱}+2\alpha_{ℐ}}\;(\alpha_{𝒮}+\alpha_{𝒱}+2\alpha_{ℐ})!}{(\alpha_{𝒱})!(\alpha_{𝒮}+2\alpha_{ℐ})!(\alpha_{𝒮}+2\alpha_{𝒱}+2\alpha_{ℐ})!}+\frac{2.33526\times 10^{-14}\;t^{\alpha_{𝒮}+2\alpha_{𝒱}+2\alpha_{ℐ}}\;(\alpha_{𝒮}+\alpha_{ℐ})!(\alpha_{𝒮}+\alpha_{𝒱}+2\alpha_{ℐ})!}{(\alpha_{𝒮})!(\alpha_{𝒱})!(\alpha_{ℐ})!(\alpha_{𝒮}+2\alpha_{ℐ})!(\alpha_{𝒮}+2\alpha_{𝒱}+2\alpha_{ℐ})!}\\ & \;-\frac{1.95319\times 10^{-17}\;t^{2\alpha_{𝒮}+2\alpha_{𝒱}+2\alpha_{ℐ}}\;(2\alpha_{𝒮}+\alpha_{ℐ})!(2\alpha_{𝒮}+\alpha_{𝒱}+2\alpha_{ℐ})!}{(\alpha_{𝒮})!(\alpha_{𝒱})!(\alpha_{𝒮}+\alpha_{ℐ})!(2\alpha_{𝒮}+2\alpha_{ℐ})!(2\alpha_{𝒮}+2\alpha_{𝒱}+2\alpha_{ℐ})!}+\frac{4.40795\times 10^{-20}\;t^{3\alpha_{𝒱}+2\alpha_{ℐ}}\;(\alpha_{𝒱}+\alpha_{ℐ})!(2\alpha_{𝒱}+2\alpha_{ℐ})!}{(\alpha_{𝒱}){!}^{2}(\alpha_{ℐ})!(\alpha_{𝒱}+2\alpha_{ℐ})!(3\alpha_{𝒱}+2\alpha_{ℐ})!}\\ & \;-\frac{3.14629\times 10^{-23}\;t^{3\alpha_{𝒱}+2\alpha_{ℐ}}\;(2\alpha_{𝒱}+\alpha_{ℐ})!}{(\alpha_{𝒱})!(\alpha_{𝒱}+\alpha_{ℐ})!(3\alpha_{𝒱}+2\alpha_{ℐ})!}-\frac{3.68678\times 10^{-23}\;t^{\alpha_{𝒮}+3\alpha_{𝒱}+2\alpha_{ℐ}}\;(\alpha_{𝒮}+\alpha_{𝒱}+\alpha_{ℐ})!(\alpha_{𝒮}+2\alpha_{𝒱}+2\alpha_{ℐ})!}{(\alpha_{𝒱}){!}^{2}(\alpha_{𝒮}+\alpha_{ℐ})!(\alpha_{𝒮}+\alpha_{𝒱}+2\alpha_{ℐ})!(\alpha_{𝒮}+3\alpha_{𝒱}+2\alpha_{ℐ})!}\\ & \;+\frac{4.40795\times 10^{-20}\;t^{3\alpha_{𝒱}+2\alpha_{ℐ}}\;(2\alpha_{𝒱}+2\alpha_{ℐ})!}{(\alpha_{𝒱})!(\alpha_{𝒱}+2\alpha_{ℐ})!(3\alpha_{𝒱}+2\alpha_{ℐ})!}-\frac{3.68678\times 10^{-23}\;t^{\alpha_{𝒮}+3\alpha_{𝒱}+2\alpha_{ℐ}}\;(\alpha_{𝒮}+\alpha_{𝒱}+\alpha_{ℐ})!(\alpha_{𝒮}+2\alpha_{𝒱}+2\alpha_{ℐ})!}{(\alpha_{𝒮})!(\alpha_{𝒱})!(\alpha_{𝒱}+\alpha_{ℐ})!(\alpha_{𝒮}+\alpha_{𝒱}+2\alpha_{ℐ})!(\alpha_{𝒮}+3\alpha_{𝒱}+2\alpha_{ℐ})!}\\ & \;-\frac{6.95905\times 10^{-29}\;t^{4\alpha_{𝒱}+2\alpha_{ℐ}}\;(2\alpha_{𝒱}+\alpha_{ℐ})!(3\alpha_{𝒱}+2\alpha_{ℐ})!}{(\alpha_{𝒱}){!}^{2}(\alpha_{𝒱}+\alpha_{ℐ})!(2\alpha_{𝒱}+2\alpha_{ℐ})!(4\alpha_{𝒱}+2\alpha_{ℐ})!}. \end{array}$$

$$\begin{array}{cc} {ℐ}_{3}(t) & =\frac{0.00189785\;t^{2\alpha_{𝒮}+\alpha_{ℐ}}}{(2\alpha_{𝒮}+\alpha_{ℐ})!}+\frac{1.75899\times 10^{-6}\;t^{3\alpha_{𝒮}+\alpha_{ℐ}}}{(3\alpha_{𝒮}+\alpha_{ℐ})!}-\frac{2.05743\times 10^{-6}\;t^{\alpha_{𝒮}+\alpha_{𝒱}+\alpha_{ℐ}}}{(\alpha_{𝒮}+\alpha_{𝒱}+\alpha_{ℐ})!}+\frac{3.1456\times 10^{-8}\;t^{2\alpha_{𝒮}+\alpha_{𝒱}+\alpha_{ℐ}}}{(2\alpha_{𝒮}+\alpha_{𝒱}+\alpha_{ℐ})!}\\ & \;+\frac{1.87256\times 10^{-9}\;t^{2\alpha_{𝒱}+\alpha_{ℐ}}}{(2\alpha_{𝒱}+\alpha_{ℐ})!}-\frac{1.23505\times 10^{-10}\;t^{\alpha_{𝒮}+2\alpha_{𝒱}+\alpha_{ℐ}}}{(\alpha_{𝒮}+2\alpha_{𝒱}+\alpha_{ℐ})!}+\frac{9.22133\times 10^{-14}\;t^{3\alpha_{𝒱}+\alpha_{ℐ}}}{(3\alpha_{𝒱}+\alpha_{ℐ})!}+\frac{0.0110199\;t^{\alpha_{𝒮}+2\alpha_{ℐ}}}{(\alpha_{𝒮}+2\alpha_{ℐ})!}\\ & \;+\frac{4.86941\times 10^{-7}\;t^{2\alpha_{𝒮}+2\alpha_{ℐ}}}{(2\alpha_{𝒮}+2\alpha_{ℐ})!}+\frac{4.52487\times 10^{-6}\;t^{2\alpha_{𝒮}+2\alpha_{ℐ}}\;(\alpha_{𝒮}+\alpha_{ℐ})!}{(\alpha_{𝒮})!(\alpha_{ℐ})!(2\alpha_{𝒮}+2\alpha_{ℐ})!}+\frac{5.10085\times 10^{-6}\;t^{2\alpha_{𝒮}+2\alpha_{ℐ}}\;(2\alpha_{𝒮}+\alpha_{ℐ})!}{(2\alpha_{𝒮})!(\alpha_{ℐ})!(2\alpha_{𝒮}+2\alpha_{ℐ})!}\\ & \;+\frac{0.00550349\;t^{\alpha_{𝒮}+2\alpha_{ℐ}}\;(\alpha_{𝒮}+\alpha_{ℐ})!}{(\alpha_{𝒮})!(\alpha_{ℐ})!(\alpha_{𝒮}+2\alpha_{ℐ})!}-\frac{9.206\times 10^{-6}\;t^{2\alpha_{𝒮}+2\alpha_{ℐ}}\;(2\alpha_{𝒮}+\alpha_{ℐ})!}{(\alpha_{𝒮})!(\alpha_{𝒮}+\alpha_{ℐ})!(2\alpha_{𝒮}+2\alpha_{ℐ})!}-\frac{3.785\times 10^{-9}\;t^{3\alpha_{𝒮}+2\alpha_{ℐ}}\;(2\alpha_{𝒮}+\alpha_{ℐ})!}{(\alpha_{𝒮})!(\alpha_{𝒮}+\alpha_{ℐ})!(3\alpha_{𝒮}+2\alpha_{ℐ})!}\\ & \;-\frac{4.26632\times 10^{-9}\;t^{3\alpha_{𝒮}+2\alpha_{ℐ}}\;(3\alpha_{𝒮}+\alpha_{ℐ})!}{(2\alpha_{𝒮})!(\alpha_{𝒮}+\alpha_{ℐ})!(3\alpha_{𝒮}+2\alpha_{ℐ})!}-\frac{4.26634\times 10^{-9}\;t^{3\alpha_{𝒮}+2\alpha_{ℐ}}\;(3\alpha_{𝒮}+\alpha_{ℐ})!}{(\alpha_{𝒮})!(2\alpha_{𝒮}+\alpha_{ℐ})!(3\alpha_{𝒮}+2\alpha_{ℐ})!}+\frac{3.64453\times 10^{-8}\;t^{\alpha_{𝒱}+2\alpha_{ℐ}}}{(\alpha_{𝒱}+2\alpha_{ℐ})!}\\ & \;+\frac{3.258\times 10^{-8}\;t^{\alpha_{𝒱}+2\alpha_{ℐ}}\;(\alpha_{𝒱}+\alpha_{ℐ})!}{(\alpha_{𝒱})!(\alpha_{ℐ})!(\alpha_{𝒱}+2\alpha_{ℐ})!}-\frac{2.162\times 10^{-9}\;t^{\alpha_{𝒮}+\alpha_{𝒱}+2\alpha_{ℐ}}}{(\alpha_{𝒮}+\alpha_{𝒱}+2\alpha_{ℐ})!}-\frac{2.1501\times 10^{-9}\;t^{\alpha_{𝒮}+\alpha_{𝒱}+2\alpha_{ℐ}}\;(\alpha_{𝒮}+\alpha_{𝒱}+\alpha_{ℐ})!}{(\alpha_{𝒮}+\alpha_{𝒱})!(\alpha_{ℐ})!(\alpha_{𝒮}+\alpha_{𝒱}+2\alpha_{ℐ})!}\\ & \;-\frac{3.59415\times 10^{-11}\;t^{\alpha_{𝒮}+\alpha_{𝒱}+2\alpha_{ℐ}}\;(\alpha_{𝒮}+\alpha_{𝒱}+\alpha_{ℐ})!}{(\alpha_{𝒱})!(\alpha_{𝒮}+\alpha_{ℐ})!(\alpha_{𝒮}+\alpha_{𝒱}+2\alpha_{ℐ})!}-\frac{3.59415\times 10^{-11}\;t^{\alpha_{𝒮}+\alpha_{𝒱}+2\alpha_{ℐ}}\;(\alpha_{𝒮}+\alpha_{𝒱}+\alpha_{ℐ})!}{(\alpha_{𝒮})!(\alpha_{𝒱}+\alpha_{ℐ})!(\alpha_{𝒮}+\alpha_{𝒱}+2\alpha_{ℐ})!}\\ & \;-\frac{7.14363\times 10^{-15}\;t^{2\alpha_{𝒮}+\alpha_{𝒱}+2\alpha_{ℐ}}\;(\alpha_{𝒮}+\alpha_{𝒱}+\alpha_{ℐ})!}{(\alpha_{𝒮})!(\alpha_{𝒱}+\alpha_{ℐ})!(2\alpha_{𝒮}+\alpha_{𝒱}+2\alpha_{ℐ})!}+\frac{1.79834\times 10^{-12}\;t^{2\alpha_{𝒮}+\alpha_{𝒱}+2\alpha_{ℐ}}\;(2\alpha_{𝒮}+\alpha_{𝒱}+\alpha_{ℐ})!}{(\alpha_{𝒮}+\alpha_{𝒱})!(\alpha_{𝒮}+\alpha_{ℐ})!(2\alpha_{𝒮}+\alpha_{𝒱}+2\alpha_{ℐ})!}\\ & \;-\frac{8.05299\times 10^{-15}\;t^{2\alpha_{𝒮}+\alpha_{𝒱}+2\alpha_{ℐ}}\;(2\alpha_{𝒮}+\alpha_{𝒱}+\alpha_{ℐ})!}{(\alpha_{𝒱})!(2\alpha_{𝒮}+\alpha_{ℐ})!(2\alpha_{𝒮}+\alpha_{𝒱}+2\alpha_{ℐ})!}-\frac{8.05297\times 10^{-15}\;t^{2\alpha_{𝒮}+\alpha_{𝒱}+2\alpha_{ℐ}}\;(2\alpha_{𝒮}+\alpha_{𝒱}+\alpha_{ℐ})!}{(2\alpha_{𝒮})!(\alpha_{𝒱}+\alpha_{ℐ})!(2\alpha_{𝒮}+\alpha_{𝒱}+2\alpha_{ℐ})!}\\ & \;+\frac{1.798\times 10^{-12}\;t^{2\alpha_{𝒮}+\alpha_{𝒱}+2\alpha_{ℐ}}\;(2\alpha_{𝒮}+\alpha_{𝒱}+\alpha_{ℐ})!}{(\alpha_{𝒮})!(\alpha_{𝒮}+\alpha_{𝒱}+\alpha_{ℐ})!(2\alpha_{𝒮}+\alpha_{𝒱}+2\alpha_{ℐ})!}+\frac{1.605\times 10^{-12}\;t^{2\alpha_{𝒱}+2\alpha_{ℐ}}}{(2\alpha_{𝒱}+2\alpha_{ℐ})!}+\frac{8.541\times 10^{-15}\;t^{2\alpha_{𝒱}+2\alpha_{ℐ}}\;(\alpha_{𝒱}+\alpha_{ℐ})!}{(\alpha_{𝒱})!(\alpha_{ℐ})!(2\alpha_{𝒱}+2\alpha_{ℐ})!}\\ & \;+\frac{1.60457\times 10^{-12}\;t^{2\alpha_{𝒱}+2\alpha_{ℐ}}\;(2\alpha_{𝒱}+\alpha_{ℐ})!}{(2\alpha_{𝒱})!(\alpha_{ℐ})!(2\alpha_{𝒱}+2\alpha_{ℐ})!}-\frac{1.02883\times 10^{-16}\;t^{2\alpha_{𝒱}+2\alpha_{ℐ}}\;(2\alpha_{𝒱}+\alpha_{ℐ})!}{(\alpha_{𝒱})!(\alpha_{𝒱}+\alpha_{ℐ})!(2\alpha_{𝒱}+2\alpha_{ℐ})!}+\frac{0.0147918\;t^{\alpha_{𝒮}+3\alpha_{ℐ}}}{(\alpha_{𝒮}+3\alpha_{ℐ})!}\\ & \;-\frac{7.14363\times 10^{-18}\;t^{\alpha_{𝒮}+2\alpha_{𝒱}+2\alpha_{ℐ}}\;(\alpha_{𝒮}+\alpha_{𝒱}+\alpha_{ℐ})!}{(\alpha_{𝒱})!(\alpha_{𝒮}+\alpha_{ℐ})!(\alpha_{𝒮}+2\alpha_{𝒱}+2\alpha_{ℐ})!}-\frac{1.34205\times 10^{-15}\;t^{\alpha_{𝒮}+2\alpha_{𝒱}+2\alpha_{ℐ}}\;(\alpha_{𝒮}+2\alpha_{𝒱}+\alpha_{ℐ})!}{(2\alpha_{𝒱})!(\alpha_{𝒮}+\alpha_{ℐ})!(\alpha_{𝒮}+2\alpha_{𝒱}+2\alpha_{ℐ})!}\\ & \;+\frac{3.39449\times 10^{-18}\;t^{\alpha_{𝒮}+2\alpha_{𝒱}+2\alpha_{ℐ}}\;(\alpha_{𝒮}+2\alpha_{𝒱}+\alpha_{ℐ})!}{(\alpha_{𝒮}+\alpha_{𝒱})!(\alpha_{𝒱}+\alpha_{ℐ})!(\alpha_{𝒮}+2\alpha_{𝒱}+2\alpha_{ℐ})!}+\frac{3.3945\times 10^{-18}\;t^{\alpha_{𝒮}+2\alpha_{𝒱}+2\alpha_{ℐ}}\;(\alpha_{𝒮}+2\alpha_{𝒱}+\alpha_{ℐ})!}{(\alpha_{𝒱})!(\alpha_{𝒮}+\alpha_{𝒱}+\alpha_{ℐ})!(\alpha_{𝒮}+2\alpha_{𝒱}+2\alpha_{ℐ})!}\\ & \;-\frac{1.348\times 10^{-23}\;t^{3\alpha_{𝒱}+2\alpha_{ℐ}}\;(2\alpha_{𝒱}+\alpha_{ℐ})!}{(\alpha_{𝒱})!(\alpha_{𝒱}+\alpha_{ℐ})!(3\alpha_{𝒱}+2\alpha_{ℐ})!}-\frac{2.53\times 10^{-21}\;t^{3\alpha_{𝒱}+2\alpha_{ℐ}}\;(3\alpha_{𝒱}+\alpha_{ℐ})!}{(2\alpha_{𝒱})!(\alpha_{𝒱}+\alpha_{ℐ})!(3\alpha_{𝒱}+2\alpha_{ℐ})!}-\frac{2.53\times 10^{-21}\;t^{3\alpha_{𝒱}+2\alpha_{ℐ}}\;(3\alpha_{𝒱}+\alpha_{ℐ})!}{(\alpha_{𝒱})!(2\alpha_{𝒱}+\alpha_{ℐ})!(3\alpha_{𝒱}+2\alpha_{ℐ})!}\\ & \;-\frac{0.0176852\;t^{3\alpha_{ℐ}}}{(3\alpha_{ℐ})!}-\frac{1.23718\times 10^{-8}\;t^{2\alpha_{𝒮}+3\alpha_{ℐ}}\;(2\alpha_{𝒮}+2\alpha_{ℐ})!}{(\alpha_{𝒮})!(\alpha_{𝒮}+2\alpha_{ℐ})!(2\alpha_{𝒮}+3\alpha_{ℐ})!}-\frac{1.23718\times 10^{-8}\;t^{2\alpha_{𝒮}+3\alpha_{ℐ}}\;(\alpha_{𝒮}+\alpha_{ℐ})!(2\alpha_{𝒮}+2\alpha_{ℐ})!}{(\alpha_{𝒮}){!}^{2}(\alpha_{ℐ})!(\alpha_{𝒮}+2\alpha_{ℐ})!(2\alpha_{𝒮}+3\alpha_{ℐ})!}\\ & \;+\frac{1.03477\times 10^{-11}\;t^{3\alpha_{𝒮}+3\alpha_{ℐ}}\;(2\alpha_{𝒮}+\alpha_{ℐ})!(3\alpha_{𝒮}+2\alpha_{ℐ})!}{(\alpha_{𝒮}){!}^{2}(\alpha_{𝒮}+\alpha_{ℐ})!(2\alpha_{𝒮}+2\alpha_{ℐ})!(3\alpha_{𝒮}+3\alpha_{ℐ})!}+\frac{2.79205\times 10^{-11}\;t^{\alpha_{𝒱}+3\alpha_{ℐ}}}{(\alpha_{𝒱}+3\alpha_{ℐ})!}+\frac{0.0000147918\;t^{\alpha_{𝒮}+3\alpha_{ℐ}}\;(\alpha_{𝒮}+\alpha_{ℐ})!}{(\alpha_{𝒮})!(\alpha_{ℐ})!(\alpha_{𝒮}+3\alpha_{ℐ})!}\\ & \;+\frac{2.792\times 10^{-11}\;t^{\alpha_{𝒱}+3\alpha_{ℐ}}\;(\alpha_{𝒱}+\alpha_{ℐ})!}{(\alpha_{𝒱})!(\alpha_{ℐ})!(\alpha_{𝒱}+3\alpha_{ℐ})!}+\frac{2.792\times 10^{-11}\;t^{\alpha_{𝒱}+3\alpha_{ℐ}}\;(\alpha_{𝒱}+2\alpha_{ℐ})!}{(\alpha_{𝒱})!(\alpha_{𝒱}+\alpha_{ℐ})!(\alpha_{𝒱}+3\alpha_{ℐ})!}-\frac{1.237\times 10^{-8}\;t^{2\alpha_{𝒮}+3\alpha_{ℐ}}\;(2\alpha_{𝒮}+\alpha_{ℐ})!}{(\alpha_{𝒮})!(\alpha_{𝒮}+\alpha_{ℐ})!(2\alpha_{𝒮}+3\alpha_{ℐ})!}\\ & \;-\frac{2.33525\times 10^{-14}\;t^{\alpha_{𝒮}+\alpha_{𝒱}+3\alpha_{ℐ}}\;(\alpha_{𝒮}+\alpha_{𝒱}+\alpha_{ℐ})!}{(\alpha_{𝒱})!(\alpha_{𝒮}+\alpha_{ℐ})!(\alpha_{𝒮}+\alpha_{𝒱}+3\alpha_{ℐ})!}-\frac{2.33525\times 10^{-14}\;t^{\alpha_{𝒮}+\alpha_{𝒱}+3\alpha_{ℐ}}\;(\alpha_{𝒮}+\alpha_{𝒱}+\alpha_{ℐ})!}{(\alpha_{𝒮})!(\alpha_{𝒱}+\alpha_{ℐ})!(\alpha_{𝒮}+\alpha_{𝒱}+3\alpha_{ℐ})!}\\ & \;-\frac{2.33526\times 10^{-14}\;t^{\alpha_{𝒮}+\alpha_{𝒱}+3\alpha_{ℐ}}\;(\alpha_{𝒮}+\alpha_{𝒱}+2\alpha_{ℐ})!}{(\alpha_{𝒱})!(\alpha_{𝒮}+2\alpha_{ℐ})!(\alpha_{𝒮}+\alpha_{𝒱}+3\alpha_{ℐ})!}-\frac{2.33526\times 10^{-14}\;t^{\alpha_{𝒮}+\alpha_{𝒱}+3\alpha_{ℐ}}\;(\alpha_{𝒮}+\alpha_{ℐ})!(\alpha_{𝒮}+\alpha_{𝒱}+2\alpha_{ℐ})!}{(\alpha_{𝒮})!(\alpha_{𝒱})!(\alpha_{ℐ})!(\alpha_{𝒮}+2\alpha_{ℐ})!(\alpha_{𝒮}+\alpha_{𝒱}+3\alpha_{ℐ})!}\\ & \;-\frac{2.33525\times 10^{-14}\;t^{\alpha_{𝒮}+\alpha_{𝒱}+3\alpha_{ℐ}}\;(\alpha_{𝒮}+\alpha_{𝒱}+2\alpha_{ℐ})!}{(\alpha_{𝒮})!(\alpha_{𝒱}+2\alpha_{ℐ})!(\alpha_{𝒮}+\alpha_{𝒱}+3\alpha_{ℐ})!}-\frac{2.33525\times 10^{-14}\;t^{\alpha_{𝒮}+\alpha_{𝒱}+3\alpha_{ℐ}}\;(\alpha_{𝒱}+\alpha_{ℐ})!(\alpha_{𝒮}+\alpha_{𝒱}+2\alpha_{ℐ})!}{(\alpha_{𝒮})!(\alpha_{𝒱})!(\alpha_{ℐ})!(\alpha_{𝒱}+2\alpha_{ℐ})!(\alpha_{𝒮}+\alpha_{𝒱}+3\alpha_{ℐ})!}\\ & \;-\frac{2.33525\times 10^{-14}\;t^{\alpha_{𝒮}+\alpha_{𝒱}+3\alpha_{ℐ}}\;(\alpha_{𝒮}+\alpha_{𝒱}+\alpha_{ℐ})!}{(\alpha_{𝒮})!(\alpha_{𝒱}+\alpha_{ℐ})!(\alpha_{𝒮}+\alpha_{𝒱}+3\alpha_{ℐ})!}+\frac{1.95319\times 10^{-17}\;t^{2\alpha_{𝒮}+\alpha_{𝒱}+3\alpha_{ℐ}}\;(\alpha_{𝒮}+\alpha_{𝒱}+\alpha_{ℐ})!(2\alpha_{𝒮}+\alpha_{𝒱}+2\alpha_{ℐ})!}{(\alpha_{𝒮})!(\alpha_{𝒱})!(\alpha_{𝒮}+\alpha_{ℐ})!(\alpha_{𝒮}+\alpha_{𝒱}+2\alpha_{ℐ})!(2\alpha_{𝒮}+\alpha_{𝒱}+3\alpha_{ℐ})!}\\ & \;+\frac{1.95319\times 10^{-17}\;t^{2\alpha_{𝒮}+\alpha_{𝒱}+3\alpha_{ℐ}}\;(\alpha_{𝒮}+\alpha_{𝒱}+\alpha_{ℐ})!(2\alpha_{𝒮}+\alpha_{𝒱}+2\alpha_{ℐ})!}{(\alpha_{𝒮}){!}^{2}(\alpha_{𝒱}+\alpha_{ℐ})!(\alpha_{𝒮}+\alpha_{𝒱}+2\alpha_{ℐ})!(2\alpha_{𝒮}+\alpha_{𝒱}+3\alpha_{ℐ})!}+\frac{0.0000147918\;t^{\alpha_{𝒮}+3\alpha_{ℐ}}\;(\alpha_{𝒮}+2\alpha_{ℐ})!}{(\alpha_{𝒮})!(\alpha_{𝒮}+\alpha_{ℐ})!(\alpha_{𝒮}+3\alpha_{ℐ})!}\\ & \;+\frac{1.95319\times 10^{-17}\;t^{2\alpha_{𝒮}+\alpha_{𝒱}+3\alpha_{ℐ}}\;(2\alpha_{𝒮}+\alpha_{ℐ})!(2\alpha_{𝒮}+\alpha_{𝒱}+2\alpha_{ℐ})!}{(\alpha_{𝒮})!(\alpha_{𝒱})!(\alpha_{𝒮}+\alpha_{ℐ})!(2\alpha_{𝒮}+2\alpha_{ℐ})!(2\alpha_{𝒮}+\alpha_{𝒱}+3\alpha_{ℐ})!}-\frac{4.40795\times 10^{-20}\;t^{2\alpha_{𝒱}+3\alpha_{ℐ}}\;(2\alpha_{𝒱}+\alpha_{ℐ})!}{(\alpha_{𝒱})!(\alpha_{𝒱}+\alpha_{ℐ})!(2\alpha_{𝒱}+3\alpha_{ℐ})!}\\ & \;-\frac{4.40795\times 10^{-20}\;t^{2\alpha_{𝒱}+3\alpha_{ℐ}}\;(2\alpha_{𝒱}+2\alpha_{ℐ})!}{(\alpha_{𝒱})!(\alpha_{𝒱}+2\alpha_{ℐ})!(2\alpha_{𝒱}+3\alpha_{ℐ})!}+\frac{3.68678\times 10^{-23}\;t^{\alpha_{𝒮}+2\alpha_{𝒱}+3\alpha_{ℐ}}\;(\alpha_{𝒮}+\alpha_{𝒱}+\alpha_{ℐ})!(\alpha_{𝒮}+2\alpha_{𝒱}+2\alpha_{ℐ})!}{(\alpha_{𝒱}){!}^{2}(\alpha_{𝒮}+\alpha_{ℐ})!(\alpha_{𝒮}+\alpha_{𝒱}+2\alpha_{ℐ})!(\alpha_{𝒮}+2\alpha_{𝒱}+3\alpha_{ℐ})!}\\ & \;-\frac{4.40795\times 10^{-20}\;t^{2\alpha_{𝒱}+3\alpha_{ℐ}}\;(\alpha_{𝒱}+\alpha_{ℐ})!(2\alpha_{𝒱}+2\alpha_{ℐ})!}{(\alpha_{𝒱}){!}^{2}(\alpha_{ℐ})!(\alpha_{𝒱}+2\alpha_{ℐ})!(2\alpha_{𝒱}+3\alpha_{ℐ})!}+\frac{6.95905\times 10^{-29}\;t^{3\alpha_{𝒱}+3\alpha_{ℐ}}\;(2\alpha_{𝒱}+\alpha_{ℐ})!(3\alpha_{𝒱}+2\alpha_{ℐ})!}{(\alpha_{𝒱}){!}^{2}(\alpha_{𝒱}+\alpha_{ℐ})!(2\alpha_{𝒱}+2\alpha_{ℐ})!(3\alpha_{𝒱}+3\alpha_{ℐ})!}. \end{array}$$

$$\begin{array}{cc} {ℛ}_{3}(t) & =-\frac{0.0111114\;t^{\alpha_{𝒮}+\alpha_{ℐ}+\alpha_{ℛ}}}{(\alpha_{𝒮}+\alpha_{ℐ}+\alpha_{ℛ})!}-\frac{0.0000102985\;t^{2\alpha_{𝒮}+\alpha_{ℐ}+\alpha_{ℛ}}}{(2\alpha_{𝒮}+\alpha_{ℐ}+\alpha_{ℛ})!}-\frac{6.57855\times 10^{-8}\;t^{\alpha_{𝒱}+\alpha_{ℐ}+\alpha_{ℛ}}}{(\alpha_{𝒱}+\alpha_{ℐ}+\alpha_{ℛ})!}+\frac{4.34101\times 10^{-9}\;t^{\alpha_{𝒮}+\alpha_{𝒱}+\alpha_{ℐ}+\alpha_{ℛ}}}{(\alpha_{𝒮}+\alpha_{𝒱}+\alpha_{ℐ}+\alpha_{ℛ})!}\\ & \;-\frac{3.2395\times 10^{-12}\;t^{2\alpha_{𝒱}+\alpha_{ℐ}+\alpha_{ℛ}}}{(2\alpha_{𝒱}+\alpha_{ℐ}+\alpha_{ℛ})!}+\frac{0.035706\;t^{2\alpha_{ℐ}+\alpha_{ℛ}}}{(2\alpha_{ℐ}+\alpha_{ℛ})!}-\frac{0.000029864\;t^{\alpha_{𝒮}+2\alpha_{ℐ}+\alpha_{ℛ}}}{(\alpha_{𝒮}+2\alpha_{ℐ}+\alpha_{ℛ})!}-\frac{0.000029864\;t^{\alpha_{𝒮}+2\alpha_{ℐ}+\alpha_{ℛ}}(\alpha_{𝒮}+\alpha_{ℐ})!}{(\alpha_{𝒮})!(\alpha_{ℐ})!(\alpha_{𝒮}+2\alpha_{ℐ}+\alpha_{ℛ})!}\\ & \;+\frac{2.49782\times 10^{-8}\;t^{2\alpha_{𝒮}+2\alpha_{ℐ}+\alpha_{ℛ}}(2\alpha_{𝒮}+\alpha_{ℐ})!}{(\alpha_{𝒮})!(\alpha_{𝒮}+\alpha_{ℐ})!(2\alpha_{𝒮}+2\alpha_{ℐ}+\alpha_{ℛ})!}-\frac{5.63705\times 10^{-11}\;t^{\alpha_{𝒱}+2\alpha_{ℐ}+\alpha_{ℛ}}}{(\alpha_{𝒱}+2\alpha_{ℐ}+\alpha_{ℛ})!}-\frac{5.63705\times 10^{-11}\;t^{\alpha_{𝒱}+2\alpha_{ℐ}+\alpha_{ℛ}}(\alpha_{𝒱}+\alpha_{ℐ})!}{(\alpha_{𝒱})!(\alpha_{ℐ})!(\alpha_{𝒱}+2\alpha_{ℐ}+\alpha_{ℛ})!}\\ & \;+\frac{4.7148\times 10^{-14}\;t^{\alpha_{𝒮}+\alpha_{𝒱}+2\alpha_{ℐ}+\alpha_{ℛ}}(\alpha_{𝒮}+\alpha_{𝒱}+\alpha_{ℐ})!}{(\alpha_{𝒱})!(\alpha_{𝒮}+\alpha_{ℐ})!(\alpha_{𝒮}+\alpha_{𝒱}+2\alpha_{ℐ}+\alpha_{ℛ})!}+\frac{4.7148\times 10^{-14}\;t^{\alpha_{𝒮}+\alpha_{𝒱}+2\alpha_{ℐ}+\alpha_{ℛ}}(\alpha_{𝒮}+\alpha_{𝒱}+\alpha_{ℐ})!}{(\alpha_{𝒮})!(\alpha_{𝒱}+\alpha_{ℐ})!(\alpha_{𝒮}+\alpha_{𝒱}+2\alpha_{ℐ}+\alpha_{ℛ})!}\\ & \;+\frac{8.8995\times 10^{-20}\;t^{2\alpha_{𝒱}+2\alpha_{ℐ}+\alpha_{ℛ}}(2\alpha_{𝒱}+\alpha_{ℐ})!}{(\alpha_{𝒱})!(\alpha_{𝒱}+\alpha_{ℐ})!(2\alpha_{𝒱}+2\alpha_{ℐ}+\alpha_{ℛ})!}+\frac{0.0000184737\;t^{\alpha_{ℐ}+2\alpha_{ℛ}}}{(\alpha_{ℐ}+2\alpha_{ℛ})!}-\frac{1.54513\times 10^{-8}\;t^{\alpha_{𝒮}+\alpha_{ℐ}+2\alpha_{ℛ}}}{(\alpha_{𝒮}+\alpha_{ℐ}+2\alpha_{ℛ})!}\\ & \;-\frac{2.91654\times 10^{-14}\;t^{\alpha_{𝒱}+\alpha_{ℐ}+2\alpha_{ℛ}}}{(\alpha_{𝒱}+\alpha_{ℐ}+2\alpha_{ℛ})!}+\frac{9.55718\times 10^{-9}\;t^{3\alpha_{ℛ}}}{(3\alpha_{ℛ})!}. \end{array}$$

Hence, the approximate LADM solution, obtained by summing the iterative components of 𝒮,𝒱,ℐ,ℛ, is given by:


$$\begin{array}{cc} 𝒮(t) & =500+\frac{0.410126\;t^{\alpha_{𝒮}}}{(\alpha_{𝒮})!}-\frac{7.4824\;t^{\alpha_{𝒮}}}{(\alpha_{𝒮})!}-\frac{0.00693498\;t^{2\alpha_{𝒮}}}{(2\alpha_{𝒮})!}+\frac{2.15056\times 10^{-6}\;t^{\alpha_{𝒮}+\alpha_{𝒱}}}{(\alpha_{𝒮}+\alpha_{𝒱})!}+\cdots ,\\ 𝒱(t) & =350+\frac{0.00077414\;t^{\alpha_{𝒱}}}{(\alpha_{𝒱})!}-\frac{0.0443\;t^{\alpha_{𝒱}}}{(\alpha_{𝒱})!}+\frac{0.000772677\;t^{\alpha_{𝒮}+\alpha_{𝒱}}}{(\alpha_{𝒮}+\alpha_{𝒱})!}-\frac{2.18153\times 10^{-6}\;t^{2\alpha_{𝒱}}}{(2\alpha_{𝒱})!}+\cdots ,\\ ℐ(t) & =150-\frac{7.35525\;t^{\alpha_{ℐ}}}{(\alpha_{ℐ})!}+\frac{0.00615189\;t^{\alpha_{𝒮}+\alpha_{ℐ}}}{(\alpha_{𝒮}+\alpha_{ℐ})!}+\frac{1.16121\times 10^{-8}\;t^{\alpha_{𝒱}+\alpha_{ℐ}}}{(\alpha_{𝒱}+\alpha_{ℐ})!}+\cdots ,\\ ℛ(t) & =50+\frac{14.8487\;t^{\alpha_{ℛ}}}{(\alpha_{ℛ})!}+\cdots . \end{array}$$ (87)

## 8 Results discussion

The numerical results presented in Tables 2–5 illustrate the dynamical behavior of the susceptible 𝒮(t), vaccinated 𝒱(t), infected ℐ(t), and recovered ℛ(t) populations for different homogeneous fractional orders (0.6, 0.65, 0.7, 0.75, 0.8, 0.85, 0.9, 0.95,1), where the solutions are obtained using the Laplace Adomian Decomposition Method. It is observed that the fractional order significantly affects the epidemic dynamics due to the presence of memory effects.In particular, the susceptible population decreases over time, with a slower rate of decline observed for lower fractional orders compared to the classical case. The vaccinated population remains relatively stable, exhibiting only minor variations depending on the fractional order, while showing slightly higher long-term values for smaller orders. The infected population decreases more slowly with lower fractional orders, indicating a longer persistence of the infection within the system.. The recovered class increases with time for all cases on the other hand, but slower for lower fractional orders. Finally, the presented results show that the reduction of the fractional order enhances memory effects in the system, leading to a delayed decay of infection and to a change of the recovery dynamics. It means that the fractional-order SVIR model is more flexible and effective than the classical integer-order model to model the disease transmission.

**Table 2:** Numerical values of the susceptible population 𝒮(t) for homogeneous fractional orders.

*t*	$\alpha_{𝒮}=1$ $\alpha_{𝒱}=1$ $\alpha_{ℐ}=1$ $\alpha_{ℛ}=1$	$\alpha_{𝒮}=0.95$ $\alpha_{𝒱}=0.95$ $\alpha_{ℐ}=0.95$ $\alpha_{ℛ}=0.95$	$\alpha_{𝒮}=0.9$ $\alpha_{𝒱}=0.9$ $\alpha_{ℐ}=0.9$ $\alpha_{ℛ}=0.9$	$\alpha_{𝒮}=0.85$ $\alpha_{𝒱}=0.85$ $\alpha_{ℐ}=0.85$ $\alpha_{ℛ}=0.85$	$\alpha_{𝒮}=0.8$ $\alpha_{𝒱}=0.8$ $\alpha_{ℐ}=0.8$ $\alpha_{ℛ}=0.8$	$\alpha_{𝒮}=0.75$ $\alpha_{𝒱}=0.75$ $\alpha_{ℐ}=0.75$ $\alpha_{ℛ}=0.75$	$\alpha_{𝒮}=0.7$ $\alpha_{𝒱}=0.7$ $\alpha_{ℐ}=0.7$ $\alpha_{ℛ}=0.7$	$\alpha_{𝒮}=0.65$ $\alpha_{𝒱}=0.65$ $\alpha_{ℐ}=0.65$ $\alpha_{ℛ}=0.65$	$\alpha_{𝒮}=0.6$ $\alpha_{𝒱}=0.6$ $\alpha_{ℐ}=0.6$ $\alpha_{ℛ}=0.6$
0.01	499.929	499.909	499.884	499.851	499.809	499.757	499.691	499.607	499.502
0.03	499.788	499.742	499.687	499.621	499.542	499.447	499.334	499.200	499.041
0.05	499.647	499.582	499.505	499.416	499.312	499.190	499.050	498.887	498.700
0.07	499.506	499.425	499.331	499.223	499.100	498.960	498.800	498.618	498.413
0.10	499.295	499.194	499.079	498.950	498.805	498.643	498.463	498.262	498.039
0.50	496.524	496.335	496.142	495.946	495.749	495.552	495.354	495.159	494.966
1.00	493.166	493.043	492.930	492.828	492.738	492.660	492.596	492.546	492.510
5.00	470.075	471.769	473.402	474.974	476.487	477.940	479.336	480.674	481.957
10.00	448.409	452.470	456.274	459.836	463.169	466.284	469.194	471.909	474.439
20.00	414.199	423.198	431.143	438.225	444.590	450.343	455.566	460.321	464.655

**Table 3:** Numerical values of the vaccinated population 𝒱(t) for homogeneous fractional orders.

*t*	$\alpha_{𝒮}=1$ $\alpha_{𝒱}=1$ $\alpha_{ℐ}=1$ $\alpha_{ℛ}=1$	$\alpha_{𝒮}=0.95$ $\alpha_{𝒱}=0.95$ $\alpha_{ℐ}=0.95$ $\alpha_{ℛ}=0.95$	$\alpha_{𝒮}=0.9$ $\alpha_{𝒱}=0.9$ $\alpha_{ℐ}=0.9$ $\alpha_{ℛ}=0.9$	$\alpha_{𝒮}=0.85$ $\alpha_{𝒱}=0.85$ $\alpha_{ℐ}=0.85$ $\alpha_{ℛ}=0.85$	$\alpha_{𝒮}=0.8$ $\alpha_{𝒱}=0.8$ $\alpha_{ℐ}=0.8$ $\alpha_{ℛ}=0.8$	$\alpha_{𝒮}=0.75$ $\alpha_{𝒱}=0.75$ $\alpha_{ℐ}=0.75$ $\alpha_{ℛ}=0.75$	$\alpha_{𝒮}=0.7$ $\alpha_{𝒱}=0.7$ $\alpha_{ℐ}=0.7$ $\alpha_{ℛ}=0.7$	$\alpha_{𝒮}=0.65$ $\alpha_{𝒱}=0.65$ $\alpha_{ℐ}=0.65$ $\alpha_{ℛ}=0.65$	$\alpha_{𝒮}=0.6$ $\alpha_{𝒱}=0.6$ $\alpha_{ℐ}=0.6$ $\alpha_{ℛ}=0.6$
0.01	350.000	349.999	349.999	349.999	349.999	349.998	349.998	349.998	349.997
0.03	349.999	349.998	349.998	349.998	349.997	349.997	349.996	349.995	349.994
0.05	349.998	349.997	349.997	349.996	349.996	349.995	349.994	349.993	349.992
0.07	349.997	349.996	349.996	349.995	349.994	349.993	349.992	349.991	349.990
0.10	349.996	349.995	349.994	349.993	349.992	349.991	349.990	349.989	349.987
0.50	349.977	349.975	349.974	349.972	349.970	349.968	349.967	349.965	349.963
1.00	349.950	349.949	349.947	349.946	349.945	349.943	349.942	349.941	349.940
5.00	349.640	349.663	349.685	349.706	349.727	349.747	349.766	349.784	349.801
10.00	349.075	349.176	349.267	349.350	349.424	349.492	349.552	349.607	349.656
20.00	347.807	348.107	348.374	348.609	348.816	348.996	349.152	349.287	349.402

**Table 4:** Numerical values of the infected population ℐ(t) for homogeneous fractional orders.

*t*	$\alpha_{𝒮}=1$ $\alpha_{𝒱}=1$ $\alpha_{ℐ}=1$ $\alpha_{ℛ}=1$	$\alpha_{𝒮}=0.95$ $\alpha_{𝒱}=0.95$ $\alpha_{ℐ}=0.95$ $\alpha_{ℛ}=0.95$	$\alpha_{𝒮}=0.9$ $\alpha_{𝒱}=0.9$ $\alpha_{ℐ}=0.9$ $\alpha_{ℛ}=0.9$	$\alpha_{𝒮}=0.85$ $\alpha_{𝒱}=0.85$ $\alpha_{ℐ}=0.85$ $\alpha_{ℛ}=0.85$	$\alpha_{𝒮}=0.8$ $\alpha_{𝒱}=0.8$ $\alpha_{ℐ}=0.8$ $\alpha_{ℛ}=0.8$	$\alpha_{𝒮}=0.75$ $\alpha_{𝒱}=0.75$ $\alpha_{ℐ}=0.75$ $\alpha_{ℛ}=0.75$	$\alpha_{𝒮}=0.7$ $\alpha_{𝒱}=0.7$ $\alpha_{ℐ}=0.7$ $\alpha_{ℛ}=0.7$	$\alpha_{𝒮}=0.65$ $\alpha_{𝒱}=0.65$ $\alpha_{ℐ}=0.65$ $\alpha_{ℛ}=0.65$	$\alpha_{𝒮}=0.6$ $\alpha_{𝒱}=0.6$ $\alpha_{ℐ}=0.6$ $\alpha_{ℛ}=0.6$
0.01	149.926	149.906	149.879	149.845	149.802	149.747	149.678	149.591	149.482
0.03	149.779	149.732	149.674	149.606	149.523	149.424	149.306	149.166	148.999
0.05	149.633	149.565	149.485	149.391	149.283	149.156	149.009	148.839	148.642
0.07	149.486	149.401	149.303	149.190	149.062	148.914	148.747	148.556	148.340
0.10	149.266	149.160	149.040	148.905	148.753	148.583	148.393	148.182	147.947
0.50	146.354	146.152	145.945	145.736	145.523	145.309	145.095	144.881	144.670
1.00	142.773	142.634	142.505	142.388	142.282	142.190	142.111	142.048	142.000
5.00	116.517	118.431	120.278	122.060	123.776	125.427	127.014	128.536	129.996
10.00	90.085	94.867	99.369	103.598	107.563	111.274	114.739	117.968	120.971
20.00	61.366	67.307	73.613	80.033	86.384	92.534	98.393	103.904	109.031

**Table 5:** Numerical values of the recovered population ℛ(t) for homogeneous fractional orders.

*t*	$\alpha_{𝒮}=1$ $\alpha_{𝒱}=1$ $\alpha_{ℐ}=1$ $\alpha_{ℛ}=1$	$\alpha_{𝒮}=0.95$ $\alpha_{𝒱}=0.95$ $\alpha_{ℐ}=0.95$ $\alpha_{ℛ}=0.95$	$\alpha_{𝒮}=0.9$ $\alpha_{𝒱}=0.9$ $\alpha_{ℐ}=0.9$ $\alpha_{ℛ}=0.9$	$\alpha_{𝒮}=0.85$ $\alpha_{𝒱}=0.85$ $\alpha_{ℐ}=0.85$ $\alpha_{ℛ}=0.85$	$\alpha_{𝒮}=0.8$ $\alpha_{𝒱}=0.8$ $\alpha_{ℐ}=0.8$ $\alpha_{ℛ}=0.8$	$\alpha_{𝒮}=0.75$ $\alpha_{𝒱}=0.75$ $\alpha_{ℐ}=0.75$ $\alpha_{ℛ}=0.75$	$\alpha_{𝒮}=0.7$ $\alpha_{𝒱}=0.7$ $\alpha_{ℐ}=0.7$ $\alpha_{ℛ}=0.7$	$\alpha_{𝒮}=0.65$ $\alpha_{𝒱}=0.65$ $\alpha_{ℐ}=0.65$ $\alpha_{ℛ}=0.65$	$\alpha_{𝒮}=0.6$ $\alpha_{𝒱}=0.6$ $\alpha_{ℐ}=0.6$ $\alpha_{ℛ}=0.6$
0.01	50.148	50.191	50.245	50.313	50.400	50.510	50.650	50.825	51.046
0.03	50.445	50.541	50.657	50.796	50.963	51.162	51.399	51.682	52.017
0.05	50.742	50.879	51.040	51.228	51.447	51.702	51.998	52.341	52.736
0.07	51.038	51.209	51.406	51.633	51.892	52.189	52.526	52.909	53.343
0.10	51.481	51.695	51.937	52.209	52.514	52.856	53.237	53.662	54.133
0.50	57.334	57.738	58.150	58.568	58.990	59.415	59.840	60.263	60.680
1.00	64.489	64.760	65.010	65.238	65.441	65.618	65.767	65.885	65.972
5.00	115.660	111.918	108.309	104.831	101.483	98.263	95.170	92.202	89.358
10.00	166.222	156.935	148.220	140.053	132.410	125.268	118.603	112.395	106.621
20.00	234.230	218.064	202.724	188.255	174.687	162.031	150.286	139.440	129.471

The numerical comparison between the LADM and the Differential Transform Method (DTM) for the integer-order rotavirus SVIR model is presented in Table 6. The DTM results are computed following the procedure described in [16] using the same assumed parameter values. The numerical values demonstrate that the LADM and DTM solutions are in excellent agreement for smaller time intervals, confirming the accuracy and correctness of the proposed LADM formulation. However, as the time variable increases, slight deviations begin to appear in the DTM results, whereas the LADM solutions remain stable and consistent. This indicates the higher stability and reliability of LADM over larger time domains. In the absence of benchmark solutions for the fractional-order rotavirus SVIR model in the literature, the integer-order case is employed for validation through comparative analysis. Therefore, the close agreement between LADM and DTM in the integer-order model provides strong evidence for the validity and effectiveness of the proposed LADM approach for solving the fractional-order system.

**Table 6:** Numerical comparison of LADM and DTM solutions for the integer-order SVIR model.

*t*	𝒮(t)		𝒱(t)		ℐ(t)		ℛ(t)	
	LADM	DTM	LADM	DTM	LADM	DTM	LADM	DTM
0.01	499.92930	499.92930	349.99956	349.99956	149.92646	149.92646	50.14845	50.14845
0.03	499.78805	499.78805	349.99869	349.99869	149.77946	149.77946	50.44513	50.44513
0.05	499.64699	499.64700	349.99781	349.99780	149.63256	149.63256	50.74153	50.74153
0.07	499.50613	499.50613	349.99692	349.99691	149.48576	149.48576	51.03763	51.03763
0.10	499.29520	499.29521	349.99558	349.99557	149.26575	149.26575	51.48123	51.48123
0.50	496.52411	496.52412	349.97664	349.97657	146.35431	146.35430	57.33381	57.33381
1.00	493.16610	493.16620	349.95016	349.95003	142.77292	142.77282	64.48863	64.48865
5.00	470.07494	470.12214	349.64029	349.63811	116.51723	116.46161	115.65953	115.66956
10.00	448.40868	449.13488	349.07547	349.05117	90.08462	89.31782	166.22179	166.28549
20.00	414.19883	424.92099	347.80697	347.48693	61.36622	53.03690	234.22999	232.15560

**Fig 5:**
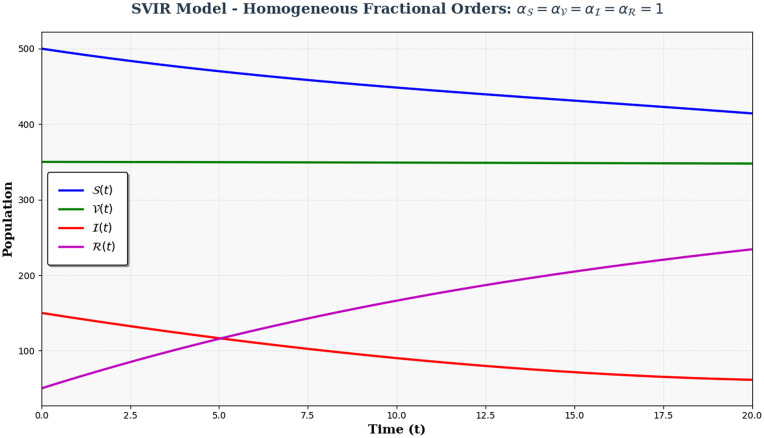
Time evolution of the susceptible 𝒮(t), vaccinated 𝒱(t), infected ℐ(t), and recovered ℛ(t) populations for homogeneous fractional orders $\alpha_{𝒮}=\alpha_{𝒱}=\alpha_{ℐ}=\alpha_{ℛ}=1$.

**Fig 6:**
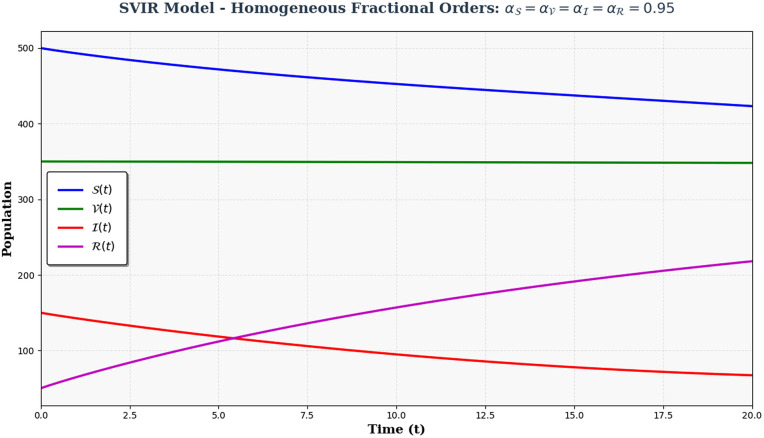
Time evolution of the susceptible 𝒮(t), vaccinated 𝒱(t), infected ℐ(t), and recovered ℛ(t) populations for homogeneous fractional orders $\alpha_{𝒮}=\alpha_{𝒱}=\alpha_{ℐ}=\alpha_{ℛ}=0.95$.

**Fig 7:**
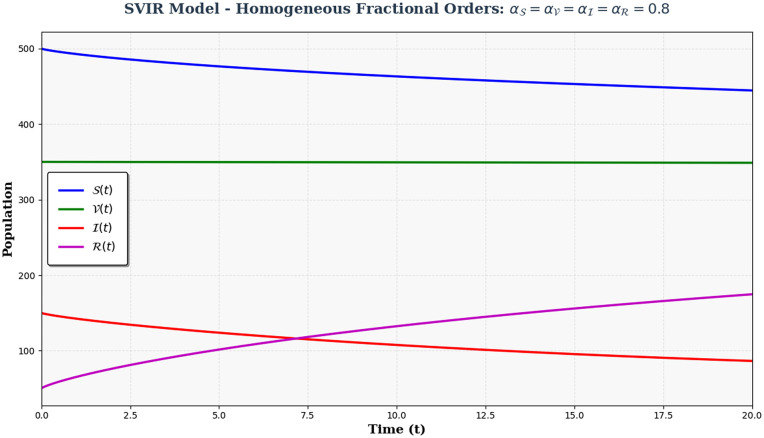
Time evolution of the susceptible 𝒮(t), vaccinated 𝒱(t), infected ℐ(t), and recovered ℛ(t) populations for homogeneous fractional orders $\alpha_{𝒮}=\alpha_{𝒱}=\alpha_{ℐ}=\alpha_{ℛ}=0.8$.

Figs 5–7 illustrate the time evolution of the susceptible 𝒮(t), vaccinated 𝒱(t), infected ℐ(t), and recovered ℛ(t) populations for different homogeneous fractional orders over the time interval 0≤t≤20. In Fig 5, corresponding to the classical case $\alpha_{S}=\alpha_{V}=\alpha_{I}=\alpha_{R}=1$, the susceptible population decreases gradually due to infection and vaccination effects, while the infected population also decreases steadily, indicating effective disease control. At the same time, the recovered population increases continuously as infected individuals recover, whereas the vaccinated population shows only slight variation and remains nearly stable. In Fig 6, for the fractional order 0.95, the dynamics begin to exhibit memory effects, where the decay of both susceptible and infected populations becomes slower compared to the classical case, and the growth of the recovered population is slightly delayed. In Fig 7, for the lower fractional order 0.8, these memory effects become more pronounced, leading to a further slowdown in the decrease of susceptible and infected populations, while the recovery process is also delayed. Overall, the results show that decreasing the fractional order intensifies memory effects in the system, which significantly influences the transmission and recovery dynamics of the rotavirus SVIR model.

**Fig 8:**
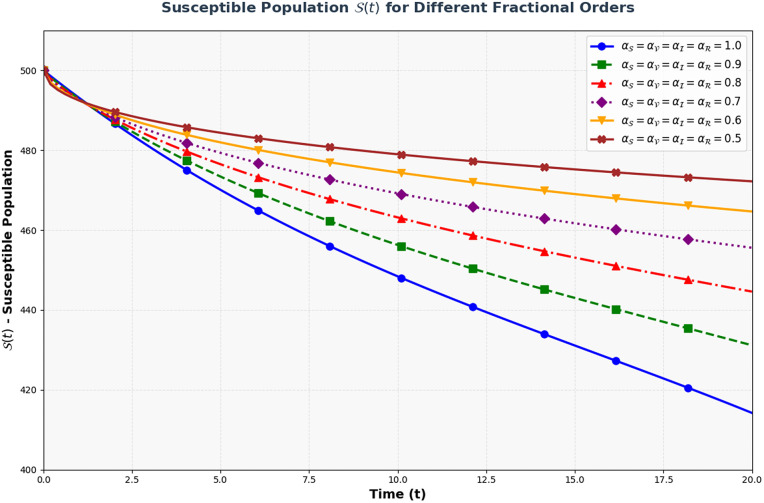
Variation of the susceptible population 𝒮(t) for different homogeneous fractional orders $\alpha_{𝒮}=\alpha_{𝒱}=\alpha_{ℐ}=\alpha_{ℛ}\in \{1.0,0.9,0.8,0.7,0.6,0.5\}$ over the time interval 0≤t≤20.

**Fig 9:**
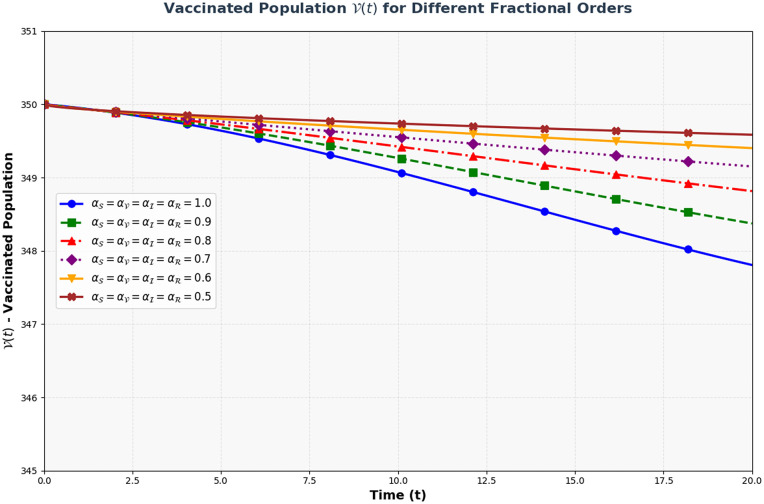
Variation of the Vaccinated population 𝒱(t) for different homogeneous fractional orders $\alpha_{𝒮}=\alpha_{𝒱}=\alpha_{ℐ}=\alpha_{ℛ}\in \{1.0,0.9,0.8,0.7,0.6,0.5\}$ over the time interval 0≤t≤20.

**Fig 10:**
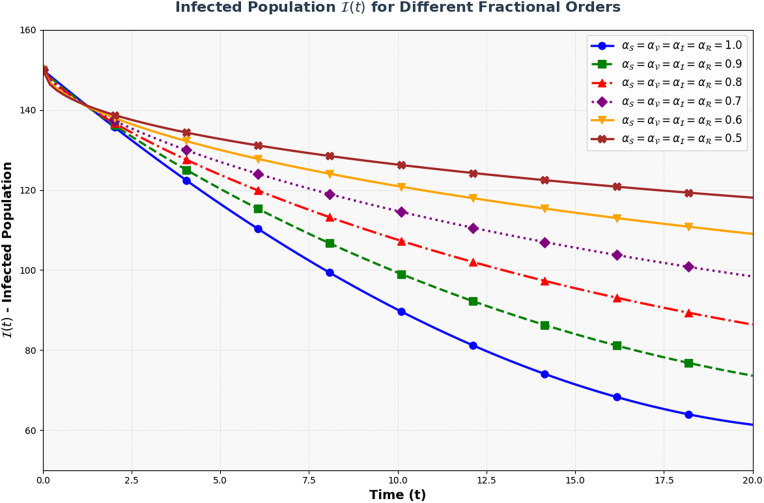
Variation of the Infected population ℐ(t) for different homogeneous fractional orders $\alpha_{𝒮}=\alpha_{𝒱}=\alpha_{ℐ}=\alpha_{ℛ}\in \{1.0,0.9,0.8,0.7,0.6,0.5\}$ over the time interval 0≤t≤20.

**Fig 11:**
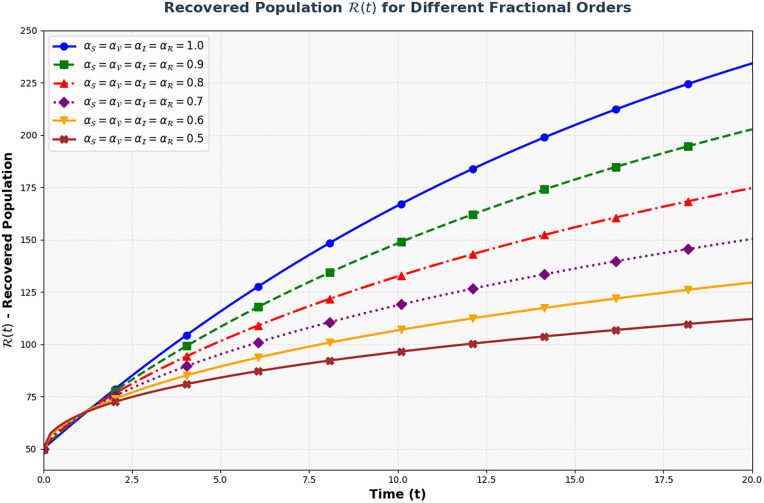
Variation of the recovered population ℛ(t) for different homogeneous fractional orders $\alpha_{𝒮}=\alpha_{𝒱}=\alpha_{ℐ}=\alpha_{ℛ}\in \{1.0,0.9,0.8,0.7,0.6,0.5\}$ over the time interval 0≤t≤20.

Figs 8–11 illustrate the time evolution of the susceptible 𝒮(t), vaccinated 𝒱(t), infected ℐ(t), and recovered ℛ(t) populations obtained from the LADM solutions for different homogeneous fractional orders (1.0,0.9,0.8,0.7,0.6,0.5) over the time interval 0≤t≤20. These figures are generated directly from the analytical LADM expressions which match with the corresponding numerical tables. In Fig 8, the susceptible population decreases over time, and lower fractional orders lead to a slower decay, indicating that memory effects delay the depletion of susceptible individuals. Fig 9 shows that the vaccinated population remains relatively stable for all fractional orders with only slight variations, although smaller fractional orders slightly modify its long-term behavior. In Fig 10, the infected population shows greater persistence for lower fractional orders, indicating that fractional memory effects reduce the rate of infection decay and extend the duration of disease presence in the population. Finally, Fig 11 shows that the recovered population increases over time for all cases but the rate of growth decreases as the fractional order decreases, reflecting the effect of memory on the recovery process.

**Fig 12:**
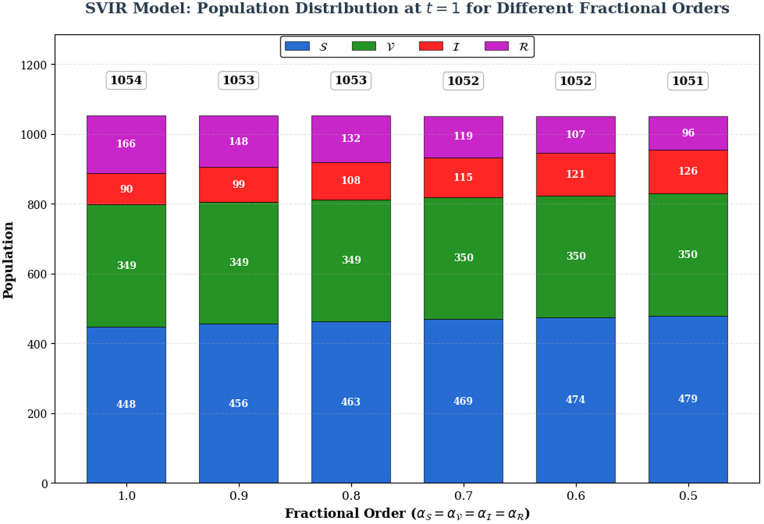
Population distribution of the SVIR compartments 𝒮,𝒱,ℐ,ℛ at *t* = 1 for different fractional orders $\alpha_{𝒮}=\alpha_{𝒱}=\alpha_{ℐ}=\alpha_{ℛ}\in \{1.0,0.9,0.8,0.7,0.6,0.5\}$.

**Fig 13:**
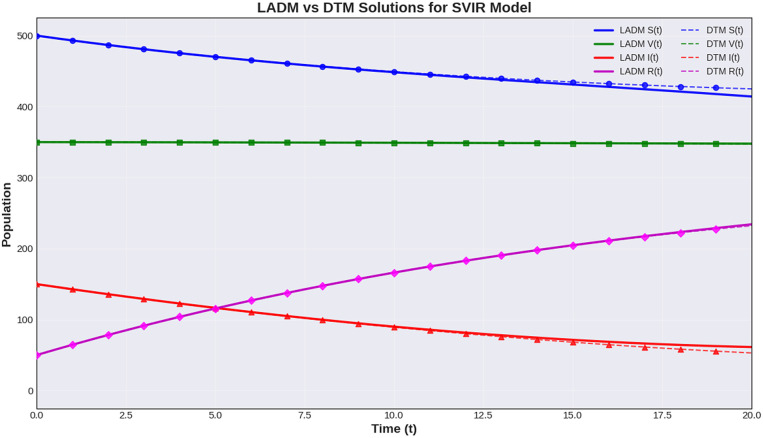
Comparison of LADM and DTM solutions for the susceptible 𝒮(t), vaccinated 𝒱(t), infected ℐ(t), and recovered ℛ(t) populations over 0≤t≤20.

Fig 12 presents the population distribution of the SVIR compartments at the fixed time instant *t* = 1 for different homogeneous fractional orders $\alpha_{𝒮}=\alpha_{𝒱}=\alpha_{ℐ}=\alpha_{ℛ}\in \{1.0,0.9,0.8,0.7,0.6,0.5\}$. It can be seen from the figure that as the fractional order decreases, the susceptible population 𝒮 decreases slightly and the infected population ℐ also decreases slightly for lower fractional orders. As the recovered population ℛ increases, showing the effect of memory on the recovery dynamics. For all fractional orders, the number of vaccinated individuals 𝒱 shows only a minor change during the considered simulation time (Fig 13).

In the graphical representation, the effect of past history on the present dynamics is clearly observed. From a physical and epidemiological perspective, the fractional-order parameter reflects memory and hereditary effects in disease transmission. Lower fractional orders indicate stronger memory effects, meaning that the present dynamics depend not only on the current state but also on the past history of the epidemic. This is very important in mathematical biological modeling, as such models need to consider past history to accurately describe real disease dynamics.

## 9 Conclusion

In this work, a Caputo fractional-order SVIR epidemic model for rotavirus transmission was proposed to incorporate memory and hereditary effects in disease dynamics. The disease-free and endemic equilibrium points of the model were determined, and the vaccination reproduction number $R_{v}$ was derived. The stability analysis of the model showed that the disease-free equilibrium is locally and globally asymptotically stable. Sensitivity analysis revealed that the transmission and recruitment rates increase disease spread, whereas vaccination and recovery-related parameters contribute significantly to reducing the effective reproduction number. Positivity analysis confirms the biological feasibility of the model by ensuring that the population dynamics remain biologically meaningful. We successfully applied the Laplace Adomian Decomposition Method to the fractional-order SVIR model and obtained approximate solutions, which are presented both numerically and graphically for different fractional orders. The results show that fractional-order parameters strongly influence the disease dynamics, Furthermore, the validity of the solution is verified by comparing the LADM results with the Differential Transform Method in the classical-order case. Since fractional-order formulations are not available in DTM, the comparison is performed only for the integer-order system, and a close agreement between both methods is observed. Overall, the numerical and graphical results demonstrate that the fractional-order framework captures the nonlocal and long-term behavior of rotavirus transmission more effectively than the classical integer–order approach, and therefore the proposed fractional SVIR model combined with the LADM technique provides an efficient mathematical framework for analyzing rotavirus dynamics and evaluating vaccination strategies. Although the proposed fractional-order SVIR model is developed under simplified assumptions with constant parameters and four compartments, real-world situations involve additional complexities. Factors such as seasonal variations, changing vaccination rates, environmental influences, and population heterogeneity may significantly affect disease transmission. Therefore, extending the present model by incorporating time-dependent parameters, stochastic effects, or spatial variations could provide a more realistic description of rotavirus dynamics and may be considered in future studies.

## Supporting information

S1 Data Data_for_Plos_One. Python code used for the numerical simulation of the fractional SVIR model.
(PDF)
