# Supplementary material for: Caputo fractional-order SVIR model for rotavirus: Numerical solutions using Laplace-Adomian decomposition method
Source: PLoS One. 2026 Jul 21;21(7):e0353071. doi: 10.1371/journal.pone.0353071 (PMC13387580; doi:10.1371/journal.pone.0353071)
Supplement: S1 Data — Python code used for the numerical simulation of the fractional SVIR model. (PDF) [file pone.0353071.s001.pdf]

Double-click (or enter) to edit

Numerical data

```
import numpy as np
from scipy.special import gamma


def gamma_ratio(x):
    return gamma(1 + x)

def susceptible(t, alpha, beta, eta, rho):

    S0 = 500 + (0.410126 * t**alpha) / gamma_ratio(alpha)


    S1 = -((7.4824 * t**alpha) / gamma_ratio(alpha)) \
         - (0.00693498 * t**(2 * alpha)) / gamma_ratio(2 * alpha) \
         + (2.15056e-6 * t**(alpha + beta)) / gamma_ratio(alpha + beta)


    S2 = (0.126523 * t**(2 * alpha)) / gamma_ratio(2 * alpha) \
         + (0.000117266 * t**(3 * alpha)) / gamma_ratio(3 * alpha) \
         - (0.000123065 * t**(alpha + beta)) / gamma_ratio(alpha + beta) \
         + (2.11013e-6 * t**(2 * alpha + beta)) / gamma_ratio(2 * alpha + beta) \
         - (6.06029e-9 * t**(alpha + 2 * beta)) / gamma_ratio(alpha + 2 * beta) \
         + (0.367763 * t**(alpha + eta)) / gamma_ratio(alpha + eta) \
         - (0.000307595 * t**(2 * alpha + eta)) / gamma_ratio(2 * alpha + eta) \
         + (0.000301658 * t**(2 * alpha + eta) * gamma_ratio(alpha + eta)) / \
           (gamma_ratio(alpha) * gamma_ratio(eta) * gamma_ratio(2 * alpha + eta)) \
         - (2.52305e-7 * t**(3 * alpha + eta) * gamma_ratio(2 * alpha + eta)) / \
           (gamma_ratio(alpha) * gamma_ratio(alpha + eta) * gamma_ratio(3 * alpha + eta)) \
         - (5.80605e-10 * t**(alpha + beta + eta)) / gamma_ratio(alpha + beta + eta) \
         - (4.76242e-13 * t**(2 * alpha + beta + eta) * gamma_ratio(alpha + beta + eta)) / \
           (gamma_ratio(alpha) * gamma_ratio(beta + eta) * gamma_ratio(2 * alpha + beta + eta))


    S3 = -((0.00213943 * t**(3 * alpha)) / gamma_ratio(3 * alpha)) \
         - (1.9829e-6 * t**(4 * alpha)) / gamma_ratio(4 * alpha) \
         - (0.00003708 * t**(2 * alpha + beta)) / gamma_ratio(2 * alpha + beta) \
         - (7.1977e-8 * t**(3 * alpha + beta)) / gamma_ratio(3 * alpha + beta) \
         + (3.46797e-7 * t**(alpha + 2 * beta)) / gamma_ratio(alpha + 2 * beta) \
         - (5.93509e-9 * t**(2 * alpha + 2 * beta)) / gamma_ratio(2 * alpha + 2 * beta) \
         + (1.70779e-11 * t**(alpha + 3 * beta)) / gamma_ratio(alpha + 3 * beta) \
         - (0.000606852 * t**(2 * alpha + eta)) / gamma_ratio(2 * alpha + eta) \
         - (0.00550349 * t**(2 * alpha + eta) * gamma_ratio(alpha + eta)) / \
           (gamma_ratio(alpha) * gamma_ratio(eta) * gamma_ratio(2 * alpha + eta)) \
         + (0.0000104025 * t**(3 * alpha + eta)) / gamma_ratio(3 * alpha + eta) \
         - (5.10086e-6 * t**(3 * alpha + eta) * gamma_ratio(alpha + eta)) / \
           (gamma_ratio(alpha) * gamma_ratio(eta) * gamma_ratio(3 * alpha + eta)) \
         - (5.10085e-6 * t**(3 * alpha + eta) * gamma_ratio(2 * alpha + eta)) / \
           (gamma_ratio(2 * alpha) * gamma_ratio(eta) * gamma_ratio(3 * alpha + eta)) \
         + (9.20618e-6 * t**(3 * alpha + eta) * gamma_ratio(2 * alpha + eta)) / \
           (gamma_ratio(alpha) * gamma_ratio(alpha + eta) * gamma_ratio(3 * alpha + eta)) \
         + (4.26633e-9 * t**(4 * alpha + eta) * gamma_ratio(2 * alpha + eta)) / \
           (gamma_ratio(alpha) * gamma_ratio(alpha + eta) * gamma_ratio(4 * alpha + eta)) \
         + (4.26632e-9 * t**(4 * alpha + eta) * gamma_ratio(3 * alpha + eta)) / \
           (gamma_ratio(2 * alpha) * gamma_ratio(alpha + eta) * gamma_ratio(4 * alpha + eta)) \
         + (4.26634e-9 * t**(4 * alpha + eta) * gamma_ratio(3 * alpha + eta)) / \
           (gamma_ratio(alpha) * gamma_ratio(2 * alpha + eta) * gamma_ratio(4 * alpha + eta)) \
         + (7.48377e-7 * t**(alpha + beta + eta)) / gamma_ratio(alpha + beta + eta) \
         - (2.78076e-9 * t**(2 * alpha + beta + eta)) / gamma_ratio(2 * alpha + beta + eta) \
         + (1.58179e-9 * t**(2 * alpha + beta + eta) * gamma_ratio(alpha + beta + eta)) / \
           (gamma_ratio(alpha + beta) * gamma_ratio(eta) * gamma_ratio(2 * alpha + beta + eta)) \
         + (3.59415e-11 * t**(2 * alpha + beta + eta) * gamma_ratio(alpha + beta + eta)) / \
           (gamma_ratio(alpha) * gamma_ratio(beta + eta) * gamma_ratio(2 * alpha + beta + eta)) \
         + (8.05297e-15 * t**(3 * alpha + beta + eta) * gamma_ratio(alpha + beta + eta)) / \
           (gamma_ratio(alpha) * gamma_ratio(beta + eta) * gamma_ratio(3 * alpha + beta + eta)) \
         - (1.323e-12 * t**(3 * alpha + beta + eta) * gamma_ratio(2 * alpha + beta + eta)) / \
           (gamma_ratio(alpha + beta) * gamma_ratio(alpha + eta) * gamma_ratio(3 * alpha + beta + eta)) \
         + (8.05297e-15 * t**(3 * alpha + beta + eta) * gamma_ratio(2 * alpha + beta + eta)) / \
           (gamma_ratio(2 * alpha) * gamma_ratio(beta + eta) * gamma_ratio(3 * alpha + beta + eta)) \
         - (1.79835e-12 * t**(3 * alpha + beta + eta) * gamma_ratio(2 * alpha + beta + eta)) / \
           (gamma_ratio(alpha) * gamma_ratio(alpha + beta + eta) * gamma_ratio(3 * alpha + beta + eta)) \
```

```
[illegible]
        + (1.63502e-12 * t**(alpha + 2 * beta + eta)) / gamma_ratio(alpha + 2 * beta + eta) \
        + (1.58179e-12 * t**(alpha + 2 * beta + eta) * gamma_ratio(beta + eta)) / \
          (gamma_ratio(beta) * gamma_ratio(eta) * gamma_ratio(alpha + 2 * beta + eta)) \
        - (1.323e-15 * t**(2 * alpha + 2 * beta + eta) * gamma_ratio(alpha + beta + eta)) / \
          (gamma_ratio(beta) * gamma_ratio(alpha + eta) * gamma_ratio(2 * alpha + 2 * beta + eta)) \
        - (2.49725e-18 * t**(2 * alpha + 2 * beta + eta) * gamma_ratio(alpha + 2 * beta + eta)) / \
          (gamma_ratio(alpha + beta) * gamma_ratio(beta + eta) * gamma_ratio(2 * alpha + 2 * beta + eta)) \
        + (1.34205e-15 * t**(2 * alpha + 2 * beta + eta) * gamma_ratio(alpha + 2 * beta + eta)) / \
          (gamma_ratio(alpha) * gamma_ratio(2 * beta + eta) * gamma_ratio(2 * alpha + 2 * beta + eta)) \
        - (2.49725e-21 * t**(alpha + 3 * beta + eta) * gamma_ratio(2 * beta + eta)) / \
          (gamma_ratio(beta) * gamma_ratio(beta + eta) * gamma_ratio(alpha + 3 * beta + eta)) \
        - (0.0180333 * t**(alpha + 2 * eta)) / gamma_ratio(alpha + 2 * eta) \
        + (0.0000150829 * t**(2 * alpha + 2 * eta)) / gamma_ratio(2 * alpha + 2 * eta) \
        + (0.0000150829 * t**(2 * alpha + 2 * eta) * gamma_ratio(alpha + eta)) / \
          (gamma_ratio(alpha) * gamma_ratio(eta) * gamma_ratio(2 * alpha + 2 * eta)) \
        - (0.0000147918 * t**(2 * alpha + 2 * eta) * gamma_ratio(alpha + 2 * eta)) / \
          (gamma_ratio(alpha) * gamma_ratio(2 * eta) * gamma_ratio(2 * alpha + 2 * eta)) \
        - (1.26153e-8 * t**(3 * alpha + 2 * eta) * gamma_ratio(2 * alpha + eta)) / \
          (gamma_ratio(alpha) * gamma_ratio(alpha + eta) * gamma_ratio(3 * alpha + 2 * eta)) \
        + (1.23718e-8 * t**(3 * alpha + 2 * eta) * gamma_ratio(2 * alpha + 2 * eta)) / \
          (gamma_ratio(alpha) * gamma_ratio(alpha + 2 * eta) * gamma_ratio(3 * alpha + 2 * eta)) \
        + (1.23718e-8 * t**(3 * alpha + 2 * eta) * gamma_ratio(alpha + eta) * gamma_ratio(2 * alpha + 2 * eta)) / \
          (gamma_ratio(alpha)**2 * gamma_ratio(eta) * gamma_ratio(alpha + 2 * eta) * gamma_ratio(3 * alpha + 2 * eta)) \
        - (1.03477e-11 * t**(4 * alpha + 2 * eta) * gamma_ratio(2 * alpha + eta) * gamma_ratio(3 * alpha + 2 * eta)) / \
          (gamma_ratio(alpha)**2 * gamma_ratio(alpha + eta) * gamma_ratio(2 * alpha + 2 * eta) * gamma_ratio(4 * alpha + 2
        + (2.847e-11 * t**(alpha + beta + 2 * eta)) / gamma_ratio(alpha + beta + 2 * eta) \
        + (2.847e-11 * t**(alpha + beta + 2 * eta) * gamma_ratio(beta + eta)) / \
          (gamma_ratio(beta) * gamma_ratio(eta) * gamma_ratio(alpha + beta + 2 * eta)) \
        - (2.38121e-14 * t**(2 * alpha + beta + 2 * eta) * gamma_ratio(alpha + beta + eta)) / \
          (gamma_ratio(beta) * gamma_ratio(alpha + eta) * gamma_ratio(2 * alpha + beta + 2 * eta)) \
        - (2.38121e-14 * t**(2 * alpha + beta + 2 * eta) * gamma_ratio(alpha + beta + eta)) / \
          (gamma_ratio(alpha) * gamma_ratio(beta + eta) * gamma_ratio(2 * alpha + beta + 2 * eta)) \
        + (2.33525e-14 * t**(2 * alpha + beta + 2 * eta) * gamma_ratio(alpha + beta + 2 * eta)) / \
          (gamma_ratio(alpha) * gamma_ratio(beta + 2 * eta) * gamma_ratio(2 * alpha + beta + 2 * eta)) \
        + (2.33525e-14 * t**(2 * alpha + beta + 2 * eta) * gamma_ratio(beta + eta) * gamma_ratio(alpha + beta + 2 * eta))
          (gamma_ratio(alpha) * gamma_ratio(beta) * gamma_ratio(eta) * gamma_ratio(beta + 2 * eta) * gamma_ratio(2 * alpha
        - (1.95319e-17 * t**(3 * alpha + beta + 2 * eta) * gamma_ratio(alpha + beta + eta) * gamma_ratio(2 * alpha + beta
          (gamma_ratio(alpha) * gamma_ratio(beta) * gamma_ratio(alpha + eta) * gamma_ratio(alpha + beta + 2 * eta) * gamma
        - (1.95319e-17 * t**(3 * alpha + beta + 2 * eta) * gamma_ratio(alpha + beta + eta) * gamma_ratio(2 * alpha + beta
          (gamma_ratio(alpha)**2 * gamma_ratio(beta + eta) * gamma_ratio(alpha + beta + 2 * eta) * gamma_ratio(3 * alpha +
        - (4.4947e-20 * t**(alpha + 2 * beta + 2 * eta) * gamma_ratio(2 * beta + eta)) / \
          (gamma_ratio(beta) * gamma_ratio(beta + eta) * gamma_ratio(alpha + 2 * beta + 2 * eta)) \
        - (3.68678e-23 * t**(2 * alpha + 2 * beta + 2 * eta) * gamma_ratio(2 * beta + eta) * gamma_ratio(alpha + 2 * beta
          (gamma_ratio(alpha) * gamma_ratio(beta) * gamma_ratio(beta + eta) * gamma_ratio(2 * beta + 2 * eta) * gamma_rati

    return S0 + S1 + S2 + S3


def vaccinated(t, alpha, beta, eta, rho):

    V0 = 350 + (0.00077414 * t**beta) / gamma_ratio(beta)


    V1 = -((0.0443 * t**beta) / gamma_ratio(beta)) \
         + (0.000772677 * t**(alpha + beta)) / gamma_ratio(alpha + beta) \
         - (2.18153e-6 * t**(2 * beta)) / gamma_ratio(2 * beta)


    V2 = -((0.0140968 * t**(alpha + beta)) / gamma_ratio(alpha + beta)) \
         - (0.0000130655 * t**(2 * alpha + beta)) / gamma_ratio(2 * alpha + beta) \
         + (0.000124837 * t**(2 * beta)) / gamma_ratio(2 * beta) \
         - (2.17335e-6 * t**(alpha + 2 * beta)) / gamma_ratio(alpha + 2 * beta) \
         + (6.14755e-9 * t**(3 * beta)) / gamma_ratio(3 * beta) \
         + (0.000257434 * t**(beta + eta)) / gamma_ratio(beta + eta) \
         - (2.15316e-7 * t**(alpha + beta + eta)) / gamma_ratio(alpha + beta + eta) \
         - (4.06423e-13 * t**(2 * beta + eta)) / gamma_ratio(2 * beta + eta) \
         + (5.69399e-10 * t**(2 * beta + eta) * gamma_ratio(beta + eta)) / \
           (gamma_ratio(beta) * gamma_ratio(eta) * gamma_ratio(2 * beta + eta)) \
         - (4.76242e-13 * t**(alpha + 2 * beta + eta) * gamma_ratio(alpha + beta + eta)) / \
           (gamma_ratio(beta) * gamma_ratio(alpha + eta) * gamma_ratio(alpha + 2 * beta + eta)) \
         - (8.98939e-19 * t**(3 * beta + eta) * gamma_ratio(2 * beta + eta)) / \
           (gamma_ratio(beta) * gamma_ratio(beta + eta) * gamma_ratio(3 * beta + eta))

    V3 = (0.000238369 * t**(2 * alpha + beta)) / gamma_ratio(2 * alpha + beta) \
         + (2.20929e-7 * t**(3 * alpha + beta)) / gamma_ratio(3 * alpha + beta) \
         + (0.0000394929 * t**(alpha + 2 * beta)) / gamma_ratio(alpha + 2 * beta) \
         + (4.07941e-8 * t**(2 * alpha + 2 * beta)) / gamma_ratio(2 * alpha + 2 * beta) \
```

```
    - (3.51791e-7 * t**(3 * beta)) / gamma_ratio(3 * beta) \
    + (6.11308e-9 * t**(alpha + 3 * beta)) / gamma_ratio(alpha + 3 * beta) \
    - (1.73238e-11 * t**(4 * beta)) / gamma_ratio(4 * beta) \
    + (0.000696794 * t**(alpha + beta + eta)) / gamma_ratio(alpha + beta + eta) \
    - (5.75868e-7 * t**(2 * alpha + beta + eta)) / gamma_ratio(2 * alpha + beta + eta) \
    + (5.68324e-7 * t**(2 * alpha + beta + eta) * gamma_ratio(alpha + eta)) / \
      (gamma_ratio(alpha) * gamma_ratio(eta) * gamma_ratio(2 * alpha + beta + eta)) \
    - (4.75343e-10 * t**(3 * alpha + beta + eta) * gamma_ratio(2 * alpha + eta)) / \
      (gamma_ratio(alpha) * gamma_ratio(alpha + eta) * gamma_ratio(3 * alpha + beta + eta)) \
    - (7.25426e-7 * t**(2 * beta + eta)) / gamma_ratio(2 * beta + eta) \
    - (3.25838e-8 * t**(2 * beta + eta) * gamma_ratio(beta + eta)) / \
      (gamma_ratio(beta) * gamma_ratio(eta) * gamma_ratio(2 * beta + eta)) \
    + (6.04132e-10 * t**(alpha + 2 * beta + eta)) / gamma_ratio(alpha + 2 * beta + eta) \
    + (5.68323e-10 * t**(alpha + 2 * beta + eta) * gamma_ratio(alpha + beta + eta)) / \
      (gamma_ratio(alpha + beta) * gamma_ratio(eta) * gamma_ratio(alpha + 2 * beta + eta)) \
    + (3.59415e-11 * t**(alpha + 2 * beta + eta) * gamma_ratio(alpha + beta + eta)) / \
      (gamma_ratio(beta) * gamma_ratio(alpha + eta) * gamma_ratio(alpha + 2 * beta + eta)) \
    - (8.9724e-16 * t**(2 * alpha + 2 * beta + eta) * gamma_ratio(alpha + beta + eta)) / \
      (gamma_ratio(alpha) * gamma_ratio(beta + eta) * gamma_ratio(2 * alpha + 2 * beta + eta)) \
    - (4.75342e-13 * t**(2 * alpha + 2 * beta + eta) * gamma_ratio(2 * alpha + beta + eta)) / \
      (gamma_ratio(alpha + beta) * gamma_ratio(alpha + eta) * gamma_ratio(2 * alpha + 2 * beta + eta)) \
    + (8.05299e-15 * t**(2 * alpha + 2 * beta + eta) * gamma_ratio(2 * alpha + beta + eta)) / \
      (gamma_ratio(beta) * gamma_ratio(2 * alpha + eta) * gamma_ratio(2 * alpha + 2 * beta + eta)) \
    + (2.2906e-15 * t**(3 * beta + eta)) / gamma_ratio(3 * beta + eta) \
    - (1.60457e-12 * t**(3 * beta + eta) * gamma_ratio(beta + eta)) / \
      (gamma_ratio(beta) * gamma_ratio(eta) * gamma_ratio(3 * beta + eta)) \
    - (1.60457e-12 * t**(3 * beta + eta) * gamma_ratio(2 * beta + eta)) / \
      (gamma_ratio(2 * beta) * gamma_ratio(eta) * gamma_ratio(3 * beta + eta)) \
    + (1.02883e-16 * t**(3 * beta + eta) * gamma_ratio(2 * beta + eta)) / \
      (gamma_ratio(beta) * gamma_ratio(beta + eta) * gamma_ratio(3 * beta + eta)) \
    + (1.34205e-15 * t**(alpha + 3 * beta + eta) * gamma_ratio(alpha + beta + eta)) / \
      (gamma_ratio(beta) * gamma_ratio(alpha + eta) * gamma_ratio(alpha + 3 * beta + eta)) \
    + (1.34205e-15 * t**(alpha + 3 * beta + eta) * gamma_ratio(alpha + 2 * beta + eta)) / \
      (gamma_ratio(2 * beta) * gamma_ratio(alpha + eta) * gamma_ratio(alpha + 3 * beta + eta)) \
    - (8.9724e-19 * t**(alpha + 3 * beta + eta) * gamma_ratio(alpha + 2 * beta + eta)) / \
      (gamma_ratio(alpha + beta) * gamma_ratio(beta + eta) * gamma_ratio(alpha + 3 * beta + eta)) \
    - (3.3945e-18 * t**(alpha + 3 * beta + eta) * gamma_ratio(alpha + 2 * beta + eta)) / \
      (gamma_ratio(beta) * gamma_ratio(alpha + beta + eta) * gamma_ratio(alpha + 3 * beta + eta)) \
    + (2.53321e-21 * t**(4 * beta + eta) * gamma_ratio(2 * beta + eta)) / \
      (gamma_ratio(beta) * gamma_ratio(beta + eta) * gamma_ratio(4 * beta + eta)) \
    + (2.53321e-21 * t**(4 * beta + eta) * gamma_ratio(3 * beta + eta)) / \
      (gamma_ratio(2 * beta) * gamma_ratio(beta + eta) * gamma_ratio(4 * beta + eta)) \
    + (2.53321e-21 * t**(4 * beta + eta) * gamma_ratio(3 * beta + eta)) / \
      (gamma_ratio(beta) * gamma_ratio(2 * beta + eta) * gamma_ratio(4 * beta + eta)) \
    - (0.0000126233 * t**(beta + 2 * eta)) / gamma_ratio(beta + 2 * eta) \
    + (1.0558e-8 * t**(alpha + beta + 2 * eta)) / gamma_ratio(alpha + beta + 2 * eta) \
    + (1.0558e-8 * t**(alpha + beta + 2 * eta) * gamma_ratio(alpha + eta)) / \
      (gamma_ratio(alpha) * gamma_ratio(eta) * gamma_ratio(alpha + beta + 2 * eta)) \
    - (8.83067e-12 * t**(2 * alpha + beta + 2 * eta) * gamma_ratio(2 * alpha + eta)) / \
      (gamma_ratio(alpha) * gamma_ratio(alpha + eta) * gamma_ratio(2 * alpha + beta + 2 * eta)) \
    + (1.9929e-14 * t**(2 * beta + 2 * eta)) / gamma_ratio(2 * beta + 2 * eta) \
    + (1.9929e-14 * t**(2 * beta + 2 * eta) * gamma_ratio(beta + eta)) / \
      (gamma_ratio(beta) * gamma_ratio(eta) * gamma_ratio(2 * beta + 2 * eta)) \
    - (2.79205e-11 * t**(2 * beta + 2 * eta) * gamma_ratio(beta + 2 * eta)) / \
      (gamma_ratio(beta) * gamma_ratio(2 * eta) * gamma_ratio(2 * beta + 2 * eta)) \
    - (1.66685e-17 * t**(alpha + 2 * beta + 2 * eta) * gamma_ratio(alpha + beta + eta)) / \
      (gamma_ratio(beta) * gamma_ratio(alpha + eta) * gamma_ratio(alpha + 2 * beta + 2 * eta)) \
    - (1.66685e-17 * t**(alpha + 2 * beta + 2 * eta) * gamma_ratio(alpha + beta + eta)) / \
      (gamma_ratio(alpha) * gamma_ratio(beta + eta) * gamma_ratio(alpha + 2 * beta + 2 * eta)) \
    + (2.33526e-14 * t**(alpha + 2 * beta + 2 * eta) * gamma_ratio(alpha + beta + 2 * eta)) / \
      (gamma_ratio(beta) * gamma_ratio(alpha + 2 * eta) * gamma_ratio(alpha + 2 * beta + 2 * eta)) \
    + (2.33526e-14 * t**(alpha + 2 * beta + 2 * eta) * gamma_ratio(alpha + eta) * gamma_ratio(alpha + beta + 2 * eta))
      (gamma_ratio(alpha) * gamma_ratio(beta) * gamma_ratio(eta) * gamma_ratio(alpha + 2 * eta) * gamma_ratio(alpha +
    - (1.95319e-17 * t**(2 * alpha + 2 * beta + 2 * eta) * gamma_ratio(2 * alpha + eta) * gamma_ratio(2 * alpha + beta
      (gamma_ratio(alpha) * gamma_ratio(beta) * gamma_ratio(alpha + eta) * gamma_ratio(2 * alpha + 2 * eta) * gamma_ra
    - (3.14629e-23 * t**(3 * beta + 2 * eta) * gamma_ratio(2 * beta + eta)) / \
      (gamma_ratio(beta) * gamma_ratio(beta + eta) * gamma_ratio(3 * beta + 2 * eta)) \
    + (4.40795e-20 * t**(3 * beta + 2 * eta) * gamma_ratio(2 * beta + 2 * eta)) / \
      (gamma_ratio(beta) * gamma_ratio(beta + 2 * eta) * gamma_ratio(3 * beta + 2 * eta)) \
    + (4.40795e-20 * t**(3 * beta + 2 * eta) * gamma_ratio(beta + eta) * gamma_ratio(2 * beta + 2 * eta)) / \
      (gamma_ratio(beta)**2 * gamma_ratio(eta) * gamma_ratio(beta + 2 * eta) * gamma_ratio(3 * beta + 2 * eta)) \
    - (3.68678e-23 * t**(alpha + 3 * beta + 2 * eta) * gamma_ratio(alpha + beta + eta) * gamma_ratio(alpha + 2 * beta
      (gamma_ratio(beta)**2 * gamma_ratio(alpha + eta) * gamma_ratio(alpha + beta + 2 * eta) * gamma_ratio(alpha + 3 *
    - (3.68678e-23 * t**(alpha + 3 * beta + 2 * eta) * gamma_ratio(alpha + beta + eta) * gamma_ratio(alpha + 2 * beta
      (gamma_ratio(alpha) * gamma_ratio(beta) * gamma_ratio(beta + eta) * gamma_ratio(alpha + beta + 2 * eta) * gamma_
    - (6.95905e-29 * t**(4 * beta + 2 * eta) * gamma_ratio(2 * beta + eta) * gamma_ratio(3 * beta + 2 * eta)) / \
      (gamma_ratio(beta)**2 * gamma_ratio(beta + eta) * gamma_ratio(2 * beta + 2 * eta) * gamma_ratio(4 * beta + 2 * e
```

```
    return V0 + V1 + V2 + V3


def infected(t, alpha, beta, eta, rho):

    I0 = 150


    I1 = -((7.35525 * t**eta) / gamma_ratio(eta)) \
         + (0.00615189 * t**(alpha + eta)) / gamma_ratio(alpha + eta) \
         + (1.16121e-8 * t**(beta + eta)) / gamma_ratio(beta + eta)


    I2 = -((0.112236 * t**(alpha + eta)) / gamma_ratio(alpha + eta)) \
         - (0.000104025 * t**(2 * alpha + eta)) / gamma_ratio(2 * alpha + eta) \
         - (6.645e-7 * t**(beta + eta)) / gamma_ratio(beta + eta) \
         + (4.38486e-8 * t**(alpha + beta + eta)) / gamma_ratio(alpha + beta + eta) \
         - (3.27229e-11 * t**(2 * beta + eta)) / gamma_ratio(2 * beta + eta) \
         + (0.360665 * t**(2 * eta)) / gamma_ratio(2 * eta) \
         - (0.000301658 * t**(alpha + 2 * eta)) / gamma_ratio(alpha + 2 * eta) \
         - (0.000301658 * t**(alpha + 2 * eta) * gamma_ratio(alpha + eta)) / \
           (gamma_ratio(alpha) * gamma_ratio(eta) * gamma_ratio(alpha + 2 * eta)) \
         + (2.52305e-7 * t**(2 * alpha + 2 * eta) * gamma_ratio(2 * alpha + eta)) / \
           (gamma_ratio(alpha) * gamma_ratio(alpha + eta) * gamma_ratio(2 * alpha + 2 * eta)) \
         - (5.69399e-10 * t**(beta + 2 * eta)) / gamma_ratio(beta + 2 * eta) \
         - (5.69399e-10 * t**(beta + 2 * eta) * gamma_ratio(beta + eta)) / \
           (gamma_ratio(beta) * gamma_ratio(eta) * gamma_ratio(beta + 2 * eta)) \
         + (4.76242e-13 * t**(alpha + beta + 2 * eta) * gamma_ratio(alpha + beta + eta)) / \
           (gamma_ratio(beta) * gamma_ratio(alpha + eta) * gamma_ratio(alpha + beta + 2 * eta)) \
         + (4.76242e-13 * t**(alpha + beta + 2 * eta) * gamma_ratio(alpha + beta + eta)) / \
           (gamma_ratio(alpha) * gamma_ratio(beta + eta) * gamma_ratio(alpha + beta + 2 * eta)) \
         + (8.98939e-19 * t**(2 * beta + 2 * eta) * gamma_ratio(2 * beta + eta)) / \
           (gamma_ratio(beta) * gamma_ratio(beta + eta) * gamma_ratio(2 * beta + 2 * eta))

    I3 = (0.00189785 * t**(2 * alpha + eta)) / gamma_ratio(2 * alpha + eta) \
         + (1.75899e-6 * t**(3 * alpha + eta)) / gamma_ratio(3 * alpha + eta) \
         - (2.05743e-6 * t**(alpha + beta + eta)) / gamma_ratio(alpha + beta + eta) \
         + (3.1456e-8 * t**(2 * alpha + beta + eta)) / gamma_ratio(2 * alpha + beta + eta) \
         + (1.87256e-9 * t**(2 * beta + eta)) / gamma_ratio(2 * beta + eta) \
         - (1.23505e-10 * t**(alpha + 2 * beta + eta)) / gamma_ratio(alpha + 2 * beta + eta) \
         + (9.22133e-14 * t**(3 * beta + eta)) / gamma_ratio(3 * beta + eta) \
         + (0.0110199 * t**(alpha + 2 * eta)) / gamma_ratio(alpha + 2 * eta) \
         + (0.00550349 * t**(alpha + 2 * eta) * gamma_ratio(alpha + eta)) / \
           (gamma_ratio(alpha) * gamma_ratio(eta) * gamma_ratio(alpha + 2 * eta)) \
         + (4.86941e-7 * t**(2 * alpha + 2 * eta)) / gamma_ratio(2 * alpha + 2 * eta) \
         + (4.52487e-6 * t**(2 * alpha + 2 * eta) * gamma_ratio(alpha + eta)) / \
           (gamma_ratio(alpha) * gamma_ratio(eta) * gamma_ratio(2 * alpha + 2 * eta)) \
         + (5.10085e-6 * t**(2 * alpha + 2 * eta) * gamma_ratio(2 * alpha + eta)) / \
           (gamma_ratio(2 * alpha) * gamma_ratio(eta) * gamma_ratio(2 * alpha + 2 * eta)) \
         - (9.20618e-6 * t**(2 * alpha + 2 * eta) * gamma_ratio(2 * alpha + eta)) / \
           (gamma_ratio(alpha) * gamma_ratio(alpha + eta) * gamma_ratio(2 * alpha + 2 * eta)) \
         - (3.78458e-9 * t**(3 * alpha + 2 * eta) * gamma_ratio(2 * alpha + eta)) / \
           (gamma_ratio(alpha) * gamma_ratio(alpha + eta) * gamma_ratio(3 * alpha + 2 * eta)) \
         - (4.26632e-9 * t**(3 * alpha + 2 * eta) * gamma_ratio(3 * alpha + eta)) / \
           (gamma_ratio(2 * alpha) * gamma_ratio(alpha + eta) * gamma_ratio(3 * alpha + 2 * eta)) \
         - (4.26634e-9 * t**(3 * alpha + 2 * eta) * gamma_ratio(3 * alpha + eta)) / \
           (gamma_ratio(alpha) * gamma_ratio(2 * alpha + eta) * gamma_ratio(3 * alpha + 2 * eta)) \
         + (3.64453e-8 * t**(beta + 2 * eta)) / gamma_ratio(beta + 2 * eta) \
         + (3.25838e-8 * t**(beta + 2 * eta) * gamma_ratio(beta + eta)) / \
           (gamma_ratio(beta) * gamma_ratio(eta) * gamma_ratio(beta + 2 * eta)) \
         - (2.16205e-9 * t**(alpha + beta + 2 * eta)) / gamma_ratio(alpha + beta + 2 * eta) \
         - (2.15011e-9 * t**(alpha + beta + 2 * eta) * gamma_ratio(alpha + beta + eta)) / \
           (gamma_ratio(alpha + beta) * gamma_ratio(eta) * gamma_ratio(alpha + beta + 2 * eta)) \
         - (3.59415e-11 * t**(alpha + beta + 2 * eta) * gamma_ratio(alpha + beta + eta)) / \
           (gamma_ratio(beta) * gamma_ratio(alpha + eta) * gamma_ratio(alpha + beta + 2 * eta)) \
         - (3.59415e-11 * t**(alpha + beta + 2 * eta) * gamma_ratio(alpha + beta + eta)) / \
           (gamma_ratio(alpha) * gamma_ratio(beta + eta) * gamma_ratio(alpha + beta + 2 * eta)) \
         - (7.14363e-15 * t**(2 * alpha + beta + 2 * eta) * gamma_ratio(alpha + beta + eta)) / \
           (gamma_ratio(alpha) * gamma_ratio(beta + eta) * gamma_ratio(2 * alpha + beta + 2 * eta)) \
         + (1.79834e-12 * t**(2 * alpha + beta + 2 * eta) * gamma_ratio(2 * alpha + beta + eta)) / \
           (gamma_ratio(alpha + beta) * gamma_ratio(alpha + eta) * gamma_ratio(2 * alpha + beta + 2 * eta)) \
         - (8.05299e-15 * t**(2 * alpha + beta + 2 * eta) * gamma_ratio(2 * alpha + beta + eta)) / \
           (gamma_ratio(beta) * gamma_ratio(2 * alpha + eta) * gamma_ratio(2 * alpha + beta + 2 * eta)) \
         - (8.05297e-15 * t**(2 * alpha + beta + 2 * eta) * gamma_ratio(2 * alpha + beta + eta)) / \
           (gamma_ratio(2 * alpha) * gamma_ratio(beta + eta) * gamma_ratio(2 * alpha + beta + 2 * eta)) \
         + (1.79835e-12 * t**(2 * alpha + beta + 2 * eta) * gamma_ratio(2 * alpha + beta + eta)) / \
           (gamma_ratio(alpha) * gamma_ratio(alpha + beta + eta) * gamma_ratio(2 * alpha + beta + 2 * eta)) \
```

```
        + (1.60456e-12 * t**(2 * beta + 2 * eta)) / gamma_ratio(2 * beta + 2 * eta) \
        + (8.54099e-15 * t**(2 * beta + 2 * eta) * gamma_ratio(beta + eta)) / \
          (gamma_ratio(beta) * gamma_ratio(eta) * gamma_ratio(2 * beta + 2 * eta)) \
        + (1.60457e-12 * t**(2 * beta + 2 * eta) * gamma_ratio(2 * beta + eta)) / \
          (gamma_ratio(2 * beta) * gamma_ratio(eta) * gamma_ratio(2 * beta + 2 * eta)) \
        - (1.02883e-16 * t**(2 * beta + 2 * eta) * gamma_ratio(2 * beta + eta)) / \
          (gamma_ratio(beta) * gamma_ratio(beta + eta) * gamma_ratio(2 * beta + 2 * eta)) \
        - (7.14363e-18 * t**(alpha + 2 * beta + 2 * eta) * gamma_ratio(alpha + beta + eta)) / \
          (gamma_ratio(beta) * gamma_ratio(alpha + eta) * gamma_ratio(alpha + 2 * beta + 2 * eta)) \
        - (1.34205e-15 * t**(alpha + 2 * beta + 2 * eta) * gamma_ratio(alpha + 2 * beta + eta)) / \
          (gamma_ratio(2 * beta) * gamma_ratio(alpha + eta) * gamma_ratio(alpha + 2 * beta + 2 * eta)) \
        + (3.39449e-18 * t**(alpha + 2 * beta + 2 * eta) * gamma_ratio(alpha + 2 * beta + eta)) / \
          (gamma_ratio(alpha + beta) * gamma_ratio(beta + eta) * gamma_ratio(alpha + 2 * beta + 2 * eta)) \
        + (3.3945e-18 * t**(alpha + 2 * beta + 2 * eta) * gamma_ratio(alpha + 2 * beta + eta)) / \
          (gamma_ratio(beta) * gamma_ratio(alpha + beta + eta) * gamma_ratio(alpha + 2 * beta + 2 * eta)) \
        - (1.34205e-15 * t**(alpha + 2 * beta + 2 * eta) * gamma_ratio(alpha + 2 * beta + eta)) / \
          (gamma_ratio(alpha) * gamma_ratio(2 * beta + eta) * gamma_ratio(alpha + 2 * beta + 2 * eta)) \
        - (1.34841e-23 * t**(3 * beta + 2 * eta) * gamma_ratio(2 * beta + eta)) / \
          (gamma_ratio(beta) * gamma_ratio(beta + eta) * gamma_ratio(3 * beta + 2 * eta)) \
        - (2.53321e-21 * t**(3 * beta + 2 * eta) * gamma_ratio(3 * beta + eta)) / \
          (gamma_ratio(2 * beta) * gamma_ratio(beta + eta) * gamma_ratio(3 * beta + 2 * eta)) \
        - (2.53321e-21 * t**(3 * beta + 2 * eta) * gamma_ratio(3 * beta + eta)) / \
          (gamma_ratio(beta) * gamma_ratio(2 * beta + eta) * gamma_ratio(3 * beta + 2 * eta)) \
        - (0.0176852 * t**(3 * eta)) / gamma_ratio(3 * eta) \
        + (0.0000147918 * t**(alpha + 3 * eta)) / gamma_ratio(alpha + 3 * eta) \
        + (0.0000147918 * t**(alpha + 3 * eta) * gamma_ratio(alpha + eta)) / \
          (gamma_ratio(alpha) * gamma_ratio(eta) * gamma_ratio(alpha + 3 * eta)) \
        + (0.0000147918 * t**(alpha + 3 * eta) * gamma_ratio(alpha + 2 * eta)) / \
          (gamma_ratio(alpha) * gamma_ratio(2 * eta) * gamma_ratio(alpha + 3 * eta)) \
        - (1.23718e-8 * t**(2 * alpha + 3 * eta) * gamma_ratio(2 * alpha + eta)) / \
          (gamma_ratio(alpha) * gamma_ratio(alpha + eta) * gamma_ratio(2 * alpha + 3 * eta)) \
        - (1.23718e-8 * t**(2 * alpha + 3 * eta) * gamma_ratio(2 * alpha + 2 * eta)) / \
          (gamma_ratio(alpha) * gamma_ratio(alpha + 2 * eta) * gamma_ratio(2 * alpha + 3 * eta)) \
        - (1.23718e-8 * t**(2 * alpha + 3 * eta) * gamma_ratio(alpha + eta) * gamma_ratio(2 * alpha + 2 * eta)) / \
          (gamma_ratio(alpha)**2 * gamma_ratio(eta) * gamma_ratio(alpha + 2 * eta) * gamma_ratio(2 * alpha + 3 * eta)) \
        + (1.03477e-11 * t**(3 * alpha + 3 * eta) * gamma_ratio(2 * alpha + eta) * gamma_ratio(3 * alpha + 2 * eta)) / \
          (gamma_ratio(alpha)**2 * gamma_ratio(alpha + eta) * gamma_ratio(2 * alpha + 2 * eta) * gamma_ratio(3 * alpha + 3
        + (2.79205e-11 * t**(beta + 3 * eta)) / gamma_ratio(beta + 3 * eta) \
        + (2.79205e-11 * t**(beta + 3 * eta) * gamma_ratio(beta + eta)) / \
          (gamma_ratio(beta) * gamma_ratio(eta) * gamma_ratio(beta + 3 * eta)) \
        + (2.79205e-11 * t**(beta + 3 * eta) * gamma_ratio(beta + 2 * eta)) / \
          (gamma_ratio(beta) * gamma_ratio(2 * eta) * gamma_ratio(beta + 3 * eta)) \
        - (2.33525e-14 * t**(alpha + beta + 3 * eta) * gamma_ratio(alpha + beta + eta)) / \
          (gamma_ratio(beta) * gamma_ratio(alpha + eta) * gamma_ratio(alpha + beta + 3 * eta)) \
        - (2.33525e-14 * t**(alpha + beta + 3 * eta) * gamma_ratio(alpha + beta + eta)) / \
          (gamma_ratio(alpha) * gamma_ratio(beta + eta) * gamma_ratio(alpha + beta + 3 * eta)) \
        - (2.33526e-14 * t**(alpha + beta + 3 * eta) * gamma_ratio(alpha + beta + 2 * eta)) / \
          (gamma_ratio(beta) * gamma_ratio(alpha + 2 * eta) * gamma_ratio(alpha + beta + 3 * eta)) \
        - (2.33526e-14 * t**(alpha + beta + 3 * eta) * gamma_ratio(alpha + eta) * gamma_ratio(alpha + beta + 2 * eta)) / \
          (gamma_ratio(alpha) * gamma_ratio(beta) * gamma_ratio(eta) * gamma_ratio(alpha + 2 * eta) * gamma_ratio(alpha +
        - (2.33525e-14 * t**(alpha + beta + 3 * eta) * gamma_ratio(alpha + beta + 2 * eta)) / \
          (gamma_ratio(alpha) * gamma_ratio(beta + 2 * eta) * gamma_ratio(alpha + beta + 3 * eta)) \
        - (2.33525e-14 * t**(alpha + beta + 3 * eta) * gamma_ratio(beta + eta) * gamma_ratio(alpha + beta + 2 * eta)) / \
          (gamma_ratio(alpha) * gamma_ratio(beta) * gamma_ratio(eta) * gamma_ratio(beta + 2 * eta) * gamma_ratio(alpha + b
        + (1.95319e-17 * t**(2 * alpha + beta + 3 * eta) * gamma_ratio(2 * alpha + eta) * gamma_ratio(2 * alpha + beta + 2
          (gamma_ratio(alpha) * gamma_ratio(beta) * gamma_ratio(alpha + eta) * gamma_ratio(2 * alpha + 2 * eta) * gamma_ra
        + (1.95319e-17 * t**(2 * alpha + beta + 3 * eta) * gamma_ratio(alpha + beta + eta) * gamma_ratio(2 * alpha + beta
          (gamma_ratio(alpha) * gamma_ratio(beta) * gamma_ratio(alpha + eta) * gamma_ratio(alpha + beta + 2 * eta) * gamma
        + (1.95319e-17 * t**(2 * alpha + beta + 3 * eta) * gamma_ratio(alpha + beta + eta) * gamma_ratio(2 * alpha + beta
          (gamma_ratio(alpha)**2 * gamma_ratio(beta + eta) * gamma_ratio(alpha + beta + 2 * eta) * gamma_ratio(2 * alpha +
        - (4.40795e-20 * t**(2 * beta + 3 * eta) * gamma_ratio(2 * beta + eta)) / \
          (gamma_ratio(beta) * gamma_ratio(beta + eta) * gamma_ratio(2 * beta + 3 * eta)) \
        - (4.40795e-20 * t**(2 * beta + 3 * eta) * gamma_ratio(2 * beta + 2 * eta)) / \
          (gamma_ratio(beta) * gamma_ratio(beta + 2 * eta) * gamma_ratio(2 * beta + 3 * eta)) \
        - (4.40795e-20 * t**(2 * beta + 3 * eta) * gamma_ratio(beta + eta) * gamma_ratio(2 * beta + 2 * eta)) / \
          (gamma_ratio(beta)**2 * gamma_ratio(eta) * gamma_ratio(beta + 2 * eta) * gamma_ratio(2 * beta + 3 * eta)) \
        + (3.68678e-23 * t**(alpha + 2 * beta + 3 * eta) * gamma_ratio(alpha + beta + eta) * gamma_ratio(alpha + 2 * beta
          (gamma_ratio(beta)**2 * gamma_ratio(alpha + eta) * gamma_ratio(alpha + beta + 2 * eta) * gamma_ratio(alpha + 2 *
        + (3.68678e-23 * t**(alpha + 2 * beta + 3 * eta) * gamma_ratio(alpha + beta + eta) * gamma_ratio(alpha + 2 * beta
          (gamma_ratio(alpha) * gamma_ratio(beta) * gamma_ratio(beta + eta) * gamma_ratio(alpha + beta + 2 * eta) * gamma_
        + (3.68678e-23 * t**(alpha + 2 * beta + 3 * eta) * gamma_ratio(2 * beta + eta) * gamma_ratio(alpha + 2 * beta + 2
          (gamma_ratio(alpha) * gamma_ratio(beta) * gamma_ratio(beta + eta) * gamma_ratio(2 * beta + 2 * eta) * gamma_rati
        + (6.95905e-29 * t**(3 * beta + 3 * eta) * gamma_ratio(2 * beta + eta) * gamma_ratio(3 * beta + 2 * eta)) / \
          (gamma_ratio(beta)**2 * gamma_ratio(beta + eta) * gamma_ratio(2 * beta + 2 * eta) * gamma_ratio(3 * beta + 3 * e

    return I0 + I1 + I2 + I3

```

```python
def recovered(t, alpha, beta, eta, rho):
    # R0
    R0 = 50

    # R1
    R1 = (14.8487 * t**rho) / gamma_ratio(rho)

    # R2 - ALL terms (with corrected values)
    R2 = -((0.72817 * t**(eta + rho)) / gamma_ratio(eta + rho)) \
         + (0.000609037 * t**(alpha + eta + rho)) / gamma_ratio(alpha + eta + rho) \
         + (1.1496e-9 * t**(beta + eta + rho)) / gamma_ratio(beta + eta + rho) \
         - (0.000376712 * t**(2 * rho)) / gamma_ratio(2 * rho)

    # R3 - ALL terms (with corrected values)
    R3 = -((0.0111114 * t**(alpha + eta + rho)) / gamma_ratio(alpha + eta + rho)) \
         - (0.0000102985 * t**(2 * alpha + eta + rho)) / gamma_ratio(2 * alpha + eta + rho) \
         - (6.57855e-8 * t**(beta + eta + rho)) / gamma_ratio(beta + eta + rho) \
         + (4.34101e-9 * t**(alpha + beta + eta + rho)) / gamma_ratio(alpha + beta + eta + rho) \
         - (3.23957e-12 * t**(2 * beta + eta + rho)) / gamma_ratio(2 * beta + eta + rho) \
         + (0.0357058 * t**(2 * eta + rho)) / gamma_ratio(2 * eta + rho) \
         - (0.0000298641 * t**(alpha + 2 * eta + rho)) / gamma_ratio(alpha + 2 * eta + rho) \
         - (0.0000298641 * t**(alpha + 2 * eta + rho) * gamma_ratio(alpha + eta)) / \
           (gamma_ratio(alpha) * gamma_ratio(eta) * gamma_ratio(alpha + 2 * eta + rho)) \
         + (2.49782e-8 * t**(2 * alpha + 2 * eta + rho) * gamma_ratio(2 * alpha + eta)) / \
           (gamma_ratio(alpha) * gamma_ratio(alpha + eta) * gamma_ratio(2 * alpha + 2 * eta + rho)) \
         - (5.63705e-11 * t**(beta + 2 * eta + rho)) / gamma_ratio(beta + 2 * eta + rho) \
         - (5.63705e-11 * t**(beta + 2 * eta + rho) * gamma_ratio(beta + eta)) / \
           (gamma_ratio(beta) * gamma_ratio(eta) * gamma_ratio(beta + 2 * eta + rho)) \
         + (4.7148e-14 * t**(alpha + beta + 2 * eta + rho) * gamma_ratio(alpha + beta + eta)) / \
           (gamma_ratio(beta) * gamma_ratio(alpha + eta) * gamma_ratio(alpha + beta + 2 * eta + rho)) \
         + (4.7148e-14 * t**(alpha + beta + 2 * eta + rho) * gamma_ratio(alpha + beta + eta)) / \
           (gamma_ratio(alpha) * gamma_ratio(beta + eta) * gamma_ratio(alpha + beta + 2 * eta + rho)) \
         + (8.8995e-20 * t**(2 * beta + 2 * eta + rho) * gamma_ratio(2 * beta + eta)) / \
           (gamma_ratio(beta) * gamma_ratio(beta + eta) * gamma_ratio(2 * beta + 2 * eta + rho)) \
         + (0.0000184737 * t**(eta + 2 * rho)) / gamma_ratio(eta + 2 * rho) \
         - (1.54513e-8 * t**(alpha + eta + 2 * rho)) / gamma_ratio(alpha + eta + 2 * rho) \
         - (2.91654e-14 * t**(beta + eta + 2 * rho)) / gamma_ratio(beta + eta + 2 * rho) \
         + (9.55718e-9 * t**(3 * rho)) / gamma_ratio(3 * rho)

    return R0 + R1 + R2 + R3



alpha_S = 1  # Fractional order for Susceptible
alpha_V = 1  # Fractional order for Vaccinated
alpha_I = 1  # Fractional order for Infected
alpha_R = 1  # Fractional order for Recovered


t_values = [0.01, 0.03, 0.05, 0.07, 0.1, 0.5, 1, 5, 10, 20]

print("=" * 100)
print("HOMOGENEOUS FRACTIONAL ORDERS SVIR MODEL")
print("α_S = 1, α_V = 1, α_I = 1, α_R = 1")
print("=" * 100)
print(f"{'t':<10} {'S(t)':<18} {'V(t)':<18} {'I(t)':<18} {'R(t)':<18}")
print("-" * 100)

for t in t_values:
    S_val = susceptible(t, alpha_S, alpha_V, alpha_I, alpha_R)
    V_val = vaccinated(t, alpha_S, alpha_V, alpha_I, alpha_R)
    I_val = infected(t, alpha_S, alpha_V, alpha_I, alpha_R)
    R_val = recovered(t, alpha_S, alpha_V, alpha_I, alpha_R)

    print(f"{t:<10} {S_val:<18.8f} {V_val:<18.8f} {I_val:<18.8f} {R_val:<18.8f}")

print("=" * 100)


print("\n" + "=" * 100)
print("TOTAL POPULATION N(t) = S(t) + V(t) + I(t) + R(t)")
print("=" * 100)
print(f"{'t':<10} {'Total N(t)':<20}")
print("-" * 100)

for t in t_values:
    S_val = susceptible(t, alpha_S, alpha_V, alpha_I, alpha_R)
```

```
    V_val = vaccinated(t, alpha_S, alpha_V, alpha_I, alpha_R)
    I_val = infected(t, alpha_S, alpha_V, alpha_I, alpha_R)
    R_val = recovered(t, alpha_S, alpha_V, alpha_I, alpha_R)
    total = S_val + V_val + I_val + R_val
    print(f"{t:<10} {total:<20.8f}")

print("=" * 100)
```

SVIR Model

```
import numpy as np
import matplotlib.pyplot as plt
from scipy.special import gamma

def gamma_ratio(x):
    return gamma(1 + x)

def susceptible(t, alpha, beta, eta, rho):
    S0 = 500 + (0.410126 * t**alpha) / gamma_ratio(alpha)


    S1 = -((7.4824 * t**alpha) / gamma_ratio(alpha)) \
         - (0.00693498 * t**(2 * alpha)) / gamma_ratio(2 * alpha) \
         + (2.15056e-6 * t**(alpha + beta)) / gamma_ratio(alpha + beta)


    S2 = (0.126523 * t**(2 * alpha)) / gamma_ratio(2 * alpha) \
         + (0.000117266 * t**(3 * alpha)) / gamma_ratio(3 * alpha) \
         - (0.000123065 * t**(alpha + beta)) / gamma_ratio(alpha + beta) \
         + (2.11013e-6 * t**(2 * alpha + beta)) / gamma_ratio(2 * alpha + beta) \
         - (6.06029e-9 * t**(alpha + 2 * beta)) / gamma_ratio(alpha + 2 * beta) \
         + (0.367763 * t**(alpha + eta)) / gamma_ratio(alpha + eta) \
         - (0.000307595 * t**(2 * alpha + eta)) / gamma_ratio(2 * alpha + eta) \
         + (0.000301658 * t**(2 * alpha + eta) * gamma_ratio(alpha + eta)) / \
           (gamma_ratio(alpha) * gamma_ratio(eta) * gamma_ratio(2 * alpha + eta)) \
         - (2.52305e-7 * t**(3 * alpha + eta) * gamma_ratio(2 * alpha + eta)) / \
           (gamma_ratio(alpha) * gamma_ratio(alpha + eta) * gamma_ratio(3 * alpha + eta)) \
         - (5.80605e-10 * t**(alpha + beta + eta)) / gamma_ratio(alpha + beta + eta) \
         - (4.76242e-13 * t**(2 * alpha + beta + eta) * gamma_ratio(alpha + beta + eta)) / \
           (gamma_ratio(alpha) * gamma_ratio(beta + eta) * gamma_ratio(2 * alpha + beta + eta))


    S3 = -((0.00213943 * t**(3 * alpha)) / gamma_ratio(3 * alpha)) \
         - (1.9829e-6 * t**(4 * alpha)) / gamma_ratio(4 * alpha) \
         - (0.00003708 * t**(2 * alpha + beta)) / gamma_ratio(2 * alpha + beta) \
         - (7.1977e-8 * t**(3 * alpha + beta)) / gamma_ratio(3 * alpha + beta) \
         + (3.46797e-7 * t**(alpha + 2 * beta)) / gamma_ratio(alpha + 2 * beta) \
         - (5.93509e-9 * t**(2 * alpha + 2 * beta)) / gamma_ratio(2 * alpha + 2 * beta) \
         + (1.70779e-11 * t**(alpha + 3 * beta)) / gamma_ratio(alpha + 3 * beta) \
         - (0.000606852 * t**(2 * alpha + eta)) / gamma_ratio(2 * alpha + eta) \
         - (0.00550349 * t**(2 * alpha + eta) * gamma_ratio(alpha + eta)) / \
           (gamma_ratio(alpha) * gamma_ratio(eta) * gamma_ratio(2 * alpha + eta)) \
         + (0.0000104025 * t**(3 * alpha + eta)) / gamma_ratio(3 * alpha + eta) \
         - (5.10086e-6 * t**(3 * alpha + eta) * gamma_ratio(alpha + eta)) / \
           (gamma_ratio(alpha) * gamma_ratio(eta) * gamma_ratio(3 * alpha + eta)) \
         - (5.10085e-6 * t**(3 * alpha + eta) * gamma_ratio(2 * alpha + eta)) / \
           (gamma_ratio(2 * alpha) * gamma_ratio(eta) * gamma_ratio(3 * alpha + eta)) \
         + (9.20618e-6 * t**(3 * alpha + eta) * gamma_ratio(2 * alpha + eta)) / \
           (gamma_ratio(alpha) * gamma_ratio(alpha + eta) * gamma_ratio(3 * alpha + eta)) \
         + (4.26633e-9 * t**(4 * alpha + eta) * gamma_ratio(2 * alpha + eta)) / \
           (gamma_ratio(alpha) * gamma_ratio(alpha + eta) * gamma_ratio(4 * alpha + eta)) \
         + (4.26632e-9 * t**(4 * alpha + eta) * gamma_ratio(3 * alpha + eta)) / \
           (gamma_ratio(2 * alpha) * gamma_ratio(alpha + eta) * gamma_ratio(4 * alpha + eta)) \
         + (4.26634e-9 * t**(4 * alpha + eta) * gamma_ratio(3 * alpha + eta)) / \
           (gamma_ratio(alpha) * gamma_ratio(2 * alpha + eta) * gamma_ratio(4 * alpha + eta)) \
         + (7.48377e-7 * t**(alpha + beta + eta)) / gamma_ratio(alpha + beta + eta) \
         - (2.78076e-9 * t**(2 * alpha + beta + eta)) / gamma_ratio(2 * alpha + beta + eta) \
         + (1.58179e-9 * t**(2 * alpha + beta + eta) * gamma_ratio(alpha + beta + eta)) / \
           (gamma_ratio(alpha + beta) * gamma_ratio(eta) * gamma_ratio(2 * alpha + beta + eta)) \
         + (3.59415e-11 * t**(2 * alpha + beta + eta) * gamma_ratio(alpha + beta + eta)) / \
           (gamma_ratio(alpha) * gamma_ratio(beta + eta) * gamma_ratio(2 * alpha + beta + eta)) \
         + (8.05297e-15 * t**(3 * alpha + beta + eta) * gamma_ratio(alpha + beta + eta)) / \
           (gamma_ratio(alpha) * gamma_ratio(beta + eta) * gamma_ratio(3 * alpha + beta + eta)) \
         - (1.323e-12 * t**(3 * alpha + beta + eta) * gamma_ratio(2 * alpha + beta + eta)) / \
           (gamma_ratio(alpha + beta) * gamma_ratio(alpha + eta) * gamma_ratio(3 * alpha + beta + eta)) \
```

```
         + (8.05297e-15 * t**(3 * alpha + beta + eta) * gamma_ratio(2 * alpha + beta + eta)) / \
           (gamma_ratio(2 * alpha) * gamma_ratio(beta + eta) * gamma_ratio(3 * alpha + beta + eta)) \
         - (1.79835e-12 * t**(3 * alpha + beta + eta) * gamma_ratio(2 * alpha + beta + eta)) / \
           (gamma_ratio(alpha) * gamma_ratio(alpha + beta + eta) * gamma_ratio(3 * alpha + beta + eta)) \
         + (1.63502e-12 * t**(alpha + 2 * beta + eta)) / gamma_ratio(alpha + 2 * beta + eta) \
         + (1.58179e-12 * t**(alpha + 2 * beta + eta) * gamma_ratio(beta + eta)) / \
           (gamma_ratio(beta) * gamma_ratio(eta) * gamma_ratio(alpha + 2 * beta + eta)) \
         - (1.323e-15 * t**(2 * alpha + 2 * beta + eta) * gamma_ratio(alpha + beta + eta)) / \
           (gamma_ratio(beta) * gamma_ratio(alpha + eta) * gamma_ratio(2 * alpha + 2 * beta + eta)) \
         - (2.49725e-18 * t**(2 * alpha + 2 * beta + eta) * gamma_ratio(alpha + 2 * beta + eta)) / \
           (gamma_ratio(alpha + beta) * gamma_ratio(beta + eta) * gamma_ratio(2 * alpha + 2 * beta + eta)) \
         + (1.34205e-15 * t**(2 * alpha + 2 * beta + eta) * gamma_ratio(alpha + 2 * beta + eta)) / \
           (gamma_ratio(alpha) * gamma_ratio(2 * beta + eta) * gamma_ratio(2 * alpha + 2 * beta + eta)) \
         - (2.49725e-21 * t**(alpha + 3 * beta + eta) * gamma_ratio(2 * beta + eta)) / \
           (gamma_ratio(beta) * gamma_ratio(beta + eta) * gamma_ratio(alpha + 3 * beta + eta)) \
         - (0.0180333 * t**(alpha + 2 * eta)) / gamma_ratio(alpha + 2 * eta) \
         + (0.0000150829 * t**(2 * alpha + 2 * eta)) / gamma_ratio(2 * alpha + 2 * eta) \
         + (0.0000150829 * t**(2 * alpha + 2 * eta) * gamma_ratio(alpha + eta)) / \
           (gamma_ratio(alpha) * gamma_ratio(eta) * gamma_ratio(2 * alpha + 2 * eta)) \
         - (0.0000147918 * t**(2 * alpha + 2 * eta) * gamma_ratio(alpha + 2 * eta)) / \
           (gamma_ratio(alpha) * gamma_ratio(2 * eta) * gamma_ratio(2 * alpha + 2 * eta)) \
         - (1.26153e-8 * t**(3 * alpha + 2 * eta) * gamma_ratio(2 * alpha + eta)) / \
           (gamma_ratio(alpha) * gamma_ratio(alpha + eta) * gamma_ratio(3 * alpha + 2 * eta)) \
         + (1.23718e-8 * t**(3 * alpha + 2 * eta) * gamma_ratio(2 * alpha + 2 * eta)) / \
           (gamma_ratio(alpha) * gamma_ratio(alpha + 2 * eta) * gamma_ratio(3 * alpha + 2 * eta)) \
         + (1.23718e-8 * t**(3 * alpha + 2 * eta) * gamma_ratio(alpha + eta) * gamma_ratio(2 * alpha + 2 * eta)) / \
           (gamma_ratio(alpha)**2 * gamma_ratio(eta) * gamma_ratio(alpha + 2 * eta) * gamma_ratio(3 * alpha + 2 * eta)) \
         - (1.03477e-11 * t**(4 * alpha + 2 * eta) * gamma_ratio(2 * alpha + eta) * gamma_ratio(3 * alpha + 2 * eta)) / \
           (gamma_ratio(alpha)**2 * gamma_ratio(alpha + eta) * gamma_ratio(2 * alpha + 2 * eta) * gamma_ratio(4 * alpha +
         + (2.847e-11 * t**(alpha + beta + 2 * eta)) / gamma_ratio(alpha + beta + 2 * eta) \
         + (2.847e-11 * t**(alpha + beta + 2 * eta) * gamma_ratio(beta + eta)) / \
           (gamma_ratio(beta) * gamma_ratio(eta) * gamma_ratio(alpha + beta + 2 * eta)) \
         - (2.38121e-14 * t**(2 * alpha + beta + 2 * eta) * gamma_ratio(alpha + beta + eta)) / \
           (gamma_ratio(beta) * gamma_ratio(alpha + eta) * gamma_ratio(2 * alpha + beta + 2 * eta)) \
         - (2.38121e-14 * t**(2 * alpha + beta + 2 * eta) * gamma_ratio(alpha + beta + eta)) / \
           (gamma_ratio(alpha) * gamma_ratio(beta + eta) * gamma_ratio(2 * alpha + beta + 2 * eta)) \
         + (2.33525e-14 * t**(2 * alpha + beta + 2 * eta) * gamma_ratio(alpha + beta + 2 * eta)) / \
           (gamma_ratio(alpha) * gamma_ratio(beta + 2 * eta) * gamma_ratio(2 * alpha + beta + 2 * eta)) \
         + (2.33525e-14 * t**(2 * alpha + beta + 2 * eta) * gamma_ratio(beta + eta) * gamma_ratio(alpha + beta + 2 * eta))
           (gamma_ratio(alpha) * gamma_ratio(beta) * gamma_ratio(eta) * gamma_ratio(beta + 2 * eta) * gamma_ratio(2 * alph
         - (1.95319e-17 * t**(3 * alpha + beta + 2 * eta) * gamma_ratio(alpha + beta + eta) * gamma_ratio(2 * alpha + beta
           (gamma_ratio(alpha) * gamma_ratio(beta) * gamma_ratio(alpha + eta) * gamma_ratio(alpha + beta + 2 * eta) * gamm
         - (1.95319e-17 * t**(3 * alpha + beta + 2 * eta) * gamma_ratio(alpha + beta + eta) * gamma_ratio(2 * alpha + beta
           (gamma_ratio(alpha)**2 * gamma_ratio(beta + eta) * gamma_ratio(alpha + beta + 2 * eta) * gamma_ratio(3 * alpha
         - (4.4947e-20 * t**(alpha + 2 * beta + 2 * eta) * gamma_ratio(2 * beta + eta)) / \
           (gamma_ratio(beta) * gamma_ratio(beta + eta) * gamma_ratio(alpha + 2 * beta + 2 * eta)) \
         - (3.68678e-23 * t**(2 * alpha + 2 * beta + 2 * eta) * gamma_ratio(2 * beta + eta) * gamma_ratio(alpha + 2 * beta
           (gamma_ratio(alpha) * gamma_ratio(beta) * gamma_ratio(beta + eta) * gamma_ratio(2 * beta + 2 * eta) * gamma_rat

    return S0 + S1 + S2 + S3


def vaccinated(t, alpha, beta, eta, rho):

    V0 = 350 + (0.00077414 * t**beta) / gamma_ratio(beta)


    V1 = -((0.0443 * t**beta) / gamma_ratio(beta)) \
         + (0.000772677 * t**(alpha + beta)) / gamma_ratio(alpha + beta) \
         - (2.18153e-6 * t**(2 * beta)) / gamma_ratio(2 * beta)


    V2 = -((0.0140968 * t**(alpha + beta)) / gamma_ratio(alpha + beta)) \
         - (0.0000130655 * t**(2 * alpha + beta)) / gamma_ratio(2 * alpha + beta) \
         + (0.000124837 * t**(2 * beta)) / gamma_ratio(2 * beta) \
         - (2.17335e-6 * t**(alpha + 2 * beta)) / gamma_ratio(alpha + 2 * beta) \
         + (6.14755e-9 * t**(3 * beta)) / gamma_ratio(3 * beta) \
         + (0.000257434 * t**(beta + eta)) / gamma_ratio(beta + eta) \
         - (2.15316e-7 * t**(alpha + beta + eta)) / gamma_ratio(alpha + beta + eta) \
         - (4.06423e-13 * t**(2 * beta + eta)) / gamma_ratio(2 * beta + eta) \
         + (5.69399e-10 * t**(2 * beta + eta) * gamma_ratio(beta + eta)) / \
           (gamma_ratio(beta) * gamma_ratio(eta) * gamma_ratio(2 * beta + eta)) \
         - (4.76242e-13 * t**(alpha + 2 * beta + eta) * gamma_ratio(alpha + beta + eta)) / \
           (gamma_ratio(beta) * gamma_ratio(alpha + eta) * gamma_ratio(alpha + 2 * beta + eta)) \
         - (8.98939e-19 * t**(3 * beta + eta) * gamma_ratio(2 * beta + eta)) / \
           (gamma_ratio(beta) * gamma_ratio(beta + eta) * gamma_ratio(3 * beta + eta))

```

```

V3 = (0.000238369 * t**(2 * alpha + beta)) / gamma_ratio(2 * alpha + beta) \
     + (2.20929e-7 * t**(3 * alpha + beta)) / gamma_ratio(3 * alpha + beta) \
     + (0.0000394929 * t**(alpha + 2 * beta)) / gamma_ratio(alpha + 2 * beta) \
     + (4.07941e-8 * t**(2 * alpha + 2 * beta)) / gamma_ratio(2 * alpha + 2 * beta) \
     - (3.51791e-7 * t**(3 * beta)) / gamma_ratio(3 * beta) \
     + (6.11308e-9 * t**(alpha + 3 * beta)) / gamma_ratio(alpha + 3 * beta) \
     - (1.73238e-11 * t**(4 * beta)) / gamma_ratio(4 * beta) \
     + (0.000696794 * t**(alpha + beta + eta)) / gamma_ratio(alpha + beta + eta) \
     - (5.75868e-7 * t**(2 * alpha + beta + eta)) / gamma_ratio(2 * alpha + beta + eta) \
     + (5.68324e-7 * t**(2 * alpha + beta + eta) * gamma_ratio(alpha + eta)) / \
       (gamma_ratio(alpha) * gamma_ratio(eta) * gamma_ratio(2 * alpha + beta + eta)) \
     - (4.75343e-10 * t**(3 * alpha + beta + eta) * gamma_ratio(2 * alpha + eta)) / \
       (gamma_ratio(alpha) * gamma_ratio(alpha + eta) * gamma_ratio(3 * alpha + beta + eta)) \
     - (7.25426e-7 * t**(2 * beta + eta)) / gamma_ratio(2 * beta + eta) \
     - (3.25838e-8 * t**(2 * beta + eta) * gamma_ratio(beta + eta)) / \
       (gamma_ratio(beta) * gamma_ratio(eta) * gamma_ratio(2 * beta + eta)) \
     + (6.04132e-10 * t**(alpha + 2 * beta + eta)) / gamma_ratio(alpha + 2 * beta + eta) \
     + (5.68323e-10 * t**(alpha + 2 * beta + eta) * gamma_ratio(alpha + beta + eta)) / \
       (gamma_ratio(alpha + beta) * gamma_ratio(eta) * gamma_ratio(alpha + 2 * beta + eta)) \
     + (3.59415e-11 * t**(alpha + 2 * beta + eta) * gamma_ratio(alpha + beta + eta)) / \
       (gamma_ratio(beta) * gamma_ratio(alpha + eta) * gamma_ratio(alpha + 2 * beta + eta)) \
     - (8.9724e-16 * t**(2 * alpha + 2 * beta + eta) * gamma_ratio(alpha + beta + eta)) / \
       (gamma_ratio(alpha) * gamma_ratio(beta + eta) * gamma_ratio(2 * alpha + 2 * beta + eta)) \
     - (4.75342e-13 * t**(2 * alpha + 2 * beta + eta) * gamma_ratio(2 * alpha + beta + eta)) / \
       (gamma_ratio(alpha + beta) * gamma_ratio(alpha + eta) * gamma_ratio(2 * alpha + 2 * beta + eta)) \
     + (8.05299e-15 * t**(2 * alpha + 2 * beta + eta) * gamma_ratio(2 * alpha + beta + eta)) / \
       (gamma_ratio(beta) * gamma_ratio(2 * alpha + eta) * gamma_ratio(2 * alpha + 2 * beta + eta)) \
     + (2.2906e-15 * t**(3 * beta + eta)) / gamma_ratio(3 * beta + eta) \
     - (1.60457e-12 * t**(3 * beta + eta) * gamma_ratio(beta + eta)) / \
       (gamma_ratio(beta) * gamma_ratio(eta) * gamma_ratio(3 * beta + eta)) \
     - (1.60457e-12 * t**(3 * beta + eta) * gamma_ratio(2 * beta + eta)) / \
       (gamma_ratio(2 * beta) * gamma_ratio(eta) * gamma_ratio(3 * beta + eta)) \
     + (1.02883e-16 * t**(3 * beta + eta) * gamma_ratio(2 * beta + eta)) / \
       (gamma_ratio(beta) * gamma_ratio(beta + eta) * gamma_ratio(3 * beta + eta)) \
     + (1.34205e-15 * t**(alpha + 3 * beta + eta) * gamma_ratio(alpha + beta + eta)) / \
       (gamma_ratio(beta) * gamma_ratio(alpha + eta) * gamma_ratio(alpha + 3 * beta + eta)) \
     + (1.34205e-15 * t**(alpha + 3 * beta + eta) * gamma_ratio(alpha + 2 * beta + eta)) / \
       (gamma_ratio(2 * beta) * gamma_ratio(alpha + eta) * gamma_ratio(alpha + 3 * beta + eta)) \
     - (8.9724e-19 * t**(alpha + 3 * beta + eta) * gamma_ratio(alpha + 2 * beta + eta)) / \
       (gamma_ratio(alpha + beta) * gamma_ratio(beta + eta) * gamma_ratio(alpha + 3 * beta + eta)) \
     - (3.3945e-18 * t**(alpha + 3 * beta + eta) * gamma_ratio(alpha + 2 * beta + eta)) / \
       (gamma_ratio(beta) * gamma_ratio(alpha + beta + eta) * gamma_ratio(alpha + 3 * beta + eta)) \
     + (2.53321e-21 * t**(4 * beta + eta) * gamma_ratio(2 * beta + eta)) / \
       (gamma_ratio(beta) * gamma_ratio(beta + eta) * gamma_ratio(4 * beta + eta)) \
     + (2.53321e-21 * t**(4 * beta + eta) * gamma_ratio(3 * beta + eta)) / \
       (gamma_ratio(2 * beta) * gamma_ratio(beta + eta) * gamma_ratio(4 * beta + eta)) \
     + (2.53321e-21 * t**(4 * beta + eta) * gamma_ratio(3 * beta + eta)) / \
       (gamma_ratio(beta) * gamma_ratio(2 * beta + eta) * gamma_ratio(4 * beta + eta)) \
     - (0.0000126233 * t**(beta + 2 * eta)) / gamma_ratio(beta + 2 * eta) \
     + (1.0558e-8 * t**(alpha + beta + 2 * eta)) / gamma_ratio(alpha + beta + 2 * eta) \
     + (1.0558e-8 * t**(alpha + beta + 2 * eta) * gamma_ratio(alpha + eta)) / \
       (gamma_ratio(alpha) * gamma_ratio(eta) * gamma_ratio(alpha + beta + 2 * eta)) \
     - (8.83067e-12 * t**(2 * alpha + beta + 2 * eta) * gamma_ratio(2 * alpha + eta)) / \
       (gamma_ratio(alpha) * gamma_ratio(alpha + eta) * gamma_ratio(2 * alpha + beta + 2 * eta)) \
     + (1.9929e-14 * t**(2 * beta + 2 * eta)) / gamma_ratio(2 * beta + 2 * eta) \
     + (1.9929e-14 * t**(2 * beta + 2 * eta) * gamma_ratio(beta + eta)) / \
       (gamma_ratio(beta) * gamma_ratio(eta) * gamma_ratio(2 * beta + 2 * eta)) \
     - (2.79205e-11 * t**(2 * beta + 2 * eta) * gamma_ratio(beta + 2 * eta)) / \
       (gamma_ratio(beta) * gamma_ratio(2 * eta) * gamma_ratio(2 * beta + 2 * eta)) \
     - (1.66685e-17 * t**(alpha + 2 * beta + 2 * eta) * gamma_ratio(alpha + beta + eta)) / \
       (gamma_ratio(beta) * gamma_ratio(alpha + eta) * gamma_ratio(alpha + 2 * beta + 2 * eta)) \
     - (1.66685e-17 * t**(alpha + 2 * beta + 2 * eta) * gamma_ratio(alpha + beta + eta)) / \
       (gamma_ratio(alpha) * gamma_ratio(beta + eta) * gamma_ratio(alpha + 2 * beta + 2 * eta)) \
     + (2.33526e-14 * t**(alpha + 2 * beta + 2 * eta) * gamma_ratio(alpha + beta + 2 * eta)) / \
       (gamma_ratio(beta) * gamma_ratio(alpha + 2 * eta) * gamma_ratio(alpha + 2 * beta + 2 * eta)) \
     + (2.33526e-14 * t**(alpha + 2 * beta + 2 * eta) * gamma_ratio(alpha + eta) * gamma_ratio(alpha + beta + 2 * eta)
       (gamma_ratio(alpha) * gamma_ratio(beta) * gamma_ratio(eta) * gamma_ratio(alpha + 2 * eta) * gamma_ratio(alpha +
     - (1.95319e-17 * t**(2 * alpha + 2 * beta + 2 * eta) * gamma_ratio(2 * alpha + eta) * gamma_ratio(2 * alpha + bet
       (gamma_ratio(alpha) * gamma_ratio(beta) * gamma_ratio(alpha + eta) * gamma_ratio(2 * alpha + 2 * eta) * gamma_r
     - (3.14629e-23 * t**(3 * beta + 2 * eta) * gamma_ratio(2 * beta + eta)) / \
       (gamma_ratio(beta) * gamma_ratio(beta + eta) * gamma_ratio(3 * beta + 2 * eta)) \
     + (4.40795e-20 * t**(3 * beta + 2 * eta) * gamma_ratio(2 * beta + 2 * eta)) / \
       (gamma_ratio(beta) * gamma_ratio(beta + 2 * eta) * gamma_ratio(3 * beta + 2 * eta)) \
     + (4.40795e-20 * t**(3 * beta + 2 * eta) * gamma_ratio(beta + eta) * gamma_ratio(2 * beta + 2 * eta)) / \
       (gamma_ratio(beta)**2 * gamma_ratio(eta) * gamma_ratio(beta + 2 * eta) * gamma_ratio(3 * beta + 2 * eta)) \
     - (3.68678e-23 * t**(alpha + 3 * beta + 2 * eta) * gamma_ratio(alpha + beta + eta) * gamma_ratio(alpha + 2 * beta
```

```
           (gamma_ratio(beta)**2 * gamma_ratio(alpha + eta) * gamma_ratio(alpha + beta + 2 * eta) * gamma_ratio(alpha + 3
         - (3.68678e-23 * t**(alpha + 3 * beta + 2 * eta) * gamma_ratio(alpha + beta + eta) * gamma_ratio(alpha + 2 * beta
           (gamma_ratio(alpha) * gamma_ratio(beta) * gamma_ratio(beta + eta) * gamma_ratio(alpha + beta + 2 * eta) * gamma
         - (6.95905e-29 * t**(4 * beta + 2 * eta) * gamma_ratio(2 * beta + eta) * gamma_ratio(3 * beta + 2 * eta)) / \
           (gamma_ratio(beta)**2 * gamma_ratio(beta + eta) * gamma_ratio(2 * beta + 2 * eta) * gamma_ratio(4 * beta + 2 *

    return V0 + V1 + V2 + V3


def infected(t, alpha, beta, eta, rho):

    I0 = 150

    I1 = -((7.35525 * t**eta) / gamma_ratio(eta)) \
         + (0.00615189 * t**(alpha + eta)) / gamma_ratio(alpha + eta) \
         + (1.16121e-8 * t**(beta + eta)) / gamma_ratio(beta + eta)


    I2 = -((0.112236 * t**(alpha + eta)) / gamma_ratio(alpha + eta)) \
         - (0.000104025 * t**(2 * alpha + eta)) / gamma_ratio(2 * alpha + eta) \
         - (6.645e-7 * t**(beta + eta)) / gamma_ratio(beta + eta) \
         + (4.38486e-8 * t**(alpha + beta + eta)) / gamma_ratio(alpha + beta + eta) \
         - (3.27229e-11 * t**(2 * beta + eta)) / gamma_ratio(2 * beta + eta) \
         + (0.360665 * t**(2 * eta)) / gamma_ratio(2 * eta) \
         - (0.000301658 * t**(alpha + 2 * eta)) / gamma_ratio(alpha + 2 * eta) \
         - (0.000301658 * t**(alpha + 2 * eta) * gamma_ratio(alpha + eta)) / \
           (gamma_ratio(alpha) * gamma_ratio(eta) * gamma_ratio(alpha + 2 * eta)) \
         + (2.52305e-7 * t**(2 * alpha + 2 * eta) * gamma_ratio(2 * alpha + eta)) / \
           (gamma_ratio(alpha) * gamma_ratio(alpha + eta) * gamma_ratio(2 * alpha + 2 * eta)) \
         - (5.69399e-10 * t**(beta + 2 * eta)) / gamma_ratio(beta + 2 * eta) \
         - (5.69399e-10 * t**(beta + 2 * eta) * gamma_ratio(beta + eta)) / \
           (gamma_ratio(beta) * gamma_ratio(eta) * gamma_ratio(beta + 2 * eta)) \
         + (4.76242e-13 * t**(alpha + beta + 2 * eta) * gamma_ratio(alpha + beta + eta)) / \
           (gamma_ratio(beta) * gamma_ratio(alpha + eta) * gamma_ratio(alpha + beta + 2 * eta)) \
         + (4.76242e-13 * t**(alpha + beta + 2 * eta) * gamma_ratio(alpha + beta + eta)) / \
           (gamma_ratio(alpha) * gamma_ratio(beta + eta) * gamma_ratio(alpha + beta + 2 * eta)) \
         + (8.98939e-19 * t**(2 * beta + 2 * eta) * gamma_ratio(2 * beta + eta)) / \
           (gamma_ratio(beta) * gamma_ratio(beta + eta) * gamma_ratio(2 * beta + 2 * eta))


    I3 = (0.00189785 * t**(2 * alpha + eta)) / gamma_ratio(2 * alpha + eta) \
         + (1.75899e-6 * t**(3 * alpha + eta)) / gamma_ratio(3 * alpha + eta) \
         - (2.05743e-6 * t**(alpha + beta + eta)) / gamma_ratio(alpha + beta + eta) \
         + (3.1456e-8 * t**(2 * alpha + beta + eta)) / gamma_ratio(2 * alpha + beta + eta) \
         + (1.87256e-9 * t**(2 * beta + eta)) / gamma_ratio(2 * beta + eta) \
         - (1.23505e-10 * t**(alpha + 2 * beta + eta)) / gamma_ratio(alpha + 2 * beta + eta) \
         + (9.22133e-14 * t**(3 * beta + eta)) / gamma_ratio(3 * beta + eta) \
         + (0.0110199 * t**(alpha + 2 * eta)) / gamma_ratio(alpha + 2 * eta) \
         + (0.00550349 * t**(alpha + 2 * eta) * gamma_ratio(alpha + eta)) / \
           (gamma_ratio(alpha) * gamma_ratio(eta) * gamma_ratio(alpha + 2 * eta)) \
         + (4.86941e-7 * t**(2 * alpha + 2 * eta)) / gamma_ratio(2 * alpha + 2 * eta) \
         + (4.52487e-6 * t**(2 * alpha + 2 * eta) * gamma_ratio(alpha + eta)) / \
           (gamma_ratio(alpha) * gamma_ratio(eta) * gamma_ratio(2 * alpha + 2 * eta)) \
         + (5.10085e-6 * t**(2 * alpha + 2 * eta) * gamma_ratio(2 * alpha + eta)) / \
           (gamma_ratio(2 * alpha) * gamma_ratio(eta) * gamma_ratio(2 * alpha + 2 * eta)) \
         - (9.20618e-6 * t**(2 * alpha + 2 * eta) * gamma_ratio(2 * alpha + eta)) / \
           (gamma_ratio(alpha) * gamma_ratio(alpha + eta) * gamma_ratio(2 * alpha + 2 * eta)) \
         - (3.78458e-9 * t**(3 * alpha + 2 * eta) * gamma_ratio(2 * alpha + eta)) / \
           (gamma_ratio(alpha) * gamma_ratio(alpha + eta) * gamma_ratio(3 * alpha + 2 * eta)) \
         - (4.26632e-9 * t**(3 * alpha + 2 * eta) * gamma_ratio(3 * alpha + eta)) / \
           (gamma_ratio(2 * alpha) * gamma_ratio(alpha + eta) * gamma_ratio(3 * alpha + 2 * eta)) \
         - (4.26634e-9 * t**(3 * alpha + 2 * eta) * gamma_ratio(3 * alpha + eta)) / \
           (gamma_ratio(alpha) * gamma_ratio(2 * alpha + eta) * gamma_ratio(3 * alpha + 2 * eta)) \
         + (3.64453e-8 * t**(beta + 2 * eta)) / gamma_ratio(beta + 2 * eta) \
         + (3.25838e-8 * t**(beta + 2 * eta) * gamma_ratio(beta + eta)) / \
           (gamma_ratio(beta) * gamma_ratio(eta) * gamma_ratio(beta + 2 * eta)) \
         - (2.16205e-9 * t**(alpha + beta + 2 * eta)) / gamma_ratio(alpha + beta + 2 * eta) \
         - (2.15011e-9 * t**(alpha + beta + 2 * eta) * gamma_ratio(alpha + beta + eta)) / \
           (gamma_ratio(alpha + beta) * gamma_ratio(eta) * gamma_ratio(alpha + beta + 2 * eta)) \
         - (3.59415e-11 * t**(alpha + beta + 2 * eta) * gamma_ratio(alpha + beta + eta)) / \
           (gamma_ratio(beta) * gamma_ratio(alpha + eta) * gamma_ratio(alpha + beta + 2 * eta)) \
         - (3.59415e-11 * t**(alpha + beta + 2 * eta) * gamma_ratio(alpha + beta + eta)) / \
           (gamma_ratio(alpha) * gamma_ratio(beta + eta) * gamma_ratio(alpha + beta + 2 * eta)) \
         - (7.14363e-15 * t**(2 * alpha + beta + 2 * eta) * gamma_ratio(alpha + beta + eta)) / \
           (gamma_ratio(alpha) * gamma_ratio(beta + eta) * gamma_ratio(2 * alpha + beta + 2 * eta)) \
         + (1.79834e-12 * t**(2 * alpha + beta + 2 * eta) * gamma_ratio(2 * alpha + beta + eta)) / \
           (gamma_ratio(alpha + beta) * gamma_ratio(alpha + eta) * gamma_ratio(2 * alpha + beta + 2 * eta)) \
```

```
        - (8.05299e-15 * t**(2 * alpha + beta + 2 * eta) * gamma_ratio(2 * alpha + beta + eta)) / \
          (gamma_ratio(beta) * gamma_ratio(2 * alpha + eta) * gamma_ratio(2 * alpha + beta + 2 * eta)) \
        - (8.05297e-15 * t**(2 * alpha + beta + 2 * eta) * gamma_ratio(2 * alpha + beta + eta)) / \
          (gamma_ratio(2 * alpha) * gamma_ratio(beta + eta) * gamma_ratio(2 * alpha + beta + 2 * eta)) \
        + (1.79835e-12 * t**(2 * alpha + beta + 2 * eta) * gamma_ratio(2 * alpha + beta + eta)) / \
          (gamma_ratio(alpha) * gamma_ratio(alpha + beta + eta) * gamma_ratio(2 * alpha + beta + 2 * eta)) \
        + (1.60456e-12 * t**(2 * beta + 2 * eta)) / gamma_ratio(2 * beta + 2 * eta) \
        + (8.54099e-15 * t**(2 * beta + 2 * eta) * gamma_ratio(beta + eta)) / \
          (gamma_ratio(beta) * gamma_ratio(eta) * gamma_ratio(2 * beta + 2 * eta)) \
        + (1.60457e-12 * t**(2 * beta + 2 * eta) * gamma_ratio(2 * beta + eta)) / \
          (gamma_ratio(2 * beta) * gamma_ratio(eta) * gamma_ratio(2 * beta + 2 * eta)) \
        - (1.02883e-16 * t**(2 * beta + 2 * eta) * gamma_ratio(2 * beta + eta)) / \
          (gamma_ratio(beta) * gamma_ratio(beta + eta) * gamma_ratio(2 * beta + 2 * eta)) \
        - (7.14363e-18 * t**(alpha + 2 * beta + 2 * eta) * gamma_ratio(alpha + beta + eta)) / \
          (gamma_ratio(beta) * gamma_ratio(alpha + eta) * gamma_ratio(alpha + 2 * beta + 2 * eta)) \
        - (1.34205e-15 * t**(alpha + 2 * beta + 2 * eta) * gamma_ratio(alpha + 2 * beta + eta)) / \
          (gamma_ratio(2 * beta) * gamma_ratio(alpha + eta) * gamma_ratio(alpha + 2 * beta + 2 * eta)) \
        + (3.39449e-18 * t**(alpha + 2 * beta + 2 * eta) * gamma_ratio(alpha + 2 * beta + eta)) / \
          (gamma_ratio(alpha + beta) * gamma_ratio(beta + eta) * gamma_ratio(alpha + 2 * beta + 2 * eta)) \
        + (3.3945e-18 * t**(alpha + 2 * beta + 2 * eta) * gamma_ratio(alpha + 2 * beta + eta)) / \
          (gamma_ratio(beta) * gamma_ratio(alpha + beta + eta) * gamma_ratio(alpha + 2 * beta + 2 * eta)) \
        - (1.34205e-15 * t**(alpha + 2 * beta + 2 * eta) * gamma_ratio(alpha + 2 * beta + eta)) / \
          (gamma_ratio(alpha) * gamma_ratio(2 * beta + eta) * gamma_ratio(alpha + 2 * beta + 2 * eta)) \
        - (1.34841e-23 * t**(3 * beta + 2 * eta) * gamma_ratio(2 * beta + eta)) / \
          (gamma_ratio(beta) * gamma_ratio(beta + eta) * gamma_ratio(3 * beta + 2 * eta)) \
        - (2.53321e-21 * t**(3 * beta + 2 * eta) * gamma_ratio(3 * beta + eta)) / \
          (gamma_ratio(2 * beta) * gamma_ratio(beta + eta) * gamma_ratio(3 * beta + 2 * eta)) \
        - (2.53321e-21 * t**(3 * beta + 2 * eta) * gamma_ratio(3 * beta + eta)) / \
          (gamma_ratio(beta) * gamma_ratio(2 * beta + eta) * gamma_ratio(3 * beta + 2 * eta)) \
        - (0.0176852 * t**(3 * eta)) / gamma_ratio(3 * eta) \
        + (0.0000147918 * t**(alpha + 3 * eta)) / gamma_ratio(alpha + 3 * eta) \
        + (0.0000147918 * t**(alpha + 3 * eta) * gamma_ratio(alpha + eta)) / \
          (gamma_ratio(alpha) * gamma_ratio(eta) * gamma_ratio(alpha + 3 * eta)) \
        + (0.0000147918 * t**(alpha + 3 * eta) * gamma_ratio(alpha + 2 * eta)) / \
          (gamma_ratio(alpha) * gamma_ratio(2 * eta) * gamma_ratio(alpha + 3 * eta)) \
        - (1.23718e-8 * t**(2 * alpha + 3 * eta) * gamma_ratio(2 * alpha + eta)) / \
          (gamma_ratio(alpha) * gamma_ratio(alpha + eta) * gamma_ratio(2 * alpha + 3 * eta)) \
        - (1.23718e-8 * t**(2 * alpha + 3 * eta) * gamma_ratio(2 * alpha + 2 * eta)) / \
          (gamma_ratio(alpha) * gamma_ratio(alpha + 2 * eta) * gamma_ratio(2 * alpha + 3 * eta)) \
        - (1.23718e-8 * t**(2 * alpha + 3 * eta) * gamma_ratio(alpha + eta) * gamma_ratio(2 * alpha + 2 * eta)) / \
          (gamma_ratio(alpha)**2 * gamma_ratio(eta) * gamma_ratio(alpha + 2 * eta) * gamma_ratio(2 * alpha + 3 * eta)) \
        + (1.03477e-11 * t**(3 * alpha + 3 * eta) * gamma_ratio(2 * alpha + eta) * gamma_ratio(3 * alpha + 2 * eta)) / \
          (gamma_ratio(alpha)**2 * gamma_ratio(alpha + eta) * gamma_ratio(2 * alpha + 2 * eta) * gamma_ratio(3 * alpha +
        + (2.79205e-11 * t**(beta + 3 * eta)) / gamma_ratio(beta + 3 * eta) \
        + (2.79205e-11 * t**(beta + 3 * eta) * gamma_ratio(beta + eta)) / \
          (gamma_ratio(beta) * gamma_ratio(eta) * gamma_ratio(beta + 3 * eta)) \
        + (2.79205e-11 * t**(beta + 3 * eta) * gamma_ratio(beta + 2 * eta)) / \
          (gamma_ratio(beta) * gamma_ratio(2 * eta) * gamma_ratio(beta + 3 * eta)) \
        - (2.33525e-14 * t**(alpha + beta + 3 * eta) * gamma_ratio(alpha + beta + eta)) / \
          (gamma_ratio(beta) * gamma_ratio(alpha + eta) * gamma_ratio(alpha + beta + 3 * eta)) \
        - (2.33525e-14 * t**(alpha + beta + 3 * eta) * gamma_ratio(alpha + beta + eta)) / \
          (gamma_ratio(alpha) * gamma_ratio(beta + eta) * gamma_ratio(alpha + beta + 3 * eta)) \
        - (2.33526e-14 * t**(alpha + beta + 3 * eta) * gamma_ratio(alpha + beta + 2 * eta)) / \
          (gamma_ratio(beta) * gamma_ratio(alpha + 2 * eta) * gamma_ratio(alpha + beta + 3 * eta)) \
        - (2.33526e-14 * t**(alpha + beta + 3 * eta) * gamma_ratio(alpha + eta) * gamma_ratio(alpha + beta + 2 * eta)) /
          (gamma_ratio(alpha) * gamma_ratio(beta) * gamma_ratio(eta) * gamma_ratio(alpha + 2 * eta) * gamma_ratio(alpha +
        - (2.33525e-14 * t**(alpha + beta + 3 * eta) * gamma_ratio(alpha + beta + 2 * eta)) / \
          (gamma_ratio(alpha) * gamma_ratio(beta + 2 * eta) * gamma_ratio(alpha + beta + 3 * eta)) \
        - (2.33525e-14 * t**(alpha + beta + 3 * eta) * gamma_ratio(beta + eta) * gamma_ratio(alpha + beta + 2 * eta)) / \
          (gamma_ratio(alpha) * gamma_ratio(beta) * gamma_ratio(eta) * gamma_ratio(beta + 2 * eta) * gamma_ratio(alpha +
        + (1.95319e-17 * t**(2 * alpha + beta + 3 * eta) * gamma_ratio(2 * alpha + eta) * gamma_ratio(2 * alpha + beta +
          (gamma_ratio(alpha) * gamma_ratio(beta) * gamma_ratio(alpha + eta) * gamma_ratio(2 * alpha + 2 * eta) * gamma_r
        + (1.95319e-17 * t**(2 * alpha + beta + 3 * eta) * gamma_ratio(alpha + beta + eta) * gamma_ratio(2 * alpha + beta
          (gamma_ratio(alpha) * gamma_ratio(beta) * gamma_ratio(alpha + eta) * gamma_ratio(alpha + beta + 2 * eta) * gamm
        + (1.95319e-17 * t**(2 * alpha + beta + 3 * eta) * gamma_ratio(alpha + beta + eta) * gamma_ratio(2 * alpha + beta
          (gamma_ratio(alpha)**2 * gamma_ratio(beta + eta) * gamma_ratio(alpha + beta + 2 * eta) * gamma_ratio(2 * alpha
        - (4.40795e-20 * t**(2 * beta + 3 * eta) * gamma_ratio(2 * beta + eta)) / \
          (gamma_ratio(beta) * gamma_ratio(beta + eta) * gamma_ratio(2 * beta + 3 * eta)) \
        - (4.40795e-20 * t**(2 * beta + 3 * eta) * gamma_ratio(2 * beta + 2 * eta)) / \
          (gamma_ratio(beta) * gamma_ratio(beta + 2 * eta) * gamma_ratio(2 * beta + 3 * eta)) \
        - (4.40795e-20 * t**(2 * beta + 3 * eta) * gamma_ratio(beta + eta) * gamma_ratio(2 * beta + 2 * eta)) / \
          (gamma_ratio(beta)**2 * gamma_ratio(eta) * gamma_ratio(beta + 2 * eta) * gamma_ratio(2 * beta + 3 * eta)) \
        + (3.68678e-23 * t**(alpha + 2 * beta + 3 * eta) * gamma_ratio(alpha + beta + eta) * gamma_ratio(alpha + 2 * beta
          (gamma_ratio(beta)**2 * gamma_ratio(alpha + eta) * gamma_ratio(alpha + beta + 2 * eta) * gamma_ratio(alpha + 2
        + (3.68678e-23 * t**(alpha + 2 * beta + 3 * eta) * gamma_ratio(alpha + beta + eta) * gamma_ratio(alpha + 2 * beta
          (gamma_ratio(alpha) * gamma_ratio(beta) * gamma_ratio(beta + eta) * gamma_ratio(alpha + beta + 2 * eta) * gamma
        + (3.68678e-23 * t**(alpha + 2 * beta + 3 * eta) * gamma_ratio(2 * beta + eta) * gamma_ratio(alpha + 2 * beta + 2
```

```
        (gamma_ratio(alpha) * gamma_ratio(beta) * gamma_ratio(beta + eta) * gamma_ratio(2 * beta + 2 * eta) * gamma_rat
      + (6.95905e-29 * t**(3 * beta + 3 * eta) * gamma_ratio(2 * beta + eta) * gamma_ratio(3 * beta + 2 * eta)) / \
        (gamma_ratio(beta)**2 * gamma_ratio(beta + eta) * gamma_ratio(2 * beta + 2 * eta) * gamma_ratio(3 * beta + 3 *

    return I0 + I1 + I2 + I3


def recovered(t, alpha, beta, eta, rho):

    R0 = 50


    R1 = (14.8487 * t**rho) / gamma_ratio(rho)


    R2 = -((0.72817 * t**(eta + rho)) / gamma_ratio(eta + rho)) \
         + (0.000609037 * t**(alpha + eta + rho)) / gamma_ratio(alpha + eta + rho) \
         + (1.1496e-9 * t**(beta + eta + rho)) / gamma_ratio(beta + eta + rho) \
         - (0.000376712 * t**(2 * rho)) / gamma_ratio(2 * rho)


    R3 = -((0.0111114 * t**(alpha + eta + rho)) / gamma_ratio(alpha + eta + rho)) \
         - (0.0000102985 * t**(2 * alpha + eta + rho)) / gamma_ratio(2 * alpha + eta + rho) \
         - (6.57855e-8 * t**(beta + eta + rho)) / gamma_ratio(beta + eta + rho) \
         + (4.34101e-9 * t**(alpha + beta + eta + rho)) / gamma_ratio(alpha + beta + eta + rho) \
         - (3.23957e-12 * t**(2 * beta + eta + rho)) / gamma_ratio(2 * beta + eta + rho) \
         + (0.0357058 * t**(2 * eta + rho)) / gamma_ratio(2 * eta + rho) \
         - (0.0000298641 * t**(alpha + 2 * eta + rho)) / gamma_ratio(alpha + 2 * eta + rho) \
         - (0.0000298641 * t**(alpha + 2 * eta + rho) * gamma_ratio(alpha + eta)) / \
           (gamma_ratio(alpha) * gamma_ratio(eta) * gamma_ratio(alpha + 2 * eta + rho)) \
         + (2.49782e-8 * t**(2 * alpha + 2 * eta + rho) * gamma_ratio(2 * alpha + eta)) / \
           (gamma_ratio(alpha) * gamma_ratio(alpha + eta) * gamma_ratio(2 * alpha + 2 * eta + rho)) \
         - (5.63705e-11 * t**(beta + 2 * eta + rho)) / gamma_ratio(beta + 2 * eta + rho) \
         - (5.63705e-11 * t**(beta + 2 * eta + rho) * gamma_ratio(beta + eta)) / \
           (gamma_ratio(beta) * gamma_ratio(eta) * gamma_ratio(beta + 2 * eta + rho)) \
         + (4.7148e-14 * t**(alpha + beta + 2 * eta + rho) * gamma_ratio(alpha + beta + eta)) / \
           (gamma_ratio(beta) * gamma_ratio(alpha + eta) * gamma_ratio(alpha + beta + 2 * eta + rho)) \
         + (4.7148e-14 * t**(alpha + beta + 2 * eta + rho) * gamma_ratio(alpha + beta + eta)) / \
           (gamma_ratio(alpha) * gamma_ratio(beta + eta) * gamma_ratio(alpha + beta + 2 * eta + rho)) \
         + (8.8995e-20 * t**(2 * beta + 2 * eta + rho) * gamma_ratio(2 * beta + eta)) / \
           (gamma_ratio(beta) * gamma_ratio(beta + eta) * gamma_ratio(2 * beta + 2 * eta + rho)) \
         + (0.0000184737 * t**(eta + 2 * rho)) / gamma_ratio(eta + 2 * rho) \
         - (1.54513e-8 * t**(alpha + eta + 2 * rho)) / gamma_ratio(alpha + eta + 2 * rho) \
         - (2.91654e-14 * t**(beta + eta + 2 * rho)) / gamma_ratio(beta + eta + 2 * rho) \
         + (9.55718e-9 * t**(3 * rho)) / gamma_ratio(3 * rho)

    return R0 + R1 + R2 + R3


t = np.linspace(0.001, 20, 1000)

plt.figure(figsize=(12, 7))


print("Calculating for fractional orders = 1...")
alpha1, beta1, eta1, rho1 = 1.0, 1.0, 1.0, 1.0

S1 = np.array([susceptible(ti, alpha1, beta1, eta1, rho1) for ti in t])
V1 = np.array([vaccinated(ti, alpha1, beta1, eta1, rho1) for ti in t])
I1 = np.array([infected(ti, alpha1, beta1, eta1, rho1) for ti in t])
R1 = np.array([recovered(ti, alpha1, beta1, eta1, rho1) for ti in t])


plt.plot(t, S1, 'b-', linewidth=2.5, label=r'$\mathcal{S}$')
plt.plot(t, V1, 'g-', linewidth=2.5, label=r'$\mathcal{V}$')
plt.plot(t, I1, 'r-', linewidth=2.5, label=r'$\mathcal{I}$')
plt.plot(t, R1, 'm-', linewidth=2.5, label=r'$\mathcal{R}$')


plt.xlabel('Time (t)', fontsize=14, fontweight='bold', fontfamily='serif')
plt.ylabel('Population', fontsize=14, fontweight='bold', fontfamily='serif')
plt.title(r'SVIR Model - Homogeneous Fractional Orders : $\alpha_\mathcal{S}= \alpha_\mathcal{V}= \alpha_\mathcal{I}= \alp


plt.legend(loc='upper left', fontsize=13, frameon=True, fancybox=True,
           facecolor='white', edgecolor='black', shadow=True, borderpad=1)

```

```
plt.grid(True, alpha=0.3, linestyle='--')
plt.xlim([0, 20])


plt.gca().set_facecolor('#f8f8f8')

plt.tight_layout()


plt.show()
```

Fractional for S

```
import numpy as np
import matplotlib.pyplot as plt
from scipy.special import gamma


def gamma_ratio(x):
    return gamma(1 + x)

def susceptible(t, alpha, beta, eta, rho):

    S0 = 500 + (0.410126 * t**alpha) / gamma_ratio(alpha)


    S1 = -((7.4824 * t**alpha) / gamma_ratio(alpha)) \
         - (0.00693498 * t**(2 * alpha)) / gamma_ratio(2 * alpha) \
         + (2.15056e-6 * t**(alpha + beta)) / gamma_ratio(alpha + beta)


    S2 = (0.126523 * t**(2 * alpha)) / gamma_ratio(2 * alpha) \
         + (0.000117266 * t**(3 * alpha)) / gamma_ratio(3 * alpha) \
         - (0.000123065 * t**(alpha + beta)) / gamma_ratio(alpha + beta) \
         + (2.11013e-6 * t**(2 * alpha + beta)) / gamma_ratio(2 * alpha + beta) \
         - (6.06029e-9 * t**(alpha + 2 * beta)) / gamma_ratio(alpha + 2 * beta) \
         + (0.367763 * t**(alpha + eta)) / gamma_ratio(alpha + eta) \
         - (0.000307595 * t**(2 * alpha + eta)) / gamma_ratio(2 * alpha + eta) \
         + (0.000301658 * t**(2 * alpha + eta) * gamma_ratio(alpha + eta)) / \
           (gamma_ratio(alpha) * gamma_ratio(eta) * gamma_ratio(2 * alpha + eta)) \
         - (2.52305e-7 * t**(3 * alpha + eta) * gamma_ratio(2 * alpha + eta)) / \
           (gamma_ratio(alpha) * gamma_ratio(alpha + eta) * gamma_ratio(3 * alpha + eta)) \
         - (5.80605e-10 * t**(alpha + beta + eta)) / gamma_ratio(alpha + beta + eta) \
         - (4.76242e-13 * t**(2 * alpha + beta + eta) * gamma_ratio(alpha + beta + eta)) / \
           (gamma_ratio(alpha) * gamma_ratio(beta + eta) * gamma_ratio(2 * alpha + beta + eta))

    S3 = -((0.00213943 * t**(3 * alpha)) / gamma_ratio(3 * alpha)) \
         - (1.9829e-6 * t**(4 * alpha)) / gamma_ratio(4 * alpha) \
         - (0.00003708 * t**(2 * alpha + beta)) / gamma_ratio(2 * alpha + beta) \
         - (7.1977e-8 * t**(3 * alpha + beta)) / gamma_ratio(3 * alpha + beta) \
         + (3.46797e-7 * t**(alpha + 2 * beta)) / gamma_ratio(alpha + 2 * beta) \
         - (5.93509e-9 * t**(2 * alpha + 2 * beta)) / gamma_ratio(2 * alpha + 2 * beta) \
         + (1.70779e-11 * t**(alpha + 3 * beta)) / gamma_ratio(alpha + 3 * beta) \
         - (0.000606852 * t**(2 * alpha + eta)) / gamma_ratio(2 * alpha + eta) \
         - (0.00550349 * t**(2 * alpha + eta) * gamma_ratio(alpha + eta)) / \
           (gamma_ratio(alpha) * gamma_ratio(eta) * gamma_ratio(2 * alpha + eta)) \
         + (0.0000104025 * t**(3 * alpha + eta)) / gamma_ratio(3 * alpha + eta) \
         - (5.10086e-6 * t**(3 * alpha + eta) * gamma_ratio(alpha + eta)) / \
           (gamma_ratio(alpha) * gamma_ratio(eta) * gamma_ratio(3 * alpha + eta)) \
         - (5.10085e-6 * t**(3 * alpha + eta) * gamma_ratio(2 * alpha + eta)) / \
           (gamma_ratio(2 * alpha) * gamma_ratio(eta) * gamma_ratio(3 * alpha + eta)) \
         + (9.20618e-6 * t**(3 * alpha + eta) * gamma_ratio(2 * alpha + eta)) / \
           (gamma_ratio(alpha) * gamma_ratio(alpha + eta) * gamma_ratio(3 * alpha + eta)) \
         + (4.26633e-9 * t**(4 * alpha + eta) * gamma_ratio(2 * alpha + eta)) / \
           (gamma_ratio(alpha) * gamma_ratio(alpha + eta) * gamma_ratio(4 * alpha + eta)) \
         + (4.26632e-9 * t**(4 * alpha + eta) * gamma_ratio(3 * alpha + eta)) / \
           (gamma_ratio(2 * alpha) * gamma_ratio(alpha + eta) * gamma_ratio(4 * alpha + eta)) \
         + (4.26634e-9 * t**(4 * alpha + eta) * gamma_ratio(3 * alpha + eta)) / \
           (gamma_ratio(alpha) * gamma_ratio(2 * alpha + eta) * gamma_ratio(4 * alpha + eta)) \
         + (7.48377e-7 * t**(alpha + beta + eta)) / gamma_ratio(alpha + beta + eta) \
         - (2.78076e-9 * t**(2 * alpha + beta + eta)) / gamma_ratio(2 * alpha + beta + eta) \
         + (1.58179e-9 * t**(2 * alpha + beta + eta) * gamma_ratio(alpha + beta + eta)) / \
           (gamma_ratio(alpha + beta) * gamma_ratio(eta) * gamma_ratio(2 * alpha + beta + eta)) \
         + (3.59415e-11 * t**(2 * alpha + beta + eta) * gamma_ratio(alpha + beta + eta)) / \
           (gamma_ratio(alpha) * gamma_ratio(beta + eta) * gamma_ratio(2 * alpha + beta + eta)) \
```

```
        + (8.05297e-15 * t**(3 * alpha + beta + eta) * gamma_ratio(alpha + beta + eta)) / \
          (gamma_ratio(alpha) * gamma_ratio(beta + eta) * gamma_ratio(3 * alpha + beta + eta)) \
        - (1.323e-12 * t**(3 * alpha + beta + eta) * gamma_ratio(2 * alpha + beta + eta)) / \
          (gamma_ratio(alpha + beta) * gamma_ratio(alpha + eta) * gamma_ratio(3 * alpha + beta + eta)) \
        + (8.05297e-15 * t**(3 * alpha + beta + eta) * gamma_ratio(2 * alpha + beta + eta)) / \
          (gamma_ratio(2 * alpha) * gamma_ratio(beta + eta) * gamma_ratio(3 * alpha + beta + eta)) \
        - (1.79835e-12 * t**(3 * alpha + beta + eta) * gamma_ratio(2 * alpha + beta + eta)) / \
          (gamma_ratio(alpha) * gamma_ratio(alpha + beta + eta) * gamma_ratio(3 * alpha + beta + eta)) \
        + (1.63502e-12 * t**(alpha + 2 * beta + eta)) / gamma_ratio(alpha + 2 * beta + eta) \
        + (1.58179e-12 * t**(alpha + 2 * beta + eta) * gamma_ratio(beta + eta)) / \
          (gamma_ratio(beta) * gamma_ratio(eta) * gamma_ratio(alpha + 2 * beta + eta)) \
        - (1.323e-15 * t**(2 * alpha + 2 * beta + eta) * gamma_ratio(alpha + beta + eta)) / \
          (gamma_ratio(beta) * gamma_ratio(alpha + eta) * gamma_ratio(2 * alpha + 2 * beta + eta)) \
        - (2.49725e-18 * t**(2 * alpha + 2 * beta + eta) * gamma_ratio(alpha + 2 * beta + eta)) / \
          (gamma_ratio(alpha + beta) * gamma_ratio(beta + eta) * gamma_ratio(2 * alpha + 2 * beta + eta)) \
        + (1.34205e-15 * t**(2 * alpha + 2 * beta + eta) * gamma_ratio(alpha + 2 * beta + eta)) / \
          (gamma_ratio(alpha) * gamma_ratio(2 * beta + eta) * gamma_ratio(2 * alpha + 2 * beta + eta)) \
        - (2.49725e-21 * t**(alpha + 3 * beta + eta) * gamma_ratio(2 * beta + eta)) / \
          (gamma_ratio(beta) * gamma_ratio(beta + eta) * gamma_ratio(alpha + 3 * beta + eta)) \
        - (0.0180333 * t**(alpha + 2 * eta)) / gamma_ratio(alpha + 2 * eta) \
        + (0.0000150829 * t**(2 * alpha + 2 * eta)) / gamma_ratio(2 * alpha + 2 * eta) \
        + (0.0000150829 * t**(2 * alpha + 2 * eta) * gamma_ratio(alpha + eta)) / \
          (gamma_ratio(alpha) * gamma_ratio(eta) * gamma_ratio(2 * alpha + 2 * eta)) \
        - (0.0000147918 * t**(2 * alpha + 2 * eta) * gamma_ratio(alpha + 2 * eta)) / \
          (gamma_ratio(alpha) * gamma_ratio(2 * eta) * gamma_ratio(2 * alpha + 2 * eta)) \
        - (1.26153e-8 * t**(3 * alpha + 2 * eta) * gamma_ratio(2 * alpha + eta)) / \
          (gamma_ratio(alpha) * gamma_ratio(alpha + eta) * gamma_ratio(3 * alpha + 2 * eta)) \
        + (1.23718e-8 * t**(3 * alpha + 2 * eta) * gamma_ratio(2 * alpha + 2 * eta)) / \
          (gamma_ratio(alpha) * gamma_ratio(alpha + 2 * eta) * gamma_ratio(3 * alpha + 2 * eta)) \
        + (1.23718e-8 * t**(3 * alpha + 2 * eta) * gamma_ratio(alpha + eta) * gamma_ratio(2 * alpha + 2 * eta)) / \
          (gamma_ratio(alpha)**2 * gamma_ratio(eta) * gamma_ratio(alpha + 2 * eta) * gamma_ratio(3 * alpha + 2 * eta)) \
        - (1.03477e-11 * t**(4 * alpha + 2 * eta) * gamma_ratio(2 * alpha + eta) * gamma_ratio(3 * alpha + 2 * eta)) / \
          (gamma_ratio(alpha)**2 * gamma_ratio(alpha + eta) * gamma_ratio(2 * alpha + 2 * eta) * gamma_ratio(4 * alpha +
        + (2.847e-11 * t**(alpha + beta + 2 * eta)) / gamma_ratio(alpha + beta + 2 * eta) \
        + (2.847e-11 * t**(alpha + beta + 2 * eta) * gamma_ratio(beta + eta)) / \
          (gamma_ratio(beta) * gamma_ratio(eta) * gamma_ratio(alpha + beta + 2 * eta)) \
        - (2.38121e-14 * t**(2 * alpha + beta + 2 * eta) * gamma_ratio(alpha + beta + eta)) / \
          (gamma_ratio(beta) * gamma_ratio(alpha + eta) * gamma_ratio(2 * alpha + beta + 2 * eta)) \
        - (2.38121e-14 * t**(2 * alpha + beta + 2 * eta) * gamma_ratio(alpha + beta + eta)) / \
          (gamma_ratio(alpha) * gamma_ratio(beta + eta) * gamma_ratio(2 * alpha + beta + 2 * eta)) \
        + (2.33525e-14 * t**(2 * alpha + beta + 2 * eta) * gamma_ratio(alpha + beta + 2 * eta)) / \
          (gamma_ratio(alpha) * gamma_ratio(beta + 2 * eta) * gamma_ratio(2 * alpha + beta + 2 * eta)) \
        + (2.33525e-14 * t**(2 * alpha + beta + 2 * eta) * gamma_ratio(beta + eta) * gamma_ratio(alpha + beta + 2 * eta))
          (gamma_ratio(alpha) * gamma_ratio(beta) * gamma_ratio(eta) * gamma_ratio(beta + 2 * eta) * gamma_ratio(2 * alph
        - (1.95319e-17 * t**(3 * alpha + beta + 2 * eta) * gamma_ratio(alpha + beta + eta) * gamma_ratio(2 * alpha + beta
          (gamma_ratio(alpha) * gamma_ratio(beta) * gamma_ratio(alpha + eta) * gamma_ratio(alpha + beta + 2 * eta) * gamm
        - (1.95319e-17 * t**(3 * alpha + beta + 2 * eta) * gamma_ratio(alpha + beta + eta) * gamma_ratio(2 * alpha + beta
          (gamma_ratio(alpha)**2 * gamma_ratio(beta + eta) * gamma_ratio(alpha + beta + 2 * eta) * gamma_ratio(3 * alpha
        - (4.4947e-20 * t**(alpha + 2 * beta + 2 * eta) * gamma_ratio(2 * beta + eta)) / \
          (gamma_ratio(beta) * gamma_ratio(beta + eta) * gamma_ratio(alpha + 2 * beta + 2 * eta)) \
        - (3.68678e-23 * t**(2 * alpha + 2 * beta + 2 * eta) * gamma_ratio(2 * beta + eta) * gamma_ratio(alpha + 2 * beta
          (gamma_ratio(alpha) * gamma_ratio(beta) * gamma_ratio(beta + eta) * gamma_ratio(2 * beta + 2 * eta) * gamma_rat

    return S0 + S1 + S2 + S3


t_values = np.linspace(0, 20, 100)


orders = [1.0, 0.9, 0.8, 0.7, 0.6, 0.5]


colors = ['blue', 'green', 'red', 'purple', 'orange', 'brown']
markers = ['o', 's', '^', 'D', 'v', 'X']
line_styles = ['-', '--', '-.', ':', '-', '-']


labels = [f'$\\alpha_\\mathcal{{S}} = \\alpha_\\mathcal{{V}} = \\alpha_\\mathcal{{I}} = \\alpha_\\mathcal{{R}} = {order}$'


plt.figure(figsize=(12, 8))


for i, order in enumerate(orders):
    S_values = []
    for t in t_values:
        S_val = susceptible(t, order, order, order, order)
```

```
        S_values.append(S_val)

    plt.plot(t_values, S_values,
             color=colors[i],
             linestyle=line_styles[i],
             linewidth=2.5,
             marker=markers[i],
             markevery=10,
             markersize=8,
             label=labels[i])


plt.title(r'Susceptible Population $\mathcal{S}(t)$ for Different Fractional Orders',
          fontsize=16, fontweight='bold', pad=20, color='#2c3e50')
plt.xlabel('Time (t)', fontsize=14, fontweight='bold')
plt.ylabel('$\mathcal{S}(t)$ - Susceptible Population', fontsize=14, fontweight='bold')
plt.grid(True, alpha=0.3, linestyle='--')
plt.legend(loc='upper right', fontsize=12)
plt.xlim(0, 20)
plt.ylim(400, 510)

plt.tight_layout()
plt.gca().set_facecolor('#f8f8f8')
plt.show()


print("\n" + "="*60)
print("Susceptible population at t=1 for different fractional orders:")
print("="*60)
for i, order in enumerate(orders):
    S_final = susceptible(1, order, order, order, order)
    print(f"α = {order}: S(1) = {S_final:.6f}")
print("="*60)
```
